# *Klebsiella pneumoniae* O-polysaccharide biosynthesis highlights the diverse organization of catalytic modules in ABC transporter-dependent glycan assembly

**DOI:** 10.1016/j.jbc.2024.107420

**Published:** 2024-05-28

**Authors:** Steven D. Kelly, Danielle M. Williams, Shawna Zhu, Taeok Kim, Manas Jana, Jeremy Nothof, V. Narasimharao Thota, Todd L. Lowary, Chris Whitfield

**Affiliations:** 1 Department of Molecular and Cellular Biology, University of Guelph, Guelph, Ontario, Canada; 2 Department of Chemistry, University of Alberta, Edmonton, Alberta, Canada; 3 Institute of Biological Chemistry, Academia Sinica, Taipei, Taiwan; 4 Institute of Biochemical Sciences, National Taiwan University, Taipei, Taiwan

**Keywords:** *Klebsiella*, O-antigens, O4, O7, polysaccharide, polymerization, termination, *in vitro*

## Abstract

*Klebsiella pneumoniae* provides influential prototypes for lipopolysaccharide O antigen (OPS) biosynthesis in Gram-negative bacteria. Sequences of OPS-biosynthesis gene clusters in serotypes O4 and O7 suggest fundamental differences in the organization of required enzyme modules compared to other serotypes. Furthermore, some required activities were not assigned by homology shared with characterized enzymes. The goal of this study was therefore to resolve the serotype O4 and O7 pathways to expand our broader understanding of glycan polymerization and chain termination processes. The O4 and O7 antigens were produced from cloned genetic loci in recombinant *Escherichia coli*. Systematic *in vivo* and *in vitro* approaches were then applied to assign each enzyme in each of the pathways, defining the necessary components for polymerization and chain termination. OPS assembly is accomplished by multiprotein complexes formed by interactions between polymerase components variably distributed in single and multimodule proteins. In each complex, a terminator function is present in a protein containing a characteristic coiled-coil molecular ruler, which determines glycan chain length. In serotype O4, we discovered a CMP-α-3-deoxy-ᴅ-*manno*-octulosonic acid-dependent chain-terminating glycosyltransferase that is the founding member of a new glycosyltransferase family (GT137) and potentially identifies a new glycosyltransferase fold. The O7 OPS is terminated by a methylphosphate moiety, like the *K. pneumoniae* O3 antigen, but the methyltransferase-kinase enzyme pairs responsible for termination in these serotypes differ in sequence and predicted structures. Together, the characterization of O4 and O7 has established unique enzyme activities and provided new insight into glycan-assembly strategies that are widely distributed in bacteria.

*Klebsiella pneumoniae* is a species of Gram-negative commensal bacteria found in the human mouth, gut and oropharynx. “Classical” isolates can cause opportunistic infections (typically in immunocompromised individuals), including bacteremia, urinary tract infections, and pneumonia (1). However, the emergence of both highly antibiotic-resistant clades and “hypervirulent” isolates capable of causing community acquired infections in healthy individuals creates an urgent clinical concern (2, 3, 4). Treating multidrug-resistant infections is challenging and is often left to last resort antibiotics. However, strategies for vaccination or passive immunization have been developed and offer promise. Capsular polysaccharides (K antigen) and lipopolysaccharide (LPS) O antigens (O polysaccharides; OPS) are important cell surface structures involved in cell-integrity, immune evasion and virulence, and they both offer viable antigen candidates that can be exploited for immunotherapeutic initiatives. OPS have certain advantages because the limited diversity of *K. pneumoniae* OPS structures (∼13; (5, 6)) compared to the 77 recognized K antigens (7). Certain serotypes are highly represented in clinical isolates, with recent assessments estimating that serotype O1, the O2 and O3 serogroups (groups of serotypes possessing related OPS structures with some shared epitopes), together with serotype O5, constitute at least 75% of global isolates (5, 8, 9, 10, 11). Immunotherapeutic strategies targeting individual O antigens, and combinations of O antigens have proved effective in animal challenge experiments and serum killing tests with some isolates (12, 13, 14, 15, 16, 17, 18). However, capsular polysaccharides reduce protection from OPS-directed antibodies, particularly in hypervirulent isolates and antibodies against selected capsular types are protective in animal models (19, 20).

The development of immunotherapeutic strategies involving contemporary glycoengineering methods requires detailed systematic knowledge of the relevant polysaccharide structures and the genes required for their production. Our group has focused on establishing this essential foundation. As a result, the OPS-biosynthesis systems in *K. pneumoniae* serotypes O1, O2, O3, O5, and O12 serotypes are now well understood, and they have provided fundamental prototypes for the biosynthesis of OPS in other Gram-negative bacteria. All of the known *K. pneumoniae* O-antigens are produced by an ATP-binding cassette (ABC) transporter-dependent process present in many species (reviewed in Ref. (21)). In this process, the OPS is polymerized as a polyisoprenoid lipid-linked intermediate at the membrane–cytoplasm interface and exported to the periplasm by the system-defining ABC transporter composed of two subunits each of the Wzm (transmembrane) and Wzt (nucleotide-binding domain) proteins. The completed OPS is then transferred from the polyisoprenoid lipid carrier and ligated to the lipid A–core oligosaccharide component of LPS to form the mature LPS molecule, which is then translocated to the outer leaflet of the outer membrane.

Biosynthesis of all *K. pneumoniae* OPS (and OPS in many other species) begins by the transfer of *N*-acetylglucosamine (GlcNAc)-1-phosphate to the undecaprenyl-phosphate (und-P) lipid carrier, performed by a UDP-GlcNAc-dependent phosphoglycosyltransferase enzyme (WecA) encoded by a gene in the enterobacterial common antigen locus. Following initiation, an adapter region composed of one or two sugars is added by glycosyltransferases (GTs), to create an acceptor that can be extended by a “polymerase” containing multiple GT modules. Studies with *K. pneumoniae* have identified two fundamentally different strategies for engagement of the nascent OPS by the ABC transporter and the details of these processes have a critical effect on the determination of OPS chain length. One strategy is represented by serogroup O3 and serotype O5, while the other has serotype O1 and the O2 serogroup as prototypes.

OPS belonging to *K. pneumoniae* serotype O3 subtypes and O5 have been comprehensively investigated in *Escherichia coli*, where the same *rfb* (OPS biosynthesis) genetic locus has been subject to exchange by horizontal gene transfer (*K. pneumoniae* O3 = *E. coli* O9 and *K. pneumoniae* O5 = *E. coli* O8) (22) (Fig. 1*A*). These serotypes were the first reported examples of a polymerase–terminator model for the biosynthesis of bacterial glycans that has been extended to many species and different types of glycans (23). The O3 and O5 assembly systems each contain a single polymerase enzyme (WbdA) containing two (O3) or three (O5) GDP-mannose-dependent mannosyl GT modules; one module is present for each of the linkage type in the product (24). The polysaccharide chains are terminated by methyl (O5) or phospho-methyl (O3) moieties added by the terminating enzyme (WbdD). In WbdD proteins, an extended coiled-coil molecular ruler separates the N-terminal catalytic sites from a C-terminal region containing a membrane-associated amphipathic helix and a recruitment site for the polymerase (25, 26). The occupancy of the polymerase recruitment site and the length of the coiled-coil region determine the size distribution of the OPS (27, 28). The coiled-coil spacer puts a physical separation between the polymerization and termination catalytic modules, so only polysaccharides that have reached a minimum length are terminated. In *K. pneumoniae* serotype O12, polymerization and termination are performed by a single enzyme, WbbB (Fig. 1*A*). Two C-terminal GTs polymerize the disaccharide repeat unit region of the O12 polysaccharide (29) and an N-terminal GT terminates polymerization by installing a β-linked 3-deoxy-D-*manno*-oct-2-ulosonic acid (Kdo) residue (30). In each system, only terminated OPS chains are substrates for serotype-specific ABC transporters. A carbohydrate-binding module (CBM) attached to the nucleotide-binding domain component of the transporter specifically recognizes the terminal moiety (31, 32, 33), providing a quality assurance checkpoint to ensure the OPS is fully mature and ready for export. The structure of a related ABC transporter has been reported and a model for the transport process has been proposed (34, 35, 36).

**Figure 1:**
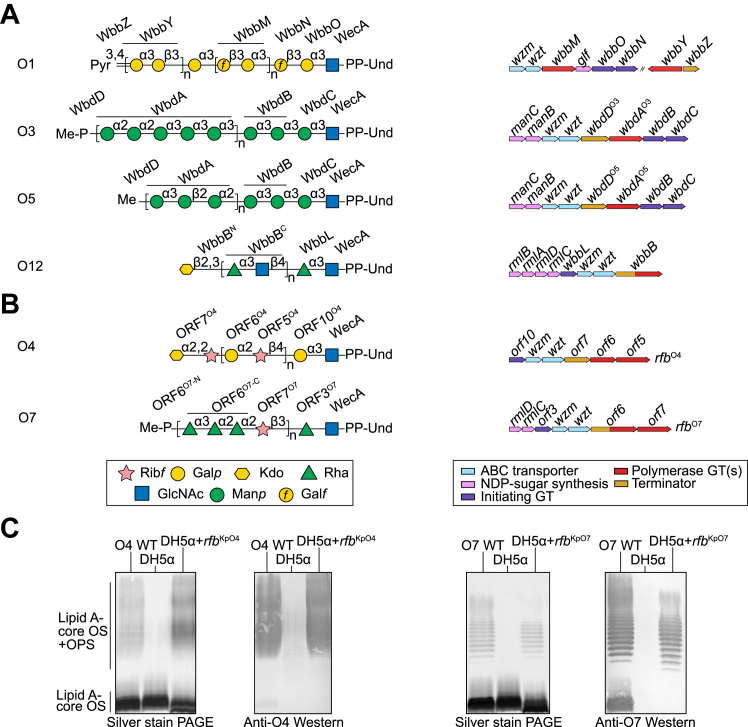
**Functional assignments of gene products encoded by *rfb* loci responsible for the production of *K. pneumoniae* OPSs.***A*, functions in O serotypes where the assembly pathways were established previously. The *orfs* are colored according to their roles in biosynthesis. The structures are presented as the corresponding completed und-PP-linked OPSs, prior to export and ligation. *B*, functional assignment of gene products for the biosynthesis of the O4 (LT174605) and O7 (MN17377) serotypes, reported in this study. *C*, SDS-PAGE and western immunoblots illustrating the production of OPS-substituted LPS molecules in *E. coli* DH5α transformed with plasmids containing the cloned *rfb*^KpO4^ (*orf10–5*; pWQ1146) or *rfb*^KpO7^ (*orf3-7*: pWQ1151) gene clusters. The *K. pneumoniae* reference strains for serotypes O4 (1702) and O7 (264–1) are also shown. All SD-PAGE and blots were performed in triplicate.

The biosynthesis strategy for OPSs belonging to serotype O1 and the O2 group (Fig. 1*A*) differ fundamentally in the interface between polymerization and export. All are built on the same (O2a) backbone with a disaccharide repeat unit whose biosynthetic sequence is established by the polymerase WbbM, which contains 2 GT modules (37). Diversification of this backbone is dictated by genes located outside the *rfb*^O2a^ locus. In serotypes O2afg and O2aeh, side-chain galactose residues are added after export to the periplasm (38). In contrast, in serotype O1, the modification occurs pre-export and includes extension of the O2a backbone by another dual-GT polymerase (WbbY) creating a region with a different repeat-unit (the O1 antigen) (39), as well as installation of a terminating pyruvate group on a portion of the chains (40). Unlike the O3, O5 and O12 OPSs, the terminating pyruvyltransferase enzyme in O1 only diversifies the structure; it is not required for chain-length determination and the ABC transporter exports chains with and without the terminal pyruvate (40). Investigation of the O2a models showed that distribution of OPS chain lengths is dictated by the stoichiometry of the ABC transporter:biosynthesis complex and chain extension and export are obligatorily coupled (41). The ABC transporter shared by O1 and O2 group isolates lacks a CBM to confer substrate specificity and, in a genetically engineered recombinant, can also export the O3a antigen (41).

While *K. pneumoniae* OPS systems have provided influential prototypes, variation is evidently possible in enzyme formats and distribution of required catalytic modules. We are interested in the limits of this variation and the consequences for the assembly of glycans in other bacteria. The sequences of the *rfb* (OPS biosynthesis) gene loci from serotypes O4 and O7 suggest key differences from the currently characterized systems. At least one catalytic module of the “polymerase” (a ribofuranosyltransferase) from each system is provided by an independent polypeptide (42). Furthermore, some catalytic components could not be identified from the sequence data, suggesting the existence of novel enzymes or structures. The goal of the current study was to resolve the details of the O4 and O7 assembly pathways as part of our broader efforts to further fundamental insights into the architectural principles of glycan biosynthesis complexes in bacteria.

## Results

### Cloning the O4 OPS-biosynthesis gene cluster

The *K. pneumoniae* O4 OPS structure contains a disaccharide repeat unit of galactopyranose and ribofuranose, with a non-reducing terminal α-linked Kdo residue (43) (Fig. 1*B*). The candidate genetic locus directing O4 synthesis deposited in GenBank (LT174605) (designated here as *rfb*^O4^, following the classical *rfb* name for OPS gene clusters) contains six predicted *orf*s (Fig. 1*B*). To first confirm the overall function of this gene cluster, a fragment encompassing the six *orfs* was cloned into pACYC184 to make pWQ1146, and transformed into *E. coli* DH5α. The host is derived from *E. coli* K-12, which does not make native OPS due to mutations in the gene (*wbbL*) encoding a rhamnosyltransferase that catalyzes addition of the second sugar in polyprenol-linked OPS repeat-unit synthesis (44). UDP-galactose and phosphoribosylpyrophosphate (PRPP) are the activated donors required for O4 biosynthesis and they are produced by enzymes encoded by genes in the *E. coli* DH5α chromosome (45, 46). SDS-PAGE of whole-cell lysates from transformants containing pWQ1146 showed the presence of OPS, evident from the characteristic high-molecular-weight material visible in the silver-stained gel (Fig. 1*C*). Western immunoblotting using rabbit antiserum generated against wildtype O4 cells revealed robust reactivity to the recombinant OPS, consistent with it being authentic O4 OPS. This conclusion was subsequently confirmed by biochemical experiments. Of the six gene products in the cluster, biochemical data was available only for ORF5^O4^, the PRPP-dependent ribofuranosyltransferase participating in the biosynthesis of the disaccharide repeat-unit region of the OPS (42). As expected, the locus contains *wzm* and *wzt* genes, encoding the subunits of the ABC transporter. The *orf10*^O4^ and *orf6*^O4^ genes are predicted to produce GTs, but *orf7*^O4^ encodes a protein whose function could not be predicted based on sequence. The roles of these ORFs were established by a combination of genetic and biochemical experiments.

### ORF10^O4^ is a galactopyranosyl transferase that catalyzes the first committed step in O4 OPS biosynthesis

Following the synthesis of undPP-GlcNAc by WecA, this compound is committed to the OPS-biosynthesis pathway by the addition of an adapter sugar (or sugars) that is also present in the repeat unit. The native O4 OPS structure (43) implicates a galactopyranose (Gal*p*) residue as the candidate adapter. ORF6^O4^ and ORF10^O4^ are predicted GTs and both offer potential candidates for this step. BLAST searches with ORF10^O4^ revealed it to be highly similar to a galactosyltransferase (WbbO) known to participate in the synthesis of the adapter region in *K. pneumoniae* O2a OPS (37). ORF10^O4^ and WbbO share 40% identity/63% similarity (Fig. S1), making ORF10^O4^ the most likely candidate GT for O4 OPS adapter synthesis. To test this hypothesis, ORF10^O4^ was cloned and the plasmid (pWQ1148) was transformed into *E. coli* DH5α carrying a version of the *K. pneumoniae rfb*^O2a^ gene cluster with *wbbO* deleted. *orf10*^O4^ complemented the *wbbO* deletion to restore O2a OPS production (Fig. S2*A*). This data confirms that ORF10^O4^ performs the same function as WbbO: adding a Gal*p* residue to undPP-GlcNAc (37).

### ORF6^O4^ is a UDP-Gal*p*-dependent GT involved in the synthesis of the repeat-unit region of O4 OPS

**Figure 2:**
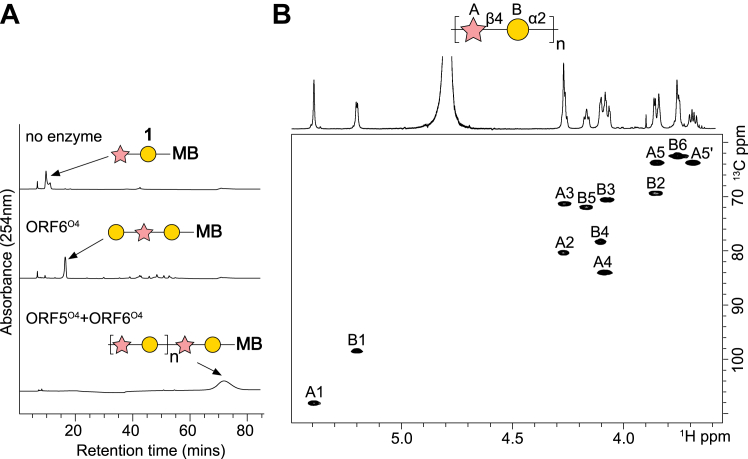
**ORF6**^**O4**^**is a UDP-Gal*p-*dependent GT that participates in O4 OPS polymerization.***A*, *in vitro* reactions were performed using the O4 repeat unit mimic **1** as an acceptor, and reactions included either ORF6^O4^, or a combination of ORF6^O4^ and ORF5^O4^. Reactions were performed in triplicate at 30 °C for 30 min with UDP-Gal*p* and PRPP, the Gal*p* and Rib*f* donors respectively. Reactions were stopped by the addition of an equal volume of acetonitrile and mixtures were separated by HPLC. *B*, the polymerization reaction was scaled up and the product was purified and analyzed by NMR spectroscopy. The ^1^H,^13^C HSQC spectrum shows chemical shifts identical to the authentic O4 OPS (determined previously (43)) (Table S2). NMR spectroscopy was performed once. MB identifies the octyl methoxybenzamide aglycone tag of the acceptor.

ORF6^O4^ was hypothesized to participate in the biosynthesis of the disaccharide repeat-unit domain or termination of the OPS. The Carbohydrate Active enZYmes (CAZY) database classifies ORF6^O4^ as a GT4 family member, based on sequence similarity. GT4 enzymes possess a GTB fold and utilize a range of NDP-linked substrates, including UDP-Gal*p*, UDP-Glc*p*, and UDP-Glc*p*NAc. The α-configuration of UDP-Gal*p* and the α-Gal*p* linkage in the O4 OPS is consistent with a retaining mechanism, like all other GT4 enzymes. A role in polymerization seemed likely and this was tested using *in vitro* GT reactions containing purified ORF6^O4^, a defined synthetic repeat unit mimic **1** and UDP-Gal*p* donor. Compound **1** is composed of a β-D-Rib*f*-(1→4)-α-D-Gal*p* disaccharide linked to a methoxybenzamide aglycone (Table S1). Following the reaction, the contents of the *in vitro* reaction mixtures were analyzed by HPLC and reactions containing ORF6^O4^ showed depletion of the peak corresponding to **1**, and accumulation of a new later-eluting species (Fig. 2*A*). Mass spectrometry of the reaction mixture confirmed the addition of a hexose onto **1** (Fig. S3), consistent with ORF6^O4^ as the galactosyltransferase for O4 polymerization. To unequivocally demonstrate the polymerization process, a reaction containing **1** was performed with ORF6^O4^ and ORF5^O4^, together with their corresponding donor substrates, UDP-Gal*p* and PRPP. The HPLC chromatogram showed the depletion of **1** and the appearance of a late eluting (and presumably high molecular weight) species. This product was purified from a scaled-up reaction and analyzed by NMR spectroscopy. Data from two-dimensional NMR experiments identified a structure identical to the repeat-unit region of authentic O4 OPS (43) (Fig. 2*B*, and Table S2). The results establish ORF5^O4^ and ORF6^O4^ as providing the two required catalytic modules for the O4 OPS polymerization.

### ORF7^O4^ terminates O4 polymerization and is the founding member of the GT137 family

**Figure 3:**
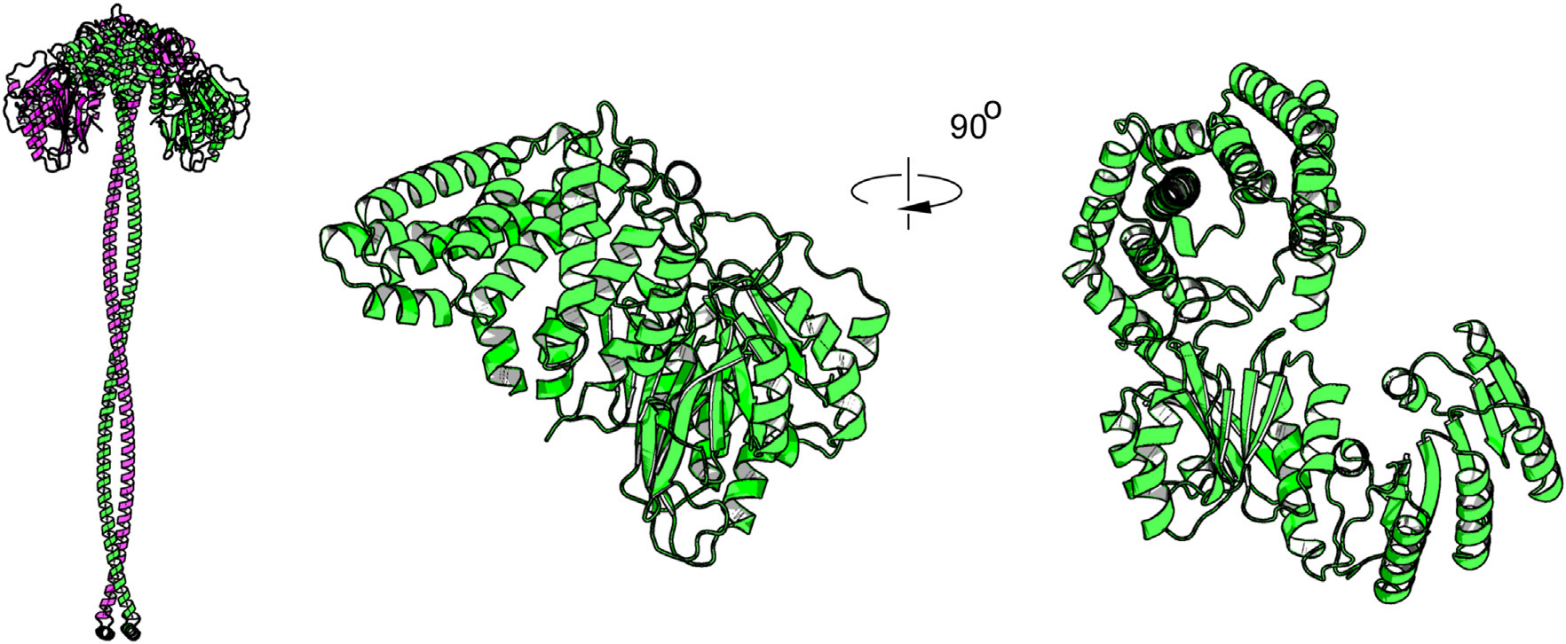
**AlphaFold model of ORF7**^**O4**^**.** ORF7^O4^ was modeled as a dimer with protomers in *green* and *pink*. Modeling was performed as a dimer as coiled-coils form oligomers, usually dimers or trimers (86). A dimer was arbitrarily chosen for the simplicity of AlphaFold modeling, but the exact oligomeric state of ORF7^O4^ is unknown. An expanded view of the CMP-Kdo-dependent GT module of one of the protomers is shown to the *right* with two views shown.

With the initiating and polymerizing enzymes of O4 characterized, ORF7^O4^ provided the only remaining candidate for the addition of the terminal α-2,2-linked Kdo residue found in the natural product. The only α-Kdo transferase that has been biochemically and structurally defined is WaaA, which (in *E. coli*) adds two Kdo residues to the LPS lipid A precursor, lipid IV_A_, to generate Kdo_2_-Lipid A (47, 48). ORF7^O4^ shares no similarity with WaaA. Furthermore, a search of the Conserved Domain Database returned no functional predictions for ORF7^O4^ and the CAZy database did not have the enzyme classified as a GT. Analysis of ORF7^O4^ with DeepCoil predicted a C-terminal coiled-coil, a structure consistently found in glycan-terminating enzymes (23). The prototypes are provided by WbdD in serotypes O5 and the O3 group (27, 28) and WbbB in O12 (29). AlphaFold modeling of ORF7^O4^ revealed an additional tetratricopeptide repeat-like α-helical bundle preceding the coiled-coil, a feature that has not been described in other glycan-terminating enzymes. The model reveals an N-terminal domain comprised of two β/α/β domains that superficially resemble the Rossmann-like domains in the GT-B glycosyltransferase fold (Fig. 3). However, the AlphaFold structure possessed key differences from the canonical GT-B fold topology; most notably, the β/α/β domains contained a switch in the orientation of central β-sheet (compared to typical GT-B Rossmann folds) (Fig. S4). This altered orientation, located after the fourth strand, results in a topology with one antiparallel strand in the N-terminal domain, and two in the C-terminal domain. This creates a structure that more closely resembles the β-sheet organization of methyltransferase enzymes (49), than GTs. Indeed, comparison of the three-dimensional structure of the ORF7^O4^ model to experimental structures in DALI revealed no similarity to known GTs, and only partial similarity to methyltransferases. The closest was the PrmA ribosomal protein L11 methyltransferase (3cjt), which aligned in only one of the β/α/β domains with a relatively poor Z-score of 12.2 and RMSD of 5.8 Å.

**Figure 4:**
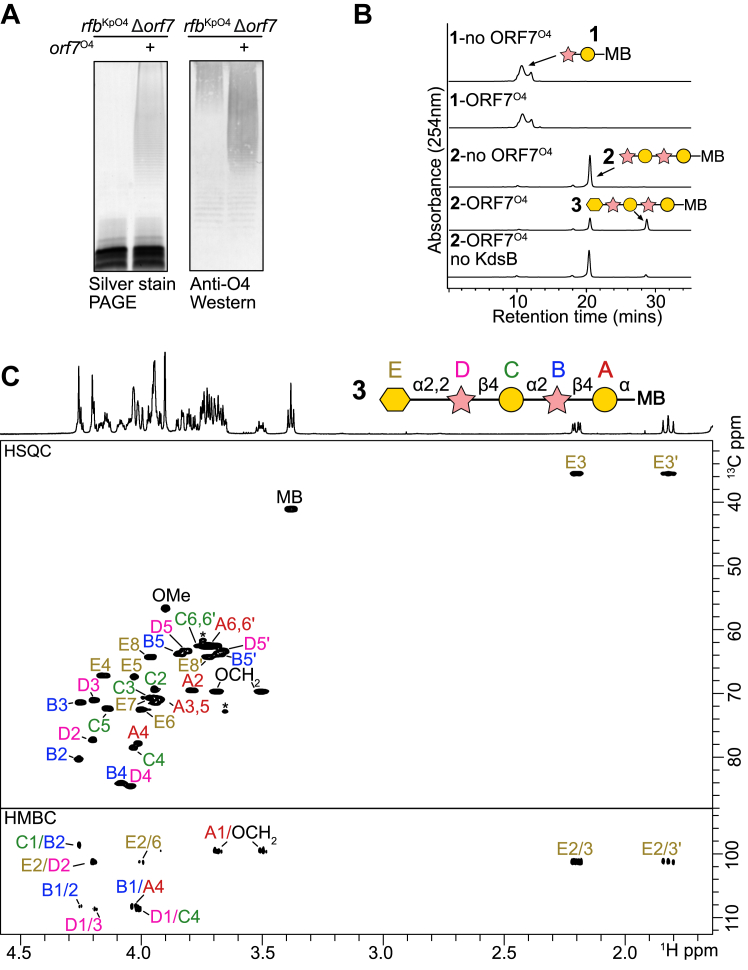
**Biochemical validation of ORF7**^**O4**^**as an α-(2→2)-Kdo transferase.***A*, Whole-cell lysates of *E. coli* DH5α cells transformed with the *rfb*^KpO4^Δ*orf7* plasmid alone, or together with the corresponding complementing plasmid expressing ORF7^O4^, were analyzed by SDS-PAGE and western blotting with rabbit O4 antiserum. The absence of silver-stained material and accumulation of high molecular weight material detected by the western immunoblot in the *orf7*^O4^ deletion is the phenotype expected from an inability to export the und-PP-linked glycan for ligation to LPS. SDS-PAGE and immunoblots were performed using biological triplicates. *B*, HPLC analysis of the contents of *in vitro* reactions using ORF7^O4^ and repeat unit mimics **1** or **2**. MB identifies the octyl-methoxybenzamide aglycone tag of the acceptor. Reactions were performed in triplicate at 4 °C overnight, after which reactions were stopped by the addition of an equal volume of acetonitrile, prior to separation by HPLC. No ORF7^O4^ activity was observed using **1**, but a later migrating species was detected in reactions with **2**. Mass spectrometry showed a new product consistent with the addition of Kdo (Fig. S7). *C*, the *in vitro* reaction containing acceptor **2** was scaled up and product **3** was purified and analyzed by NMR spectroscopy. Shown in the *upper* panel is the ^1^H,^13^C HSQC spectrum with the assignment of the product (chemical shifts in Table S4). Shown in the *lower* panel is the ^1^H,^13^C HMBC spectrum used to assign the linkage between sugar units, which confirmed the (2→2)-linkage of Kdo to Rib*f* (D). An asterisk marks an unknown contaminant.

A biochemical approach was taken to unequivocally confirm the hypothesized CMP-Kdo-dependent GT activity of ORF7^O4^. ORF7^O4^ localized primarily to the membrane (Fig. S5), and efforts to purify soluble enzyme resulted in inactivated protein, so membranes overexpressing ORF7^O4^ were isolated and used as a source of enzyme. *In vitro* reactions contained acceptor (**1**) and the donor substrate CMP-Kdo was generated *in situ* with KdsB as described previously (50), due to the inherent short lifespan of CMP-Kdo in solution (51). Initially, acceptor **1** was used as an acceptor because the Kdo in the authentic O4 OPS structure is linked to a terminal Rib*f*. However, no reaction products were detected in the HPLC chromatogram or MS spectra of these reaction mixtures. We reasoned that **1** may be too short an acceptor for ORF7^O4^, either by creating steric hindrance in the active site due to the aglycone portion of **1** or by being too short to be properly bound by the enzyme. Therefore, **1** was extended enzymatically by adding one repeat unit to make a new acceptor, compound **2** (methoxybenzamide-linked tetrasaccharide [β-D-Rib*f*-(1→4)-α-D-Gal*p*-(1→2)-β-D-Rib*f*-(1→4)-α-D-Gal*p*]). Acceptor **2** was purified and its structure was confirmed by NMR spectroscopy (Fig. S6, and Tables S1 and S3). Enzyme reactions were then performed using membranes containing ORF7^O4^ and **2**, resulting in a new, later migrating species in the HPLC chromatogram (Fig. 4*B*). Production of this compound was dependent on the presence of CMP-Kdo (*via* the addition of KdsB). The corresponding MS data was consistent with the addition of Kdo (exact mass = 220) to **2** (exact mass= 868) (Fig. S7). To unequivocally establish Kdo transfer and confirm the product linkage, the ORF7^O4^ *in vitro* product (**3**) was purified from scaled reactions by size exclusion chromatography and analyzed by NMR spectroscopy. A combination of 2D experiments confirmed the presence of Kdo and HMBC showed a correlation at δC 101.25/δH 4.20 indicative of an α-2,2-linked Kdo attached to the non-reducing Rib*f* of **2**, matching the authentic *in vivo* linkage (Fig. 4*C*, and Tables S1 and S4).

To confirm the function of ORF7^O4^ *in vivo*, we next tested whether the terminating residue was required for export of the OPS, as seen for serotypes O5, O3 (52), and O12 (33). In all of the terminated OPS examples, a carbohydrate-binding module (CBM) is attached to the C-terminus of the nucleotide-binding domain of the ABC transporter; the CBM binds the terminal epitope and facilitates the export of (only) the terminated OPS. A CBM is predicted by BLAST at the C-terminus of O4 Wzt, consistent with the requirement of the terminal Kdo for binding and export. Previous work in comparable systems has shown that accumulation of non-terminated OPS (that cannot be recognized by the ABC transporter CBM) may result in a fitness cost to bacteria, leading to the selection of mutants that inactivate OPS synthesis (52). To avoid this, the termination-deficient mutant was constructed in *E. coli* CWG286 (53), where a *galE* mutation precludes synthesis of UDP-galactose (preventing synthesis of O4 OPS) unless galactose is added to the growth medium. *E. coli* CWG286 was transformed with plasmid pWQ1147 *rfb*^O4^Δ*orf7*. Galactose addition resulted in the appearance of a high molecular weight polysaccharide reactive with O4 antibodies in western immunoblotting (Fig. 4*A*). Notably, this material was not evident in silver-stained SDS-PAGE like LPS, a property consistent with und-PP-linked polysaccharides (54). When the same experiment was performed in the presence of plasmid-encoded ORF7^O4^, the silver-stained LPS profile was restored, and the chain length of the product was consistent with wildtype OPS. These results indicate that ORF7^O4^ is essential for O4 OPS export, consistent with a role in chain termination and generation of a product recognized by the ABC transporter. With the characterization of ORF7^O4^ and the prediction of a novel fold comes the establishment of a new GT family in the CAZy database – GT137.

### Cloning of the O7 OPS-biosynthesis gene cluster

The O7 OPS contains a tetrasaccharide repeat unit made of three rhamnose (Rha) residues and a Rib*f*, with a terminal methyl-phosphate modification on a non-reducing Rha*p* (Fig. 1*B*) (55, 56). The sequence of the *rfb*^KpO7^ OPS-biosynthesis cluster (MN173773.1) is known, but only the function of the Rib*f* transferase (ORF7^O7^) has been established (42). The *rfb*^KpO7^ cluster contains seven *orf*s that are expected to be involved in O7 biosynthesis (Fig. 1*B*). The *rmlD* and *rmlC* genes encode enzymes involved in dTDP-Rha*p* precursor biosynthesis, and *wzm* and *wzt* encode components of the ABC transporter. The ORF3^O7^ protein is a predicted GT and ORF6^O7^ is a large, multidomain protein with a predicted methyltransferase domain at the N-terminus, a central coiled-coil region predicted by DeepCoil, and two predicted GT modules at the C-terminus. To confirm the overall activity of the cluster, a DNA fragment encompassing *orf3–orf7*^O7^ was cloned into pACYC184 to make plasmid pWQ1151, which was transformed into *E. coli* DH5α. *rmlD* and *rmlC* were not included in the construct because functional copies of *rmlBDAC* genes are present on the *E. coli* DH5α genome and encode enzymes in a pathway for synthesis of dTDP-rhamnose biosynthesis from glucose-1-P (57). The genes for PRPP biosynthesis (for Rib*f*) are also present on the *E. coli* DH5α chromosome (57). Silver-stained SDS-PAGE of whole-cell lysates from transformants containing pWQ1151 showed the presence of high-molecular-weight LPS containing OPS forming a classical ladder pattern (Fig. 1*C*). Western immunoblotting using rabbit antiserum generated against wild-type *K. pneumoniae* O7 cells resulted in robust reactivity to the recombinant OPS, confirming its identity as authentic O7. This was further confirmed by the biochemical data described below.

### ORF3^O7^ is a rhamnosyltransferase required for adapter synthesis

The multidomain organization of ORF6^O7^ suggested it might be involved in polymerization and termination steps. ORF7^O7^ is a validated Rib*f*-transferase (42), leaving ORF3^O7^ as the likely candidate for OPS adapter synthesis. The structure of the adapter region for O7 is unknown. ORF3^O7^ is a predicted GT2 enzyme, a GT family with a diverse range of functions that can be categorized into as many as ten currently identifiable phylogenetically distinct subfamilies (58). GT2 enzymes include characterized rhamnosyltransferases involved in rhamnose adapter synthesis including *K. pneumoniae* O12 WbbL, which adds an α-(1→3)-linked Rha*p* residue to undPP-Glc*p*NAc (59). The O12 enzyme is designated WbbL due to its similarity to the rhamnosyltransferase from the original O16 OPS in *E. coli* K-12 with the same activity (44). Although ORF3^O7^ and WbbL are both GT2 enzymes, they share no significant sequence similarity indicating they likely form distinct subfamilies within the GT2 family. Surprisingly, ORF3^O7^ was found to possess the same activity as WbbL^O12^ based on the ability of ORF3^O7^ to restore OPS biosynthesis in *E. coli* CWG1219 cells transformed with a plasmid (pWQ1153) carrying *rfb*^KpO12^Δ*wbbL* (Fig. S2*B*). This assigned the function of ORF3^O7^ as an und-PP-GlcNAc rhamnosyltransferase.

### ORF6^O7-C^ contains two TDP-Rha-dependent GT modules required for O7 OPS polymerization

**Figure 5:**
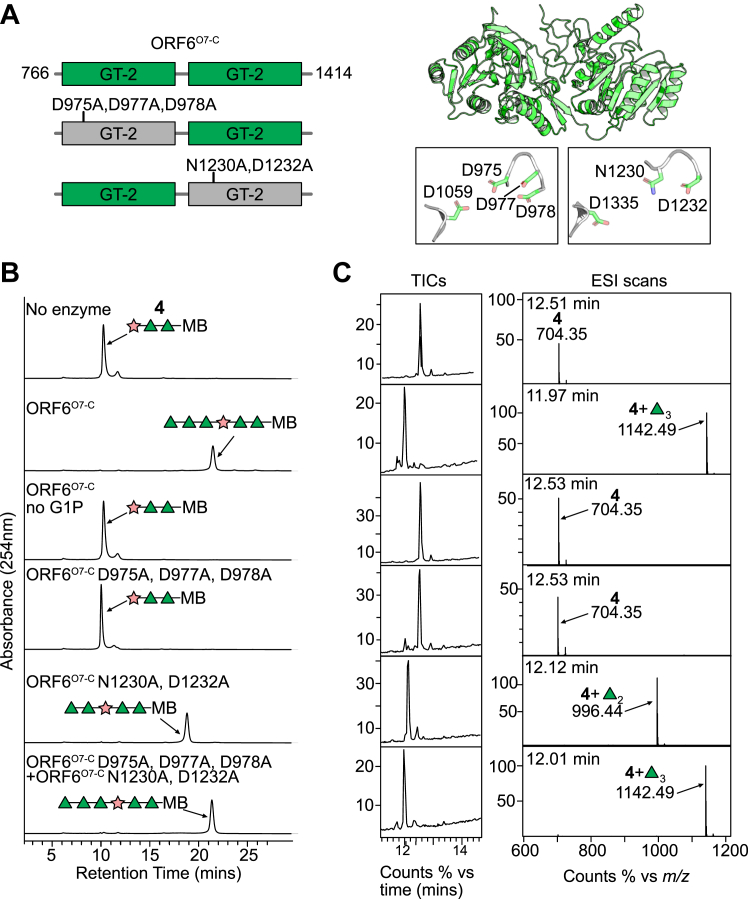
**Biochemical validation of ORF6**^**O7-C**^**, possessing two TDP-Rha*p*-dependent GT modules.***A*, organization of ORF6^O7-C^ (*left*) and the corresponding AlphaFold model *(right*). ORF6^O7-C^ contains two predicted GT2 modules; GT2 enzymes possess a DXD motif and a catalytic Asp in conserved locations. Alanine replacement mutations in the DXD motifs were used to independently inactivate each GT module. *B*, *in vitro* reactions were conducted using repeat mimic **4**. MB represents the octyl-methoxybenzamide aglycone tag of the acceptor. dTDP-Rha*p* substrate was synthesized *in situ* from the precursor, glucose-1-P by the addition of purified RmlABCD enzymes. Reactions were performed in triplicate at 30 °C for 1 h and then stopped by the addition of an equal volume of acetonitrile, prior to analysis by HPLC (*B*) and mass spectrometry (*C*). The ORF6^O7-C^ constructs used in each reaction are identified in the HPLC chromatograms. All mass spectrometry analyses were performed twice on independent reactions.

**Figure 6:**
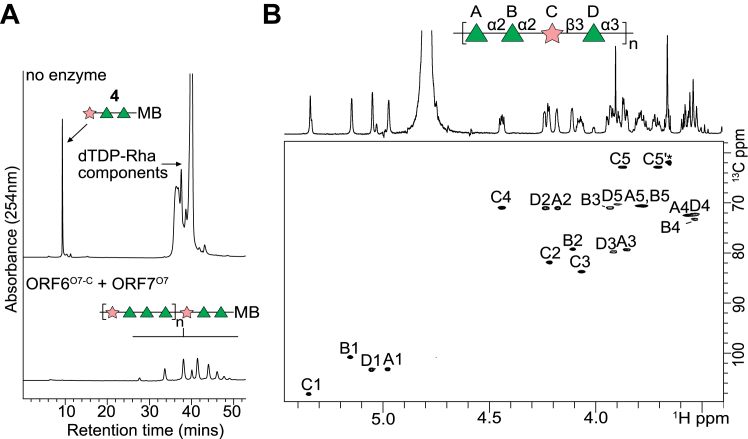
***In vitro* polymerization of the O7 polysaccharide.***A*, *in vitro* reactions containing ORF6^O7-C^ and ORF7^O7^ and acceptor **4** were performed in triplicate with dTDP-Rha*p* (synthesized *in situ*) and PRPP, donors for Rha*p* and Rib*f*, respectively. Polymerization reactions were incubated at 30 °C for 1 h and then stopped by the addition of an equal volume of acetonitrile. The components for dTDP-Rha*p* synthesis completely overlapped with signals of polysaccharide in the NMR spectra and therefore the reaction products were first purified using a C8 SepPak cartridge. The purified polymer appeared as several high molecular weight polysaccharide peaks in HPLC chromatograms. *B*, purified polysaccharide product as analyzed by NMR spectroscopy. The ^1^H,^13^C HSQC spectrum is shown with the assignment and the chemical shifts are reported in Table S5. Chemical shifts of the *in vitro* polysaccharide were identical to those previously reported for the authentic OPS (56). An asterisk marks an unidentified contaminant.

The assignments of ORF3^O7^ and ORF7^O7^ left ORF6^O7^ as the only remaining GT-like protein to contribute to the three Rha*p* residues in the repeat-unit structure. BLAST searches and AlphaFold modeling predicted that ORF6^O7^ contains two C-terminal GT2 domains (Fig. 5*A*). To investigate the activity of ORF6^O7^, a truncated form (ORF6^O7-C^-His_6_) was constructed that contained residues 766 to 1414, spanning the 2 GT modules (Fig. 5*A*). ORF6^O7-C^-His_6_ was incubated in *in vitro* reactions with an acceptor (**4**), representing part of the O7 repeat unit structure (β-D-Rib*f*-(1→3)-α-L-Rha*p*-(1→3)-α-L-Rha*p*) attached to a methoxybenzamide aglycone (Table S1), and dTDP-Rha*p* (generated enzymatically *in situ*). Following incubation, **4** was completely depleted, and a new, later migrating species was observed in the HPLC chromatogram (Fig. 5*B*). MS analysis of the reaction contents revealed a major species (*m/z* 1142.49), consistent with the addition of three deoxyhexose (rhamnose) residues (exact mass = 146 each) to **4** (exact mass = 703) (Fig. 5*C*). As expected, the new product was dependent on TDP-Rha*p* (controlled by the addition of glucose-1-phosphate precursor, which is converted to dTDP-Rha by RmlABCD in the *in vitro* reaction). To confirm that ORF6^O7-C^ was capable of supporting polymerization, a large-scale *in vitro* reaction was performed that included ORF7^O7^ and ORF6^O7-C^, acceptor **4**, and their corresponding PRPP and dTDP-rhamnose donor substrates. The HPLC chromatogram for the reaction contents showed a depletion of **4** and the presence of a range of product species with longer retention times (Fig. 6*A*). This polymeric product was purified by size exclusion chromatography and analyzed by NMR spectroscopy, which showed a structure identical to the authentic O7 repeat unit (56) (Fig. 6*B*, and Table S5).

Next, to assign the individual functions of the two GT2 domains in ORF6^O7-C^, a mutational strategy was employed to inactivate each GT domain. Enzymes belonging to the GT2 family possess a DXD motif that is required for the coordination of a divalent metal cation to stabilize the nucleotide-diphosphate moiety of the donor substrates (60). In GT2 enzymes, the DXD motif is always found in the loop positioned between β-strands 4 and 5 of the Rossmann domain (58). Multiple sequence alignment of the GT2 domains of ORF6^O7-C^ with a biochemically characterized GT2 representative (TarS from teichoic acid synthesis in *Staphylococcus aureus* (61)) identified the corresponding DXD motifs (Fig. S8). Furthermore, the AlphaFold model of ORF6^O7-C^ showed DXD motifs in the location expected for GT2 enzymes (Fig. 5*A* – *right panel*). In the first GT domain, this was a DXDD motif composed of D975, D977, and D978, and in the second GT domain, this was an NXD motif with N1230 and D1232. Plasmids expressing alanine-replacement variants were constructed to change residues in the two DXD motifs (D975A, D977A, D978A, and N1230A, D1232A, respectively), and the proteins were purified and tested for *in vitro* activity (Fig. 5, *B* and *C*). ORF6^O7-C^ D975A, D977A, D978A showed no activity on **4**, whereas ORF6^O7-C^ N1230A, and D1232A completely converted **4** to a later eluting HPLC species. This reaction product eluted earlier than the product obtained with the complete ORF6^O7-C^, suggesting less than three sugars were added to **4**. Indeed, MS showed a major species of *m/z* 996.44, consistent with the addition of two Rha*p* residues (exact mass = 292) to **4**. Reactions including both ORF6^O7-C^ D975A, D977A, D978A and ORF6^O7-C^ N1230A, D1232A with **4**, restored the product with the same retention time as the intact ORF6^O7-C^ product and the mass in MS was consistent with the addition of three Rha*p* residues (exact mass = 438). Taken together with the known structure of the O7 OPS, these results are consistent with the first GT module (located between residues 766–1140) of ORF6^O7-C^ performing the addition of two α-(1→2)-linked Rha*p* residues to Rib*f*. The second GT module (residues 1141–1414) of ORF6^O7-C^ then performs the third (α-(1→3)-linked) Rha*p* addition.

### ORF6^O7-N^ contains a bifunctional methyltransferase-kinase that terminates O7 polymerization

**Figure 7:**
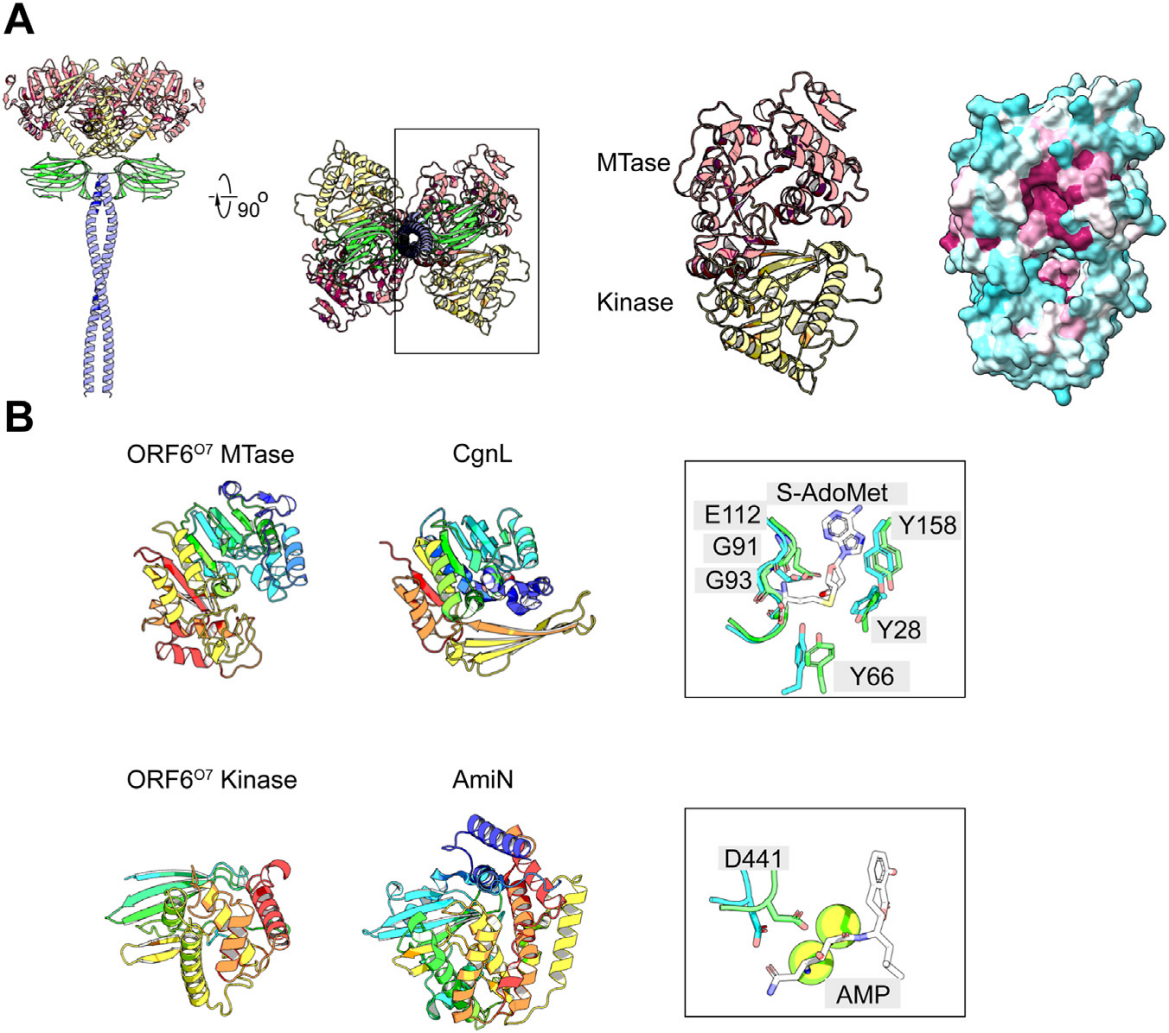
**AlphaFold modeling of the ORF6**^**O7**^**terminator.***A*, ORF6^O7-N^ was modeled as a dimer using AlphaFold, with the methyltransferase in salmon and the kinase in yellow. An additional CBM domain, shown in green, was revealed in the model, but the precise function of this domain was not pursued. ORF6^O7-N^ was modeled as a dimer as coiled-coils form oligomers usually dimers or trimers (86). A dimer was chosen arbitrarily for the simplicity of AlphaFold modeling, but the oligomeric state of ORF6^O7-N^ is unknown. A rotated view of the dimer looking down the axis of the coiled-coil is shown to the *right*, with an expanded view of only the methyltransferase and kinase domains shown next to it. Surface conservation of the isolated methyltransferase and kinase domain is shown, revealing two conserved pockets in each domain (*purple*). *B*, AlphaFold modeling of the ORF6^O7^ methyltransferase and kinase domains compared to the crystal structures of their closest DALI hits with structures shown in rainbow from N- (*red*) to C-terminus (*blue*). The methyltransferase domain shares similarity with the crocagin methyltransferase CgnL (7pd7). An expanded view of the active site is shown on the *right* with CgnL shown in green and ORF6^O7^ in *blue*. The residues shown in sticks correspond to S-Ado-Met binding residues in CgnL. The kinase domain shares similarities with the amicoumacin kinase AmiN (6sul). An expanded view of the active site is shown on the *right* with AmiN shown in *green* and ORF6^O7^ in *blue*. D441 corresponds to the residue selected for mutagenesis in ORF6^O7^; it aligns with the catalytic Asp residue in AmiN. AMP is shown as sticks with the Mg^2+^ ions as *yellow spheres*.

The O7 OPS contains a non-reducing methyl-phosphate moiety linked to a terminal Rha*p*. The precise Rha*p* residue that is modified was not unequivocally established, but NMR data suggested it was the α-(1→3)-linked Rha*p* (56). ORF6^O7^ contains a coiled-coil region that separates the C-terminal GT2 modules from an N-terminal domain with predicted methyltransferase activity. Given the similarity of the glycan terminus to the methyl-phosphate in *K. pneumoniae* O3 group OPS, the absence of a recognizable kinase domain in the N-terminal domain of ORF6^O7^ was therefore surprising. However, an unannotated 223 AA residue segment creates an additional folded domain in the AlphaFold model, located between the predicted methyltransferase domain and the coiled-coil region (Fig. 7*A*). In addition, following the 223 AA domain, an immunoglobulin-like fold, indicative of a CBM, was also predicted. CBMs are used by carbohydrate-active enzymes to recruit substrates for improved enzymatic efficiency, but it is unclear if this domain is essential for function in ORF6^O7^. Currently, CBMs have not been identified in any other terminator enzymes discovered to date.

A multiple sequence alignment was performed using ORF6^O7-N^ orthologs present in candidate polysaccharide biosynthesis clusters identified through tBLASTn (Fig. S9). These included orthologs from alphaproteobacteria, gammaproteobacteria, and cyanobacteria, all containing a predicted N-terminal methyltransferase, an unannotated domain, and a coiled-coil. Some orthologs were connected to additional C-terminal GT modules, while others ended after the coiled-coil. Mapping the sequence conservation to the AlphaFold model showed two well-conserved pockets, with one in the candidate methyltransferase domain and the other in the unannotated domain (Fig. 7*A*).

A DALI search against the PDB with the modeled structure of the predicted methyltransferase domain of ORF6^O7^ shows the closest related structural ortholog to be the *Chondromyces crocatus* Crocagin N-methyltransferase CgnL, sharing 16% sequence identity, a Z-score of 16.7 and RMSD of 2.9 Å (Fig. 7*B*). Methyltransferases employ a conserved glycine-rich loop (E/DXGXGXG motif) to bind the donor, S-adenosyl-L-methionine (S-Ado-Met). A three-dimensional superposition of the CgnL structure with the relevant domain from ORF6^07^ showed G91 and G93 in ORF6^O7^, as well as a number of other highly conserved residues (Y28, Y66, E112, and Y158) occupied similar positions to the corresponding residues used in CgnL to bind S-Ado-Met (62). A DALI search of the PDB with the predicted structure of the unannotated domain from ORF6^O7^ identified the closest related structural ortholog to be the amicoumacin aminoglycoside kinase (AmiN) from *Bacillus pumilus* (Fig. 7*B*). The similarity was low with 11% sequence identity, a Z-score of 7.4, and an RMSD value of 3.4 Å, but the three-dimensional alignment of the proteins showed the catalytic Asp residue (D202) in AmiN (63) was located in a similar position to D441 in ORF6^O7^ (Fig. 7*B*). We therefore hypothesized this unannotated domain to be responsible for the kinase activity.

**Figure 8:**
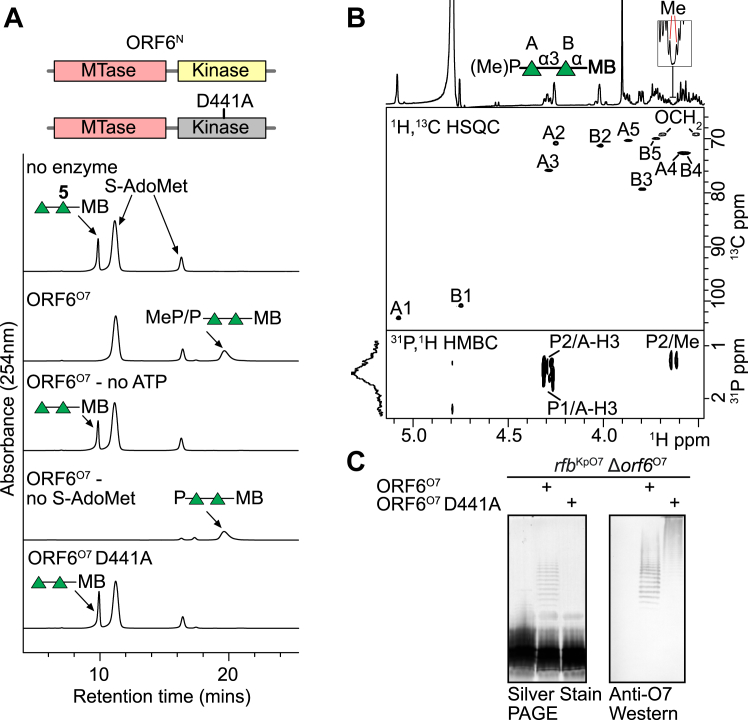
**Characterization of a methyltransferase–kinase terminator in ORF6**^**O7**^**.***A*, organization of the enzymatic modules in ORF6^O7-N^ and the kinase mutant used in the analysis. *In vitro* reactions were performed with repeat unit mimic **5**, ORF6^O7^ (or mutant variants of this enzyme) and ATP and S-Ado-Met as donor substrates. Reactions were performed in triplicate at room temperature overnight, and then stopped by the addition of an equal volume of acetonitrile and analyzed by HPLC. Notably, two peaks were present in all samples and were attributed to S-Ado-Met. These substrate peaks did not impact the interpretation of the results. *B*, *in vitro* reactions with wildtype ORF6^O7^ were scaled up and the reaction product was purified and analyzed by NMR spectroscopy. The ^1^H,^13^C HSQC spectrum is shown in the *upper* panel with the labelled assignments (chemical shifts are reported in Table S6). An expanded region is shown to highlight the methyl peak. The *lower* spectrum shows the ^31^P,^1^H HMBC spectrum, which establishes the linkages to the phosphate in the terminating moiety. The ^31^P spectrum showed two phosphorus species (P1 and P2) with slightly different chemical shifts. P1 showed a correlation to A-H3 only, while P2 showed correlations to both A-H3 and methyl. These results reflect a mixed product with only partial methylation. *C*, The ORF6^O7-N^ variants were tested for activity *in vivo* by examining the LPS profile of *E. coli* DH5α containing *rfb*^KpO7^Δ*orf6* alone, or together with the complementing plasmid expressing ORF6^O7^. Whole-cell lysates were analyzed by SDS-PAGE and western immunoblotting with rabbit O7 antiserum. The absence in some samples of silver staining material and accumulation of high molecular weight material detected by the immunoblots is a phenotype expected for an inability to export OPS for ligation. The PAGE and western immunoblots were performed in triplicate.

To confirm the function of ORF6^O7-N^ as the O7 terminator, *in vitro* reactions were performed with full-length ORF6^O7^, a repeat unit mimic (**5**, Table S1) as the acceptor and the ATP and S-Ado-Met donors. Previous studies on *E. coli* WbdD (identical to WbdD from *K. pneumoniae* O3) showed that part of the coiled-coil region was important for the proper folding and enzymatic function of the adjacent kinase (25). To ensure the O7 terminator was folded, full-length ORF6^O7^ was used in these reactions. HPLC analysis showed the complete conversion of **5** to a new species with a later retention time (Fig. 8*A*). MS analysis showed the reaction products were a mixture of phosphorylated **5** (*m/z* = 652.27) and methylated-phosphorylated **5** (*m/z* = 666.28) (Fig. S10). Although all of **5** was phosphorylated, conversion of the phosphorylated species to the methylated end-product was incomplete under the conditions tested, but this did not compromise the assignment of the overall bifunctional activity of ORF6^N^. Given the lack of sequence similarity of the kinase domain of ORF6^O7-N^ to other known kinases, we wanted to confirm its activity through a mutational strategy. Reactions containing ORF6^O7^ D441A, **5**, and ATP showed no conversion of **5** to a new product (Fig. 8*A*), consistent with the inactivation of the kinase. MS also confirmed no modification of **5** (Fig. S10).

Next, to confirm the linkage between the terminal Rha*p* and the methyl-phosphate moiety, a large-scale reaction was performed, and the product (**6**) was purified by size exclusion chromatography. NMR spectroscopic analysis of **6** confirmed the prior mass spectrometry results from analytical reactions, with **6** containing a mixture of phosphorylated and methylated-phosphorylated **5** (Fig. 8*B*, and Tables S1 and S6). The site of the modification was determined to be at position 3 of the non-reducing α-(1→3)-linked Rha*p* (residue A), evident by the large downfield shift of A-H3/C3 of **6** compared to that of **5**. This location is consistent with the proposed location in the natural product. ^31^P NMR spectroscopy showed two phosphorylated species. One phosphorus showed a correlation at δP 1.4/δH 4.31,4.27 in the ^31^P,^1^H HMBC, corresponding to A-H3, while the second phosphorus showed the same correlation to A-H3 and an additional correlation at δP 1.2/δH 3.65,3.61, indicative of a methyl group. The methyl signal was sufficiently small that it was not detectable in the HSQC spectrum and only a small signal was visible in the one-dimensional ^1^H spectrum, but its presence in the ^31^P,^1^H HMBC spectrum confirms its addition to phosphate.

Finally, to demonstrate the terminator function *in vivo*, pWQ1152 was generated with the O7 cluster containing a deletion in *orf6*^O7^ and used to transform *E. coli* DH5α. The deletion of *orf6*^O7^ resulted in no O7 backbone production because ORF6^O7^ also contains the rhamnosyltransferase component of the polymerase (Fig. 8*C*). Complementation studies were then performed by introducing plasmids expressing wild-type ORF6^O7^ or ORF6^O7^ D441A. Expression of wildtype ORF6^O7^ restored lipid A-core linked OPS production, evident by the silver-stained material in SDS-PAGE and the LPS species reacting with O7 antibodies in the corresponding western immunoblot (Fig. 8*C*). ORF6^O7^ D441A could not restore detectable silver-stained OPS, but high-molecular-weight polysaccharide was detected upon western blotting as expected for the production of intracellular (non-terminated/exported) OPS (Fig. 8*C*). The *in vivo* complementation data is therefore consistent with the *in vitro* data in the context of the requirement for termination for the ability of the export machinery to recognize only terminated glycans.

### Protein–protein interactions involving the components of O4 and O7 OPS polymerization machinery

**Figure 9:**
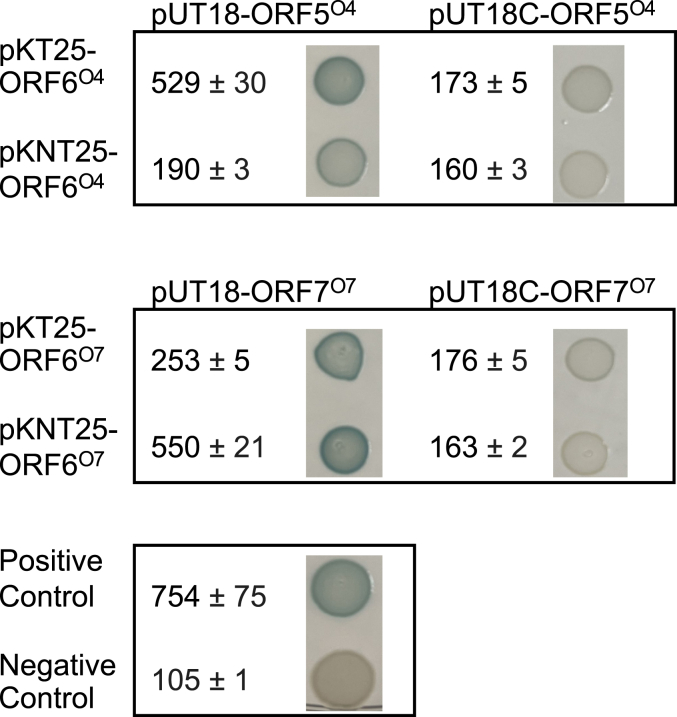
**Colorimetric detection and β-galactosidase activity values from bacterial two-hybrid analyses.** Data represents triplicate samples with standard deviation shown. The positive control was pKT25-Zip with pUT18C-Zip and the negative control was the pUT18 with pKT25 empty vectors.

The O4 and O7 OPS-biosynthesis systems both contain Rib*f* residues added to the repeat unit by dedicated ribofuranosyltransferase proteins. O4 and O7 OPS polymerization therefore requires a combination of the ribofuranosyltransferase with other enzymes; ORF6^O4^ and ORF6^O7-C^, respectively. Because other serotypes place all polymerase GT modules in a single polypeptide, we speculated that efficient polymerization of O4 and O7 OPSs may depend on close interactions between the collaborating proteins in protein complexes. This hypothesis was tested in bacterial two-hybrid experiments. A panel of T18 and T25 fusions (containing fragments of adenylate cyclase; (64)) were made and different combinations were tested for reconstituted adenylate cyclase activity measured as β-galactosidase induction. For each OPS-biosynthesis system, one combination was found with robust β-galactosidase activity, indicating that the polymerase enzymes in both systems were in close proximity (Fig. 9). The inability to obtain activity from all combinations (with C- and N-terminal fusions) was not resolved but bacterial two-hybrid chimeric fusions are potentially sensitive to steric hindrance depending on the location of the reporter fragment. To confirm that the interacting enzyme variants remained in an active conformation, their polymerization capability was validated *in vitro* using cell-free lysates prepared from *E. coli* BTH101 transformed with plasmids encoding interacting combinations (Fig. S11).

## Discussion

We describe here the initiation, polymerization, and termination reactions in pathways for biosynthesis of the *K. pneumoniae* O4 and O7 OPSs (shown in the schematic of Fig. 1*B*). When added to the existing information on *K. pneumoniae* OPS assembly in other serotypes, this offers an unprecedented level of breadth and depth of experimentally-validated biochemical insight for a class of structurally diverse glycans in a given species.

The glycan chain-termination strategy described here is widespread across the bacterial kingdom, where it participates in the biosynthesis of different classes of polysaccharides that are assembled by ABC transporter-mediated processes (23). In the majority of *K. pneumoniae* OPS (serotype O1 being an exception; (40)), the terminating moiety controls chain-length. These examples all possess a protein with an extended coiled-coil motif, which is a characteristic marker for the assembly strategy, and has been established to be a molecular ruler for glycan chain length in the *E. coli* O9 serogroup (identical to *K. pneumoniae* O3) (28) and in *K. pneumoniae* O12 (29). The data presented here for *K. pneumoniae* serotypes O4 and O7 significantly extends our understanding of how the coiled-coil proteins are integrated into functional polymerization–termination machinery. In all cases, the terminating enzyme is located at the distal end of the coiled-coil, providing separation from polymerizing GT modules, and facilitating the molecular ruler function. However, it is now apparent that the distribution of enzyme modules required for polymerization is more flexible.

The simplest system, in serotype O12, puts all of the required enzymes in a single polypeptide with the 2 GT modules forming the polymerase located near the C-terminus (29). Serotype O7 is the closest format to this, with two GT2 modules of the polymerase located at the C-terminus of the coiled-coil protein, and one GT module (the ribofuranosyltransferase, ORF7^O7^) provided by a standalone protein. In contrast, in *E. coli* O9/*K. pneumoniae* O3, only the terminating methyltransferase-kinase is present in the coiled-coil protein WbdD (26, 28) and the WbdA polymerase is a dual GT module enzyme that is recruited by a region of WbdD proximal to the membrane, to form a complex (26, 65). The separation of polymerization and termination enzymes is carried further with serotype O4, where each activity resides in a separate polypeptide.

It is unclear what dictates the different distributions of polymerase modules. Serotypes O4 and O7 both involve ribofuranosyltransferases that possess two catalytic domains; a PRPP-dependent gPRT transferase Rib*f*-5-P and a PRP that dephosphorylates the product to facilitate chain extension (42). However, there is nothing inherent in the structure of the ribofuranosyltransferase that precludes its incorporation into a multifunctional protein. For example, the capsule polymerase from *Haemophilus influenzae* serotype b contains an orthologous ribofuranoslytransferase, together with a ribitol phosphate transferase, in a single polypeptide (42, 66). Notably this capsule assembly pathway also involves an ABC transporter but there is no chain terminating residue (67). It seems more likely that the organizational differences reflect complex evolutionary histories where genes encoding catalytic modules have been incorporated from different sources to generate the current loci. Regardless of the distribution of catalytic elements, the demonstrated protein–protein interactions enable the formation of a functional complex and the coiled-coil protein provides a membrane-associated scaffold for complex assembly. While we have not experimentally assessed the presence of adapter GTs in these complexes, previous studies with *K. pneumoniae* O2a revealed interactions between the adapter GTs (WbbN and WbbO) and the polymerase WbbM (41).

Given the propensity of bacteria to exchange genetic information, it is not surprising to find that similar genetic loci are found in other bacteria (Fig. S12). For example, a gene cluster identical to *rfb*^KpO4^ is present in *E. coli* O20ab and, as expected, the OPS in these bacteria has the same structure. Several gene clusters resembling *rfb*^KpO7^ occur in species that are more distant. The OPS of *Franconibacter pulveris* O1 contains a backbone repeat unit similar to *K. pneumoniae* O7 (68) but lacking one of the rhamnose residues and is modified by a side chain composed of two 4-deoxy-D-arabinohexose residues. The mode of synthesis of the side chain residues is unclear because the sequence of the genetic locus is not available. The exopolysaccharide in *Gluconacetobacter diazotrophicus* PAL5 has the same main chain as *K. pneumoniae* O7, but includes a glucose side chain (69). In this case, the gene cluster encodes homologs of ORF6^O7^ and ORF7^O7^. Finally, an unknown surface polysaccharide from *Methanoculleus marisnigri* JR1 also has the same backbone as O7 (70), and genes encoding homologs of ORF6^O7^ and ORF7^O7^ homologs were identified. The similarities offer facile preliminary allocation of biosynthesis functions, and although general assignments are possible, some caution is still required in making definitive assignments from sequence predictions in the absence of a solved polysaccharide structure.

The serotype O4 and O7 systems provide examples where a carbohydrate structural element seen in other bacteria, sometimes within the same species, is synthesized by enzymes sharing remarkably low similarity. WbbL enzymes in a range of bacteria catalyze the transfer of Rha*p* from TDP-Rha*p* to a Glc*p*NAc acceptor (in an α-(1→3) linkage, where the glycan structures have been determined). The WbbL enzyme from O12 and ORF3^O7^ are functionally interchangeable (Fig. 1, *A* and *B*) but share only low amounts of sequence similarity (12% sequence identity). AlphaFold models of the two share a similar Rossmann core (Z-score of 15.4, RMSD of 3.6 Å) but aside from the central domain, significant divergence is present in the C-terminal helical portion of the proteins (Fig. S13). The ORF6^O7^ methyltransferase–kinase is distinct from the WbdD enzyme that installs a terminal methylphosphate onto the *K. pneumoniae* O3/*E. coli* O9 glycan chain. While the ORF6^O7^ methyltransferase domain shares some structural resemblance to WbdD, the kinase domain is quite different in the two serotypes from both sequence and structural perspectives.

While the majority of the characterized GTs involved in bacterial glycan synthesis typically transfer a single sugar, ORF6^O7^ contains one GT2 domain capable of adding two Rha*p* residues to a Rib*f* acceptor. The phenomenon is rare and only a few examples have been described in detail. One is the *E. coli* O9 (*Klebsiella* O3) WbdA polymerase that has 2 GT domains, one transfers two α-(1→3)-Man*p* residues, while the other adds two (serotype O9a) or three (serotype O9) α-(1→2)-Man*p* residues (24, 26). Both activities recognize Man*p* acceptors. A small number of sequence changes in WbdA result in the different repeat unit structures but the underlying mechanistic explanation is lacking (71). In the biosynthesis of *N*-linked glycans in *Campylobacter jejuni*, PglH transfers exactly three GalNAc residues from the UDP-linked donor to a GalNAc acceptor (72). PglH possesses an α-helix that supports a glycan-counting function (73), but similar structural elements are not apparent in predicted structures of WbdA or ORF6^O7^. Additional complexity in ORF6^O7^ is the need to recognize different acceptor sugars (Rib*f* and Rha*p*) and examples of this phenomenon are few. WaaA is a CMP-Kdo-dependent GT involved in the biosynthesis of the lipid A-core in many Gram-negative bacteria. In *E. coli*, this enzyme transfers two Kdo onto a GlcN of the lipid IVA biosynthetic intermediate (47). WaaA from *Chlamydia trachomatis* adds four Kdo residues in a similar reaction (74). The mycobacterial cell wall initiating enzyme, GlfT1, adds two galactofuranose (Gal*f*) residues in the early stages of arabinogalactan biosynthesis by producing first Gal*f*-(1→4)-Rha*p* and then Gal*f*-(1→5)-Gal*f* linkages (75). GlfT1, like ORF6^O7^, transfers a monosaccharide to different acceptors in different cyclic ring forms: Gal*f* and Rha*p*. The mechanism(s) by which these enzymes achieve this is intriguing and currently not resolved; thus, structural biology initiatives on these proteins, and other examples, are warranted.

The assembly pathways for *K. pneumoniae* OPSs have provided several new GT families. The WbbB protein in serotype O12 possesses 3 GT modules, each an anchoring representative for a new family in CAZy (GT99, GT102, GT103). ORF7^O4^ also brings with it a new GT family (GT137) and AlphaFold modeling of ORF7^O4^ reveals topological changes that distinguish it from the canonical GT-B fold. The majority of characterized Leloir GTs (*i.e.*, those using NMP/NDP-sugar donors) are categorized as either GT-A or GT-B folds (60). Additional folds have been identified more recently including GT-D (76), GT-E (77), and the ribosyltransferase fold (42). ORF7^O4^ represents a new candidate fold, but a solved structure is now required to confirm the AlphaFold prediction presented here and to elucidate the active site details for substrate binding.

The importance of a detailed understanding of a set of biosynthetic systems, as now available for *K. pneumoniae*, lies in the ability to identify broader concepts in the enzymology and architecture of assembly complexes. They provide influential prototypes to interpret related systems in other bacteria, establish fundamental guiding principles for glycan assembly in bacteria, and provide new biocatalysts that might be employed to build neoglycoconjugates. In addition, the development of glycoengineering strategies to produce glycans for biomedical and other applications in heterologous hosts is greatly facilitated by understanding the classes of proteins and functions required to generate the authentic glycan product. Glycoengineering is currently being employed to pursue immunotherapeutic approaches exploiting capsular polysaccharides (78) and OPSs (18, 79) from *K. pneumoniae*. The work described here will facilitate the application of such strategies to a broader range of *K. pneumoniae* serotypes.

## Experimental procedures

### Bacterial strains and culture conditions

Bacterial strains used in this study are listed in Table S7 and were grown at 37 °C in lysogeny broth (LB) medium unless otherwise specified. 50 μg/ml kanamycin, 100 μg/ml ampicillin or 34 μg/ml chloramphenicol, were added where appropriate. For the *orf7*^O4^ deletion mutant, glucose was used to repress O-antigen production in the *E. coli* CWG286 *galE* host background (53). Subcultures were made in the presence of 0.4% glucose and growth was continued until mid-log phase, after which the cells were washed and transferred to LB containing 0.2% galactose to induce OPS production. Induction was conducted for 3 h. For complementation of the *orf6*^O7^ deletion, 0.4% glucose was added to repress gene expression from the pBAD24-based complementation plasmids until the mid-log phase. The cells were then washed and transferred to LB containing 0.2% arabinose to induce expression of plasmid-encoded genes for 3 h.

### DNA methods

Genomic DNA was isolated using the PureLink Genomic DNA mini kits from ThermoFisher (Invitrogen). PCR products and plasmids were isolated using GeneJET PCR, or plasmid purification kits (ThermoScientific), respectively. All cloning was performed using KOD Hot Start DNA polymerase (Novagen), with oligonucleotide sequences whose sequences are provided in Table S8. Plasmid constructs were confirmed either with Sanger sequencing from the Advanced Analysis Centre at the University of Guelph or by whole-plasmid sequencing from Plasmidsaurus (https://www.plasmidsaurus.com).

### Bacterial two-hybrid experiments to assess protein-protein interactions

Overnight cultures of *E. coli* BTH101 expressing variants of paired two-hybrid constructs were diluted 1:100 into LB containing kanamycin, ampicillin and 0.5 mM 1-thio-β-ᴅ-galactopyranoside (IPTG) and grown at 30 °C to an A_660nm_ of ∼0.5. β-galactosidase activity was assessed using the microplate non-stopped protocol for the yeast β-galactosidase assay kit (Thermo Fisher Scientific). The yeast protein extraction reagent (Y-PER) was replaced with bacterial protein extraction reagent (B-PER). Absorbance was read at 420 nm using a SynergyH1 microplate reader (BioTek). Three replicates were performed for each combination, starting from independent cultures. Agar plate images were made by growing subcultures in the presence of 0.5 mM IPTG until OD_600_ of ∼0.6 and spot plating 5 μl of culture onto plates containing 0.5 mM 1-thio-β-ᴅ-galactopyranoside (IPTG), 40 μg/ml of 5-bromo-4-chloro indolyl galactopyranoside, ampicillin and kanamycin and subsequently growing for 24 h at 30 °C.

### Bioinformatic analyses

Initial protein functional predictions were performed using BLAST (https://blast.ncbi.nlm.nih.gov/Blast.cgi) and the Conserved Domain Database (https://www.ncbi.nlm.nih.gov/Structure/cdd/wrpsb.cgi). AlphaFold models were obtained from Uniprot when available, or constructed manually using ColabFold (80). AlphaFold models were assessed on an individual basis; all contained pLDTT values greater than 80 over the majority of the structures classifying the models as confident. Multiple sequence alignments were performed with ClustalW (81) and visualization of the alignments was conducted in ESPript (82). Three-dimensional structural alignments were performed using DALI (83) and all protein structures were visualized in Pymol v.2.1.

### SDS-PAGE and immunoblotting

LPS samples were prepared as proteinase K-treated whole-cell lysates as described previously (84). Cell density was assessed by OD_600nm_ and an equal amount of cells (equivalent to 1 ml with an OD_600nm_ = 1) were collected, resuspended in SDS-PAGE loading buffer, and boiled for 10 min. The samples were then treated with proteinase K at 55 °C for 1 h. Samples were analyzed on 12% tris-glycine gels and visualized using silver staining, or transferred to nitrocellulose membranes (Protran, GE Healthcare) for western immunoblotting. Transfer for LPS immunoblots was conducted at a constant current of 200 mA for 45 min in a buffer containing 25 mM Tris, 150 mM glycine, and 20% (v/v) methanol. Protein immunoblots were performed using the Pierce Power system and the supplied buffer. After transfer, membranes were blocked in 5% skim milk (w/v) dissolved in TBST (10 mM Tris-Cl, pH 7.5, 150 mM NaCl, 0.005% (v/v) Tween 20). Rabbit anti-O4 and O7 antisera were used at a 1:500 dilution made in skim milk-TBST. Anti-His_5_ antibody (Cedarlane) was used at 1:1000 made in BSA-TBST. Goat anti-rabbit-conjugated alkaline phosphatase (1:3000) (Cedarlane), or goat anti-mouse-conjugated alkaline phosphatase (1:3000) (Jackson laboratories), were used as secondary antibodies. Blots were developed with nitroblue tetrazolium and 5-bromo-4-chloro-3-indolyl phosphate (Roche Applied Science).

### Antiserum production

All animal handling and immunization was performed by staff in the Central Animal Facility at the University of Guelph, following an approved protocol. Rabbit anti-O4 and anti-O7 were produced in New Zealand white rabbits using formalin killed cells of capsular-deficient spontaneous mutants of *K. pneumoniae* 1702 and *K. pneumoniae* 264-1, respectively. Killed cells were resuspended in 0.85% (w/v) NaCl at a density equivalent to ∼10^8^ cfu/ml and mixed 1:1 with Freund’s incomplete adjuvant (Sigma). Immunizations were administered every 2 weeks for a total of 6 weeks. After 6 weeks, blood was collected. The serum was separated and stored at −80 °C for long-term storage and −20 °C for short term. Antibody specificity was established by western immunoblots of lysates from different serotypes (Fig. S14) and confirmed by data in the main text.

### Expression and purification of proteins

Gene expression from pET-vector and pBAD-vector plasmids was induced by adding 0.5 mM IPTG or 0.2% ʟ-arabinose, respectively. Cultures were transferred to 18 °C overnight for expression. Cells from overnight cultures were collected by centrifugation and resuspended in buffer A (50 mM Tris pH 7.5, 500 mM NaCl) with 10 mM imidazole. Cells were disrupted with an Avestin C3 Emulsiflex. Lysates were cleared by centrifugation at 12,000*g* for 20 min. Membranes were then separated from soluble proteins by centrifugation at 100,000*g* for 1 h and resuspended in buffer A. For purification of His_6_-tagged proteins, lysate was applied to Ni-NTA agarose with a 2 ml bed volume. Resin was washed with 10 bed volumes of buffer A containing 10 mM imidazole, followed by a wash with buffer A containing 30 mM imidazole. Protein was eluted with buffer A containing 250 mM imidazole. Eluates were concentrated using spin concentrators (Sartorious), and buffer was exchanged into buffer A using PD-10 Sephadex G-25 columns (Cytiva). Protein concentrations were estimated using A_280nm_ (measured using a NanoDrop 2000) with theoretical extinction coefficients obtained from ProtParam. Total protein concentration of membranes was measured using the DC protein assay (BioRad).

### *In vitro* GT reactions

Standard reactions were performed in 20 μl volumes of 50 mM Tris containing 50 mM MgCl_2_, 5 mM of appropriate donor(s) (PRPP, UDP-Gal*p*, ATP and S-Ado-Met). Acceptors **1** and **2** were used at 1 mM and acceptors **3** and **4** were used at 0.5 mM. dTDP-Rha*p* was generated *in situ* using purified RmlABCD (85) (10 μg of each protein) with 5 mM glucose-1-phosphate, 5 mM thymidine triphosphate (dTTP) 0.25 mM NAD^+^ and 5 mM NADH. CMP-Kdo was generated *in situ* using purified KdsB (20 μg) with 2 mM Kdo and 2 mM CTP as described previously (50). Standard reaction mixtures contained 20 μg of soluble enzymes, or 20 μg of total membrane protein for ORF7^O4^ reactions. Reactions involving ORF6^C^ were performed at 30 °C for 1 h, while reactions for ORF6^O7^ and ORF7^O4^ were performed at room temperature overnight.

To generate polysaccharide products for NMR, large-scale reactions were prepared by scaling the standard reactions by 50×. Terminator reactions were scaled by 175×. Enzymatic synthesis of **2** was accomplished using a 175× scaled-up reaction of **1** with ORF6^O4^ and 2.5 mM UDP-Gal*p* as the donor substrate. After incubation, protein was precipitated and the reaction products were purified by SepPak. Next, the purified ORF6^O4^ reaction product was incubated with ORF5^O4^ and 2.5 mM PRPP to install a Rib*f* residue, in a 175× reaction. The final product (**2**) was purified by SepPak, dried down, and resuspended in water for use as an acceptor in reactions with ORF7^O4^.

Reactions conducted to assess the function of the chimeric proteins for bacterial two-hybrid protein interaction experiments were performed using whole-cell lysates. Briefly, cells from 250 ml cultures of *E. coli* BTH101 expressing either ORF5^O4^-T18 and T25-ORF6^O4^ or ORF6^O7^-T25 and ORF7^O7^-T18 (or the control transformed with empty vector) were collected by centrifugation and lysed by sonication. The lysate was cleared and concentrated to 5 ml using a Sartorius 10,000 MWCO spin concentrator. *In vitro* reactions were then performed as above using 7.4 μl of concentrated lysate and the appropriate donors/acceptors for polymerization.

### HPLC analysis

Reaction contents were analyzed by HPLC using an Agilent 1260 Infinity II LC system. A 10 μl aliquot of the reaction mixture (mixed 50:50 with acetonitrile) was injected onto a GLYCOSEP N column (4.6 × 250 mm, Prozyme) and eluted with a gradient of ammonium formate buffered water:acetonitrile. Buffer A contained 10 mM ammonium formate (pH 4.4) in 80% acetonitrile. Buffer B contained 30 mM ammonium formate (pH 4.4) in 40% acetonitrile. Separation was accomplished with a linear gradient of 100% A to 100% B over 160 min (0.4 ml/min), followed by a 2 min gradient of 100% B to 100% C.; returning to 100% A over 2 min and holding for 15 min (1 ml/min); followed by 0.4 ml/min for 5 min in A. The column temperature was 30 °C and elution was monitored at 254 nm. Prior to HPLC analysis, all ORF7^O4^ products (and any remaining acceptor) were first bound to a SepPak C8, eluted in 50% methanol, and dried down. This was done to separate the reaction product from CMP-Kdo biosynthesis substrates, whose peaks overlapped with those of the acceptor. The dried reactions were resuspended in 20 μl and analyzed by HPLC.

### Mass spectrometry

Liquid chromatography–mass spectrometry analysis was performed on Waters Acquity I-class HPLC coupled to a Synapt G2Si mass spectrometer in the Mass Spectrometry Facility of the Advanced Analysis Centre, University of Guelph. A C18 column (Agilent Poroshell 120, 50 mm × 4.6 mm 2.7 μm) was used for chromatographic separation with the following solvents: water with 0.1% formic acid (A) and acetonitrile with 0.1 formic acid (B). The mobile phase gradient was as follows: initial conditions were 2% B hold for 1 min then increasing to 100% B in 18 min followed by column wash at 100% B for 1 min and 10 min re-equilibration. The flow rate was maintained at 0.4 ml/min. The mass spectrometer electrospray capillary voltage was maintained at 3.0 kV, the source temperature was 80 °C, cone gas was supplied at 50 L/hr, the desolvation temperature was 600 °C and desolvation gas flow was 1000 l/hr with a nebulizing gas flow of 7 bar. The mass-to-charge ratio was scanned across the *m/z* range of 300 to 2000 *m/z* in MS resolution mode using FastDDA (Waters). The instrument was externally calibrated with sodium iodide. The sample injection volume was 1 μl. Data analysis was performed using the MassLynx Version 4.2 software (Waters).

### Nuclear magnetic resonance spectroscopy

NMR spectroscopy of enzyme reaction products was performed in the NMR facility of the Advanced Analysis Centre, University of Guelph. Experiments were performed on a Bruker Avance III 600 MHz spectrometer, equipped with a cryoprobe. Samples were deuterium exchanged (twice) by resuspending in 99.0% D_2_O and lyophilizing and analyzed in 99.96% D_2_O. 3-trimethylsilylpropanoate-2,2,3,3-d_4_ (δ_H_ 0 ppm, δ_C_ −1.6 ppm) provided an internal standard. 80 ms mixing times were used for the TOCSY experiments. ^31^P-based NMR experiments were performed on a Bruker 400-MHz Avance III spectrometer equipped with a 5-mm broadband Prodigy cryoprobe.

### Acceptor synthesis

The synthesis of acceptor **1** and acceptor **4** is described in the Supporting information. Disaccharide **5** was synthesized as reported previously (42).

## Data availability

All mass spectrometry, NMR data and raw data are available upon request.

## Supporting information

This article contains supporting information (24, 39, 41, 42, 53, 54, 64).

## Conflict of interest

The authors declare that they have no known competing financial interests or personal relationships that could have appeared to influence the work reported in this paper.
