# Supplementary material for: Klebsiella pneumoniae O-polysaccharide biosynthesis highlights the diverse organization of catalytic modules in ABC transporter-dependent glycan assembly
Source: J Biol Chem. 2024 May 28;300(7):107420. doi: 10.1016/j.jbc.2024.107420 (PMC11231755; doi:10.1016/j.jbc.2024.107420)
Supplement: Supporting information [file mmc1.pdf]

SUPPLEMENTARY INFORMATION

Klebsiella pneumoniae O-polysaccharide biosynthesis highlights diverse organization of catalytic modules in ABC transporter-dependent glycan assembly

Steven D. Kelly[†1], Danielle M. Williams[†1], Shawna Zhu[1], Taeok Kim[2], Manas Jana[2], Jeremy Nothof[2], V. Narasimharao Thota[2], Todd L. Lowary[*2,3,4] and Chris Whitfield[*1]

[1]Department of Molecular and Cellular Biology, University of Guelph, Guelph, Ontario N1G 2W1, Canada
[2]Department of Chemistry, University of Alberta, Edmonton, Alberta, Canada T6G 2G2
[3]Institute of Biological Chemistry, Academia Sinica, Academia Road, Section 2, #128, Nangang, Taipei, 11529, Taiwan
[4]Institute of Biochemical Sciences, National Taiwan University, Section 4, #1, Roosevelt Road., Taipei 10617, Taiwan

*Corresponding authors
†Authors contributed equally
Todd L. Lowary. Email: toddlowary.sinica@gmail.com
Chris Whitfield. Email: cwhitfie@uoguelph.ca.

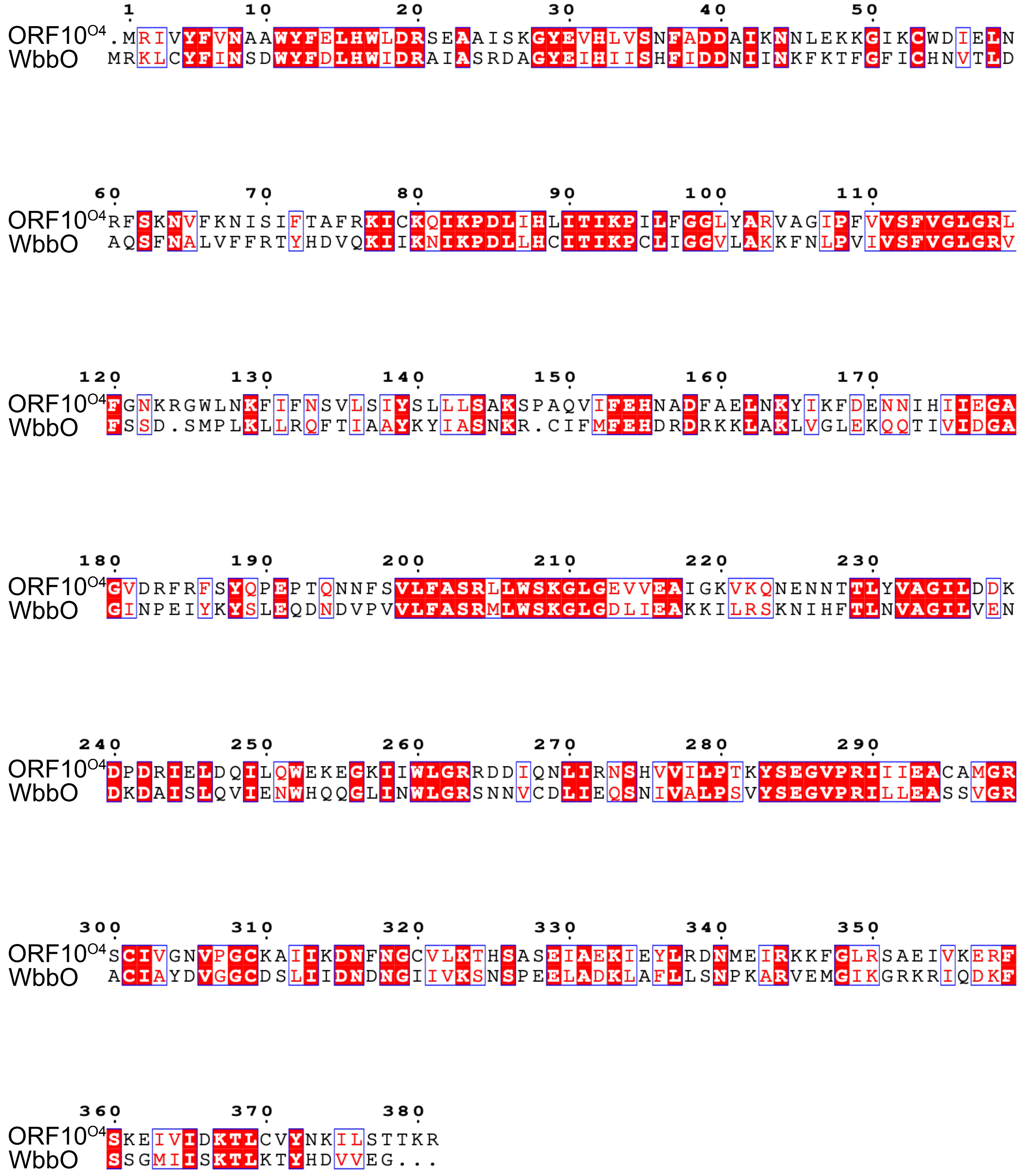

SI Figure 1. Alignment of *K. pneumoniae* O2a WbbO (AAC98410.1) with *K. pneumoniae* O4 ORF10 (ALX35081.1).

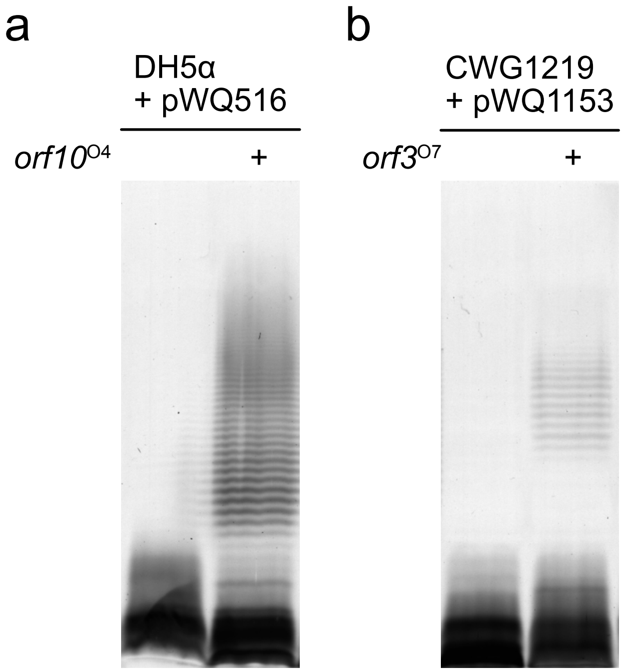

SI Figure 2. SDS-PAGE silver stain of mutant complementation using genes encoding adapter GTs from O4 and O7. **a,** pWQ516 contains the *K. pneumoniae* O2a cluster with a deletion in *wbbO*. Coexpression of pWQ516 with *orf10*^{O4} restored O2a biosynthesis, confirming the function of ORF10^{O4} as an UDP-Gal*p*-dependent GT acting on undPP-GlcNAc. **b,** pWQ1153 contains the *K. pneumoniae* O12 cluster with a deletion in *wbbL*. Coexpression of pWQ1153 with *orf3*^{O7} restored O12 biosynthesis confirming the function of ORF3^{O7} as a dTDP-Rha-dependent GT acting on undPP-GlcNAc.

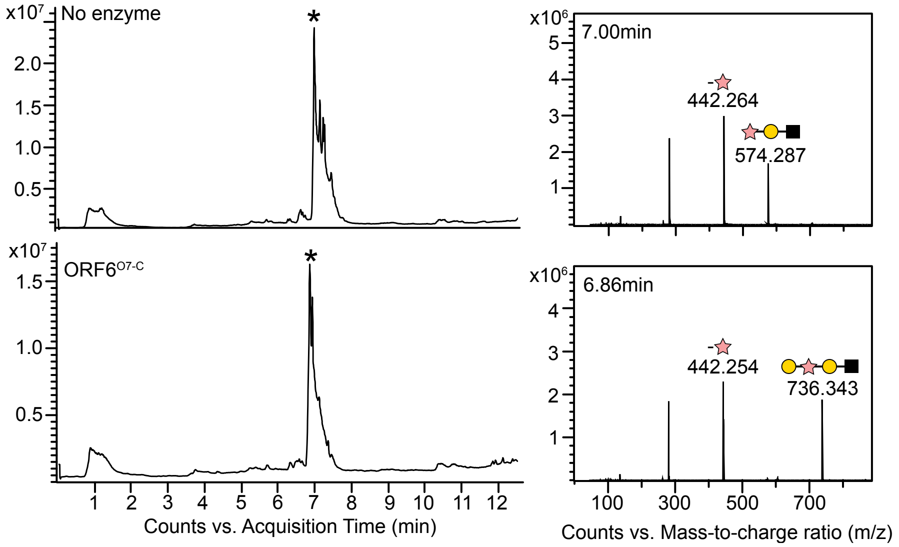

SI Figure 3. MS data for the reaction mixtures from Figure 2. The *left* panel shows total ion chromatograms with the biosynthetic reaction products eluting around the 7 min mark (indicated with an asterisk). The panel on the *right* shows the relevant regions from the ESI scans (positive ion mode) obtained from the peak marked with an asterisk within the total ion chromatogram. The products are shown on the appropriate mass spectra with the exact mass of acceptor **1** is 573.28 and a mass increase of 163 consistent with the addition of galactose to **1**.

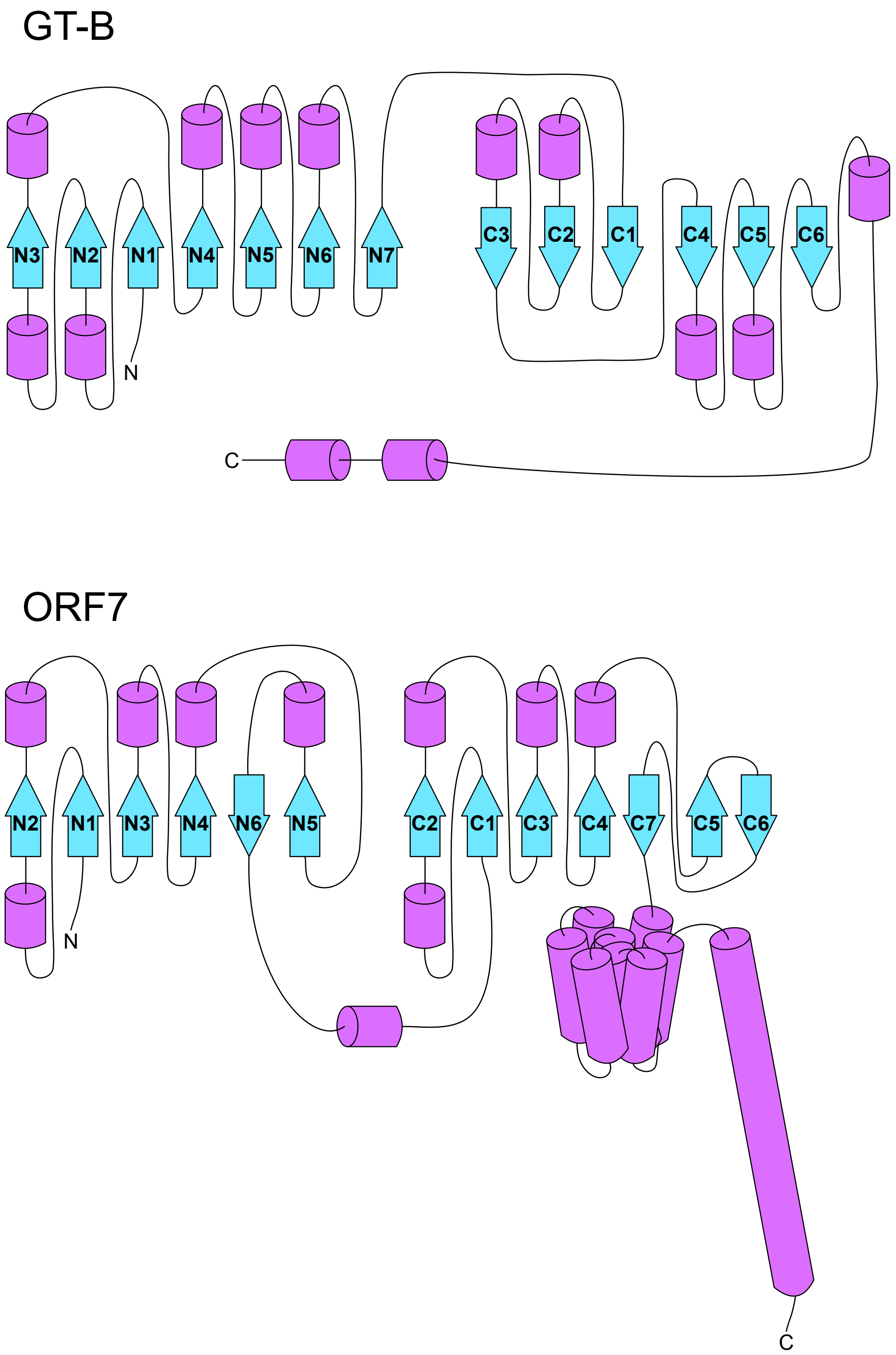

SI Figure 4. Topological diagram of the AlphaFold model of ORF7^{O4} compared to the GT-B GT fold. ORF7^{O4} displays key differences from the canonical GT-B fold including a TPR-like α-helical bundle that precedes the coiled-coil and two β/α/β domains that do not follow the usual Rossmann fold of GTs. Both domains contain a switch in the orientation of central β-sheet after the fourth strand, resulting in one antiparallel strand in the first domain, and two in the second domain

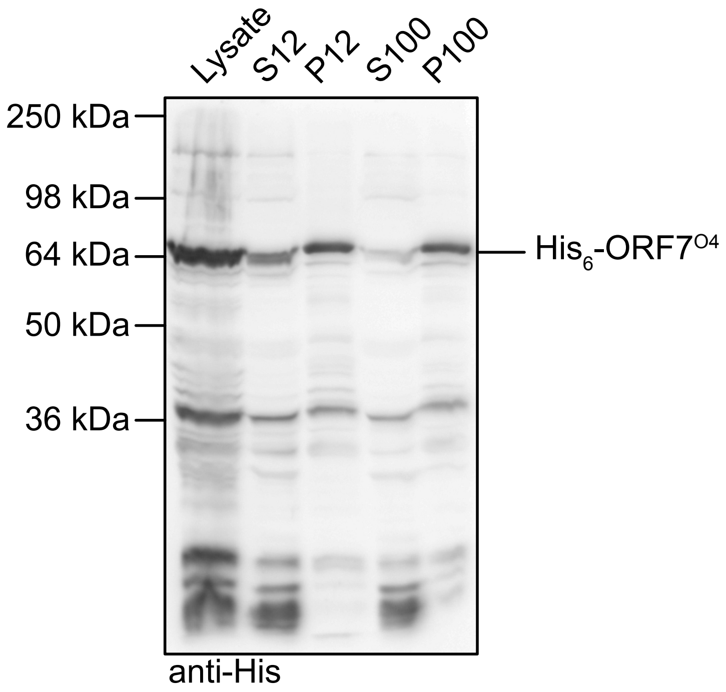

SI Figure 5. Western immunoblot using Anti-His_5 antibodies to probe the cellular localization of $ORF7^{O4}$. Cells expressing $ORF7^{O4}$ were lysed by sonication and sequential centrifugation steps were performed to fractionate the lysate and determine the localization of $ORF7^{O4}$ Centrifugation at 12,000 x *g* was performed to separate insoluble protein and cellular debris (P12) from cytosolic and membrane proteins (S12). Centrifugation at 100,000 x *g* was then performed to separate the membranes (and their associated proteins – P100) from the soluble proteins (S100). $ORF7^{O4}$ (theoretical molecular weight of 81 kDa) was predominately detected in the P100 fraction, indicating its membrane association. The protein ladder shown is from SeeBlue plus2 (Thermo).

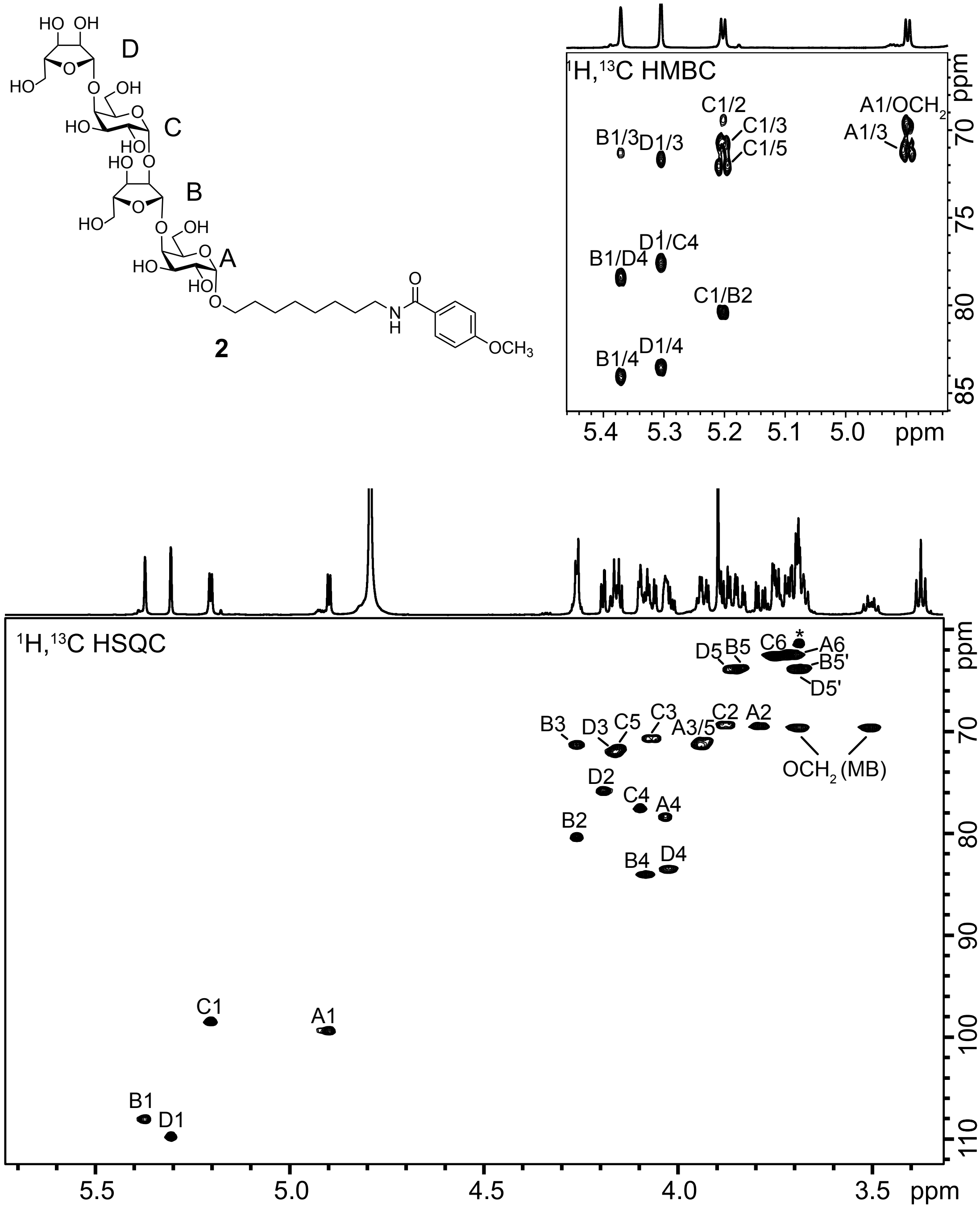

SI Figure 6. NMR spectroscopy of **2** (enzymatically synthesized from **1**). The determined structure is shown above with the residue labels. Resonance assignments are indicated on the HSQC spectrum. An HMBC experiment was used to confirm the linkages between sugar units and are indicated on the spectrum. MB represents the octyl methoxybenzamide tag of the acceptor and an asterisk indicates an unknown impurity.

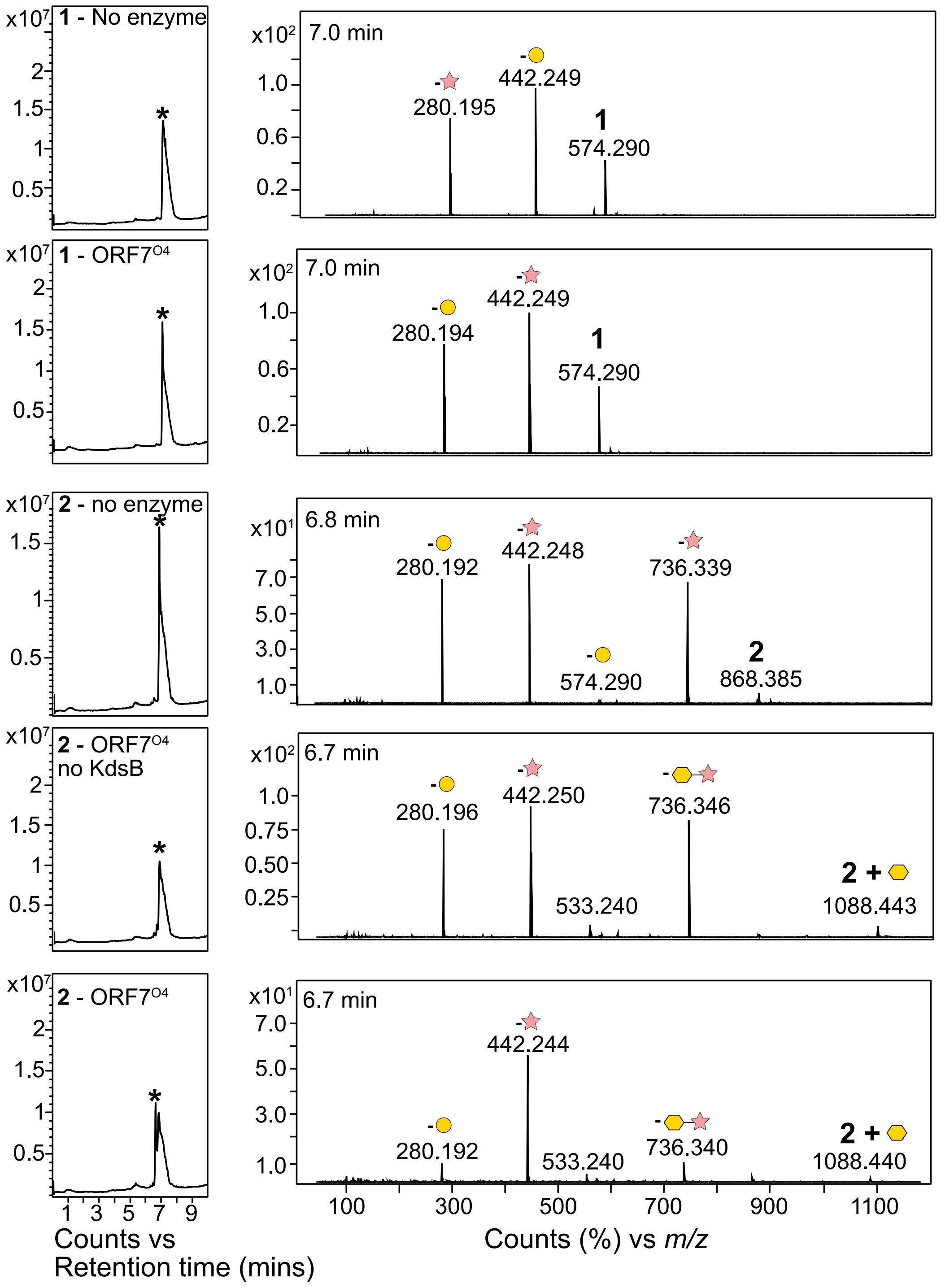

SI Figure 7. Mass spectrometry data for the reaction mixtures from Figure 3. The *left* panel shows total ion chromatograms with the biosynthetic reaction products eluting around the 7 min mark (indicated with an asterisk). The panel on the *right* shows the relevant regions from the ESI scans (positive mode) obtained from the selected point marked with an asterisk within the total ion chromatogram. The structures are indicated on the mass spectrum. Disaccharide **1** has an exact mass of 573.28 while tetrasaccharide **2** has an exact mass of 867.37. The addition of Kdo corresponds to an increase of 220.

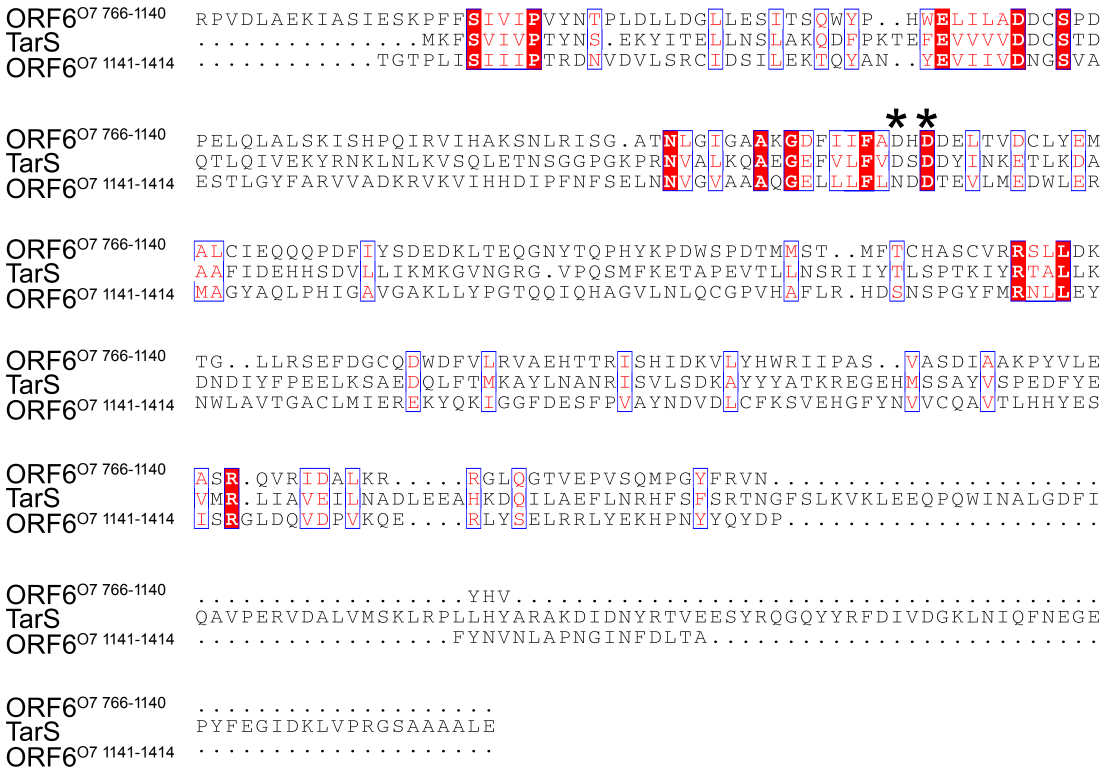

SI Figure 8. Multiple sequence alignment of the two GT2 domains from ORF6^{O7} with the characterized TarS GT2 representative (A0A0H3JPC6). The sequences share little similarity, with the exception of the conserved metal binding site. GT2 enzymes use a DXD (or a modified version) to bind a divalent cation for the coordination of nucleotide diphosphate linked sugars. This region is indicated with asterisks showing a DXDD motif (D975, D977, D978) in ORF6$^{O7\text{-}766\text{-}1140}$ and an NXD motif (N1230A, D1232A) in ORF6$^{O7\text{-}1141\text{-}1414}$. These residues were targeted for mutagenesis.

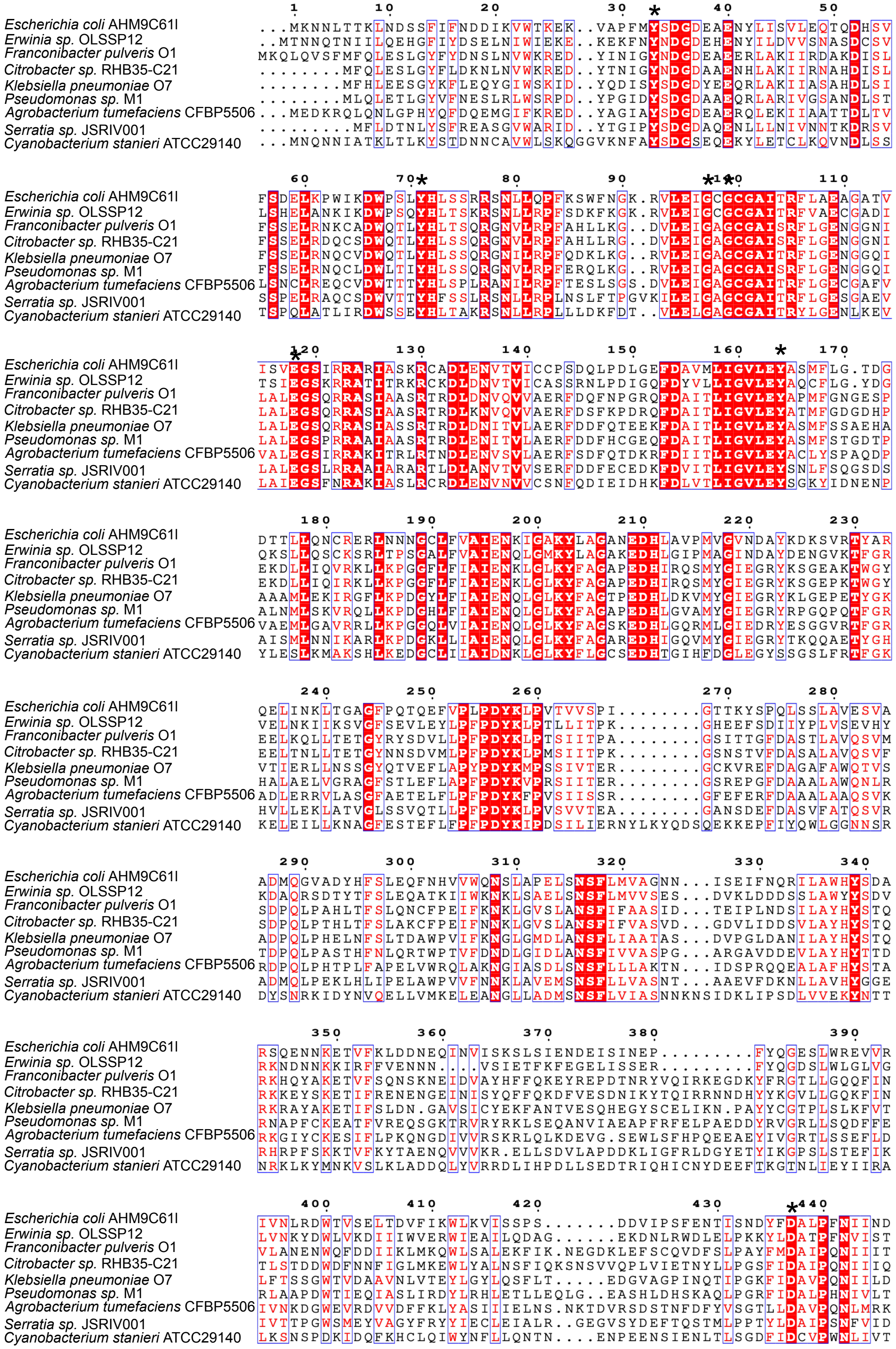
Escherichia coli AHM9C61I
Erwinia sp. OLSSP12
Franconibacter pulveris O1
Citrobacter sp. RHB35-C21
Klebsiella pneumoniae O7
Pseudomonas sp. M1
Agrobacterium tumefaciens CFBP5506
Serratia sp. JSRIV001
Cyanobacterium stanieri ATCC29140

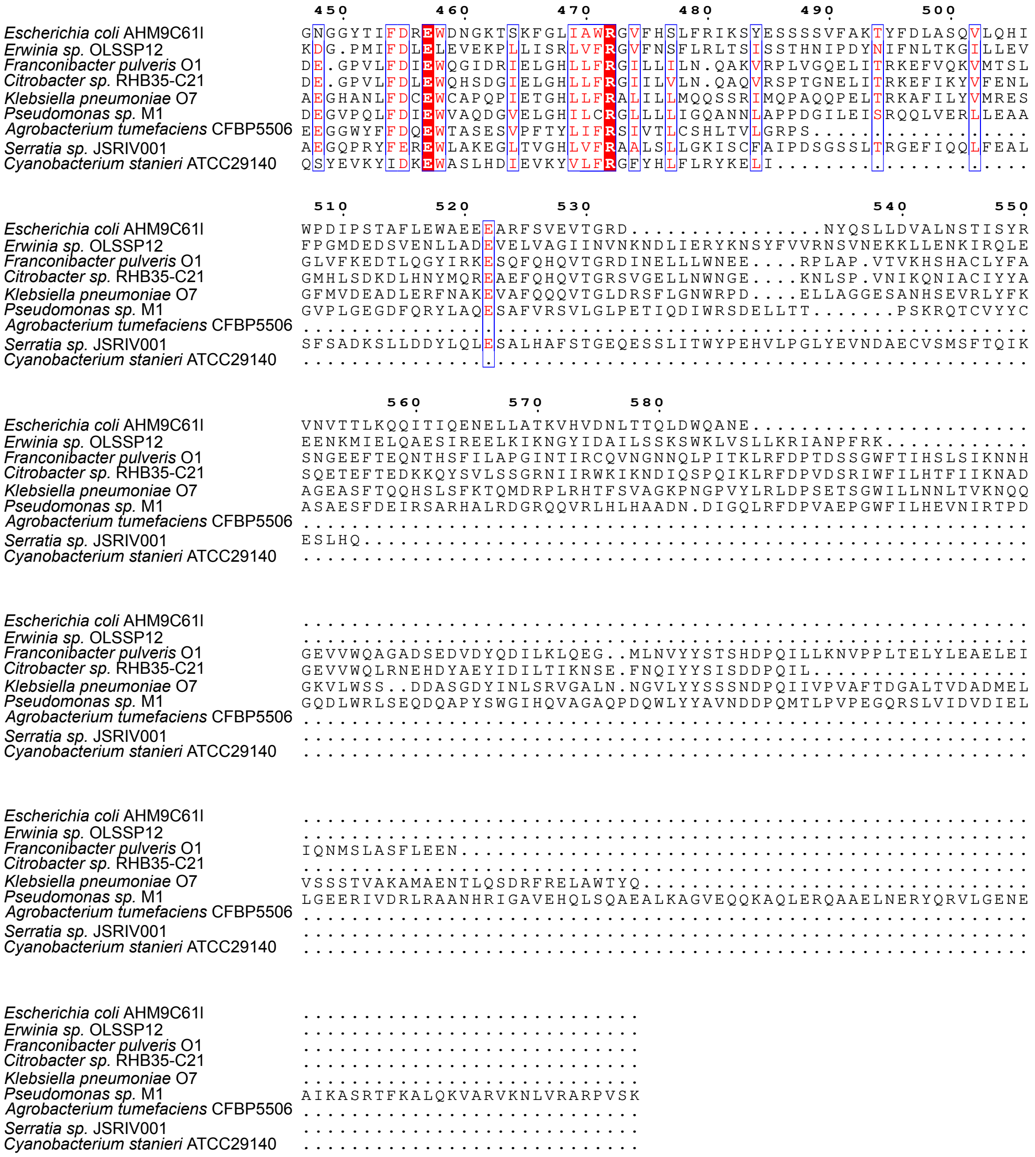

SI Figure 9. Multiple sequence alignment of selected ORF6^{O7-N} orthologs. The orthologs were all encoded by polysaccharide clusters. Orthologs shown are from *Escherichia coli* AHM9C61I (WMX77427.1), *Erwinia sp.* OLSSP12 (A0A2G8E4A3), *Franconibacter pulveris* O1 (ANF28855.1), *Citrobacter sp.* RHB35-C21 (QMD52189.1), *Pseudomonas sp.* M1 (UNY89431.1), *Agrobacterium tumefaciens* CFBP5506 (WGM60854.1), *Serratia sp.* JSRIV001 (UAN47652.1), *Cyanobacterium stanieri* ATCC29140 (K9YNL9). The asterisks are the residues shown in the model in Figure 6b. Alignments were performed in ClustalW and visualized with ESPript.

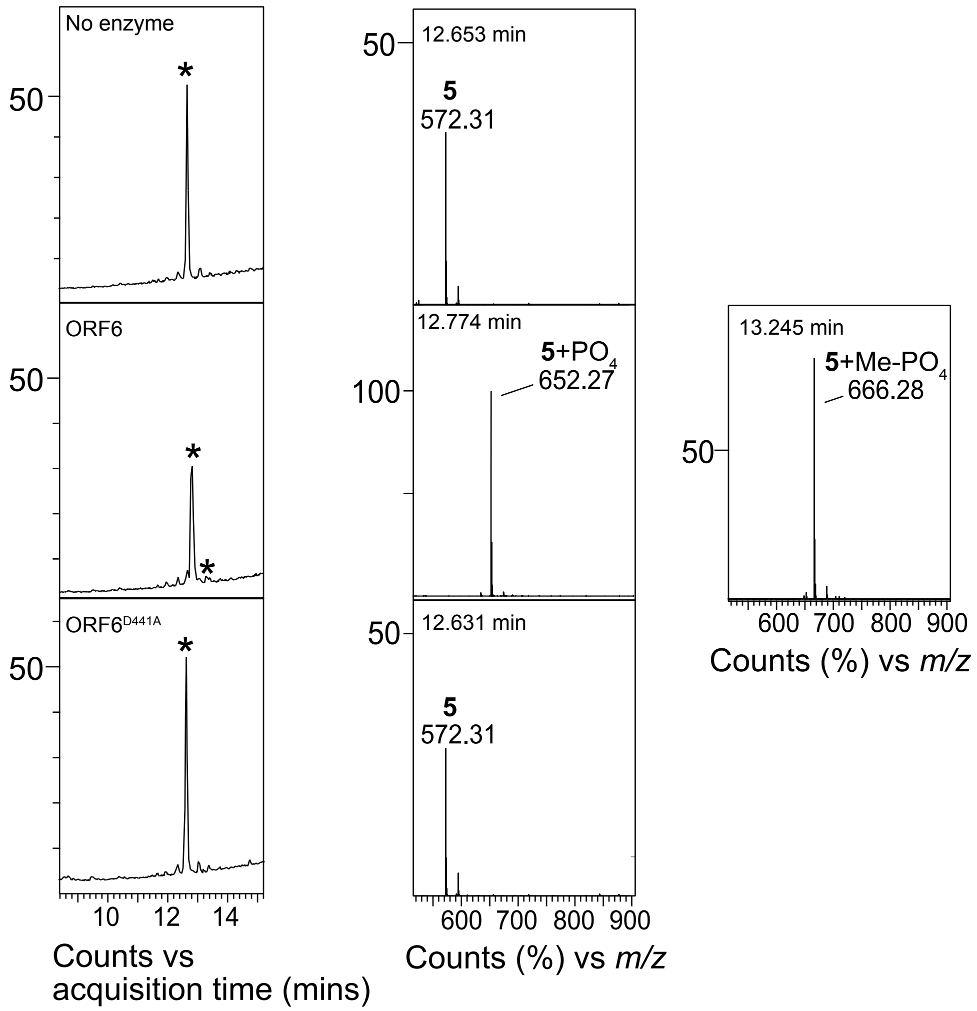

SI Figure 10. MS data for the reaction mixtures from Figure 6c. The *left* panel shows total ion chromatograms with the biosynthetic reaction products eluting around the 12.5min/13min mark (indicated with an asterisk). The panel on the *right* shows the relevant regions from the ESI scans (positive mode) obtained from the selected points marked with an asterisk within the total ion chromatogram. The structures are indicated on the mass spectrum. Disaccharide **5** has an exact mass of 571.30. The addition of PO_4 corresponds to an increase of 80 and $MePO_4$ corresponds to an exact mass of 94.

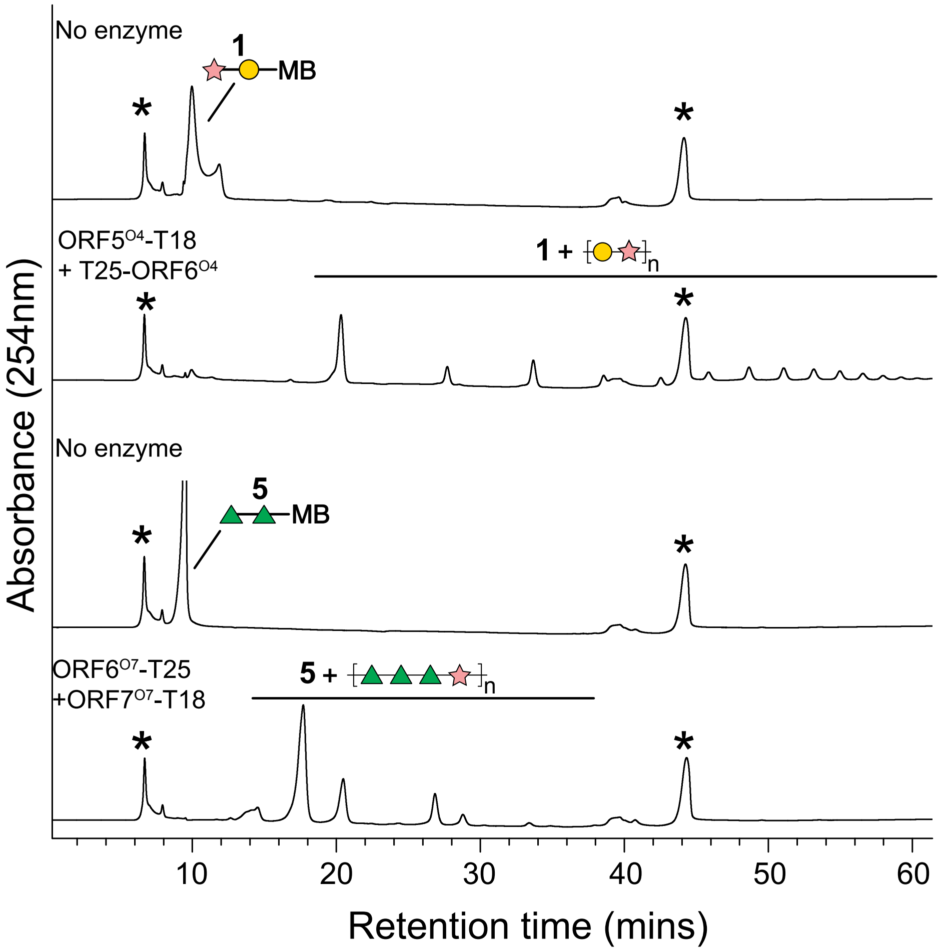

SI Figure 11. HPLC separation of the contents of *in vitro* reactions performed using whole cell lysates of *E. coli* BTH101 expressing either ORF5^{O4}-T18 and T25-ORF6^{O4} or ORF6^{O7}-T25 and ORF7^{O7}-T18. BTH101 expressing empty vectors were used as control lysates. Reactions were performed overnight at room temperature, after which they were purified by SepPak, dried and then analyzed by HPLC. Peaks marked by an asterisk were contaminants that were present in all samples. Incubation of **1** with lysate containing ORF5^{O4}-T18 and T25-ORF6^{O4} led to the depletion of acceptor and the appearance of new later eluting peaks, consistent with polymer production. Likewise, incubation of **5** with lysate containing ORF6^{O7}-T25 and ORF7^{O7}-T18 led to the depletion of acceptor and the appearance of new peaks, consistent with polymer production.

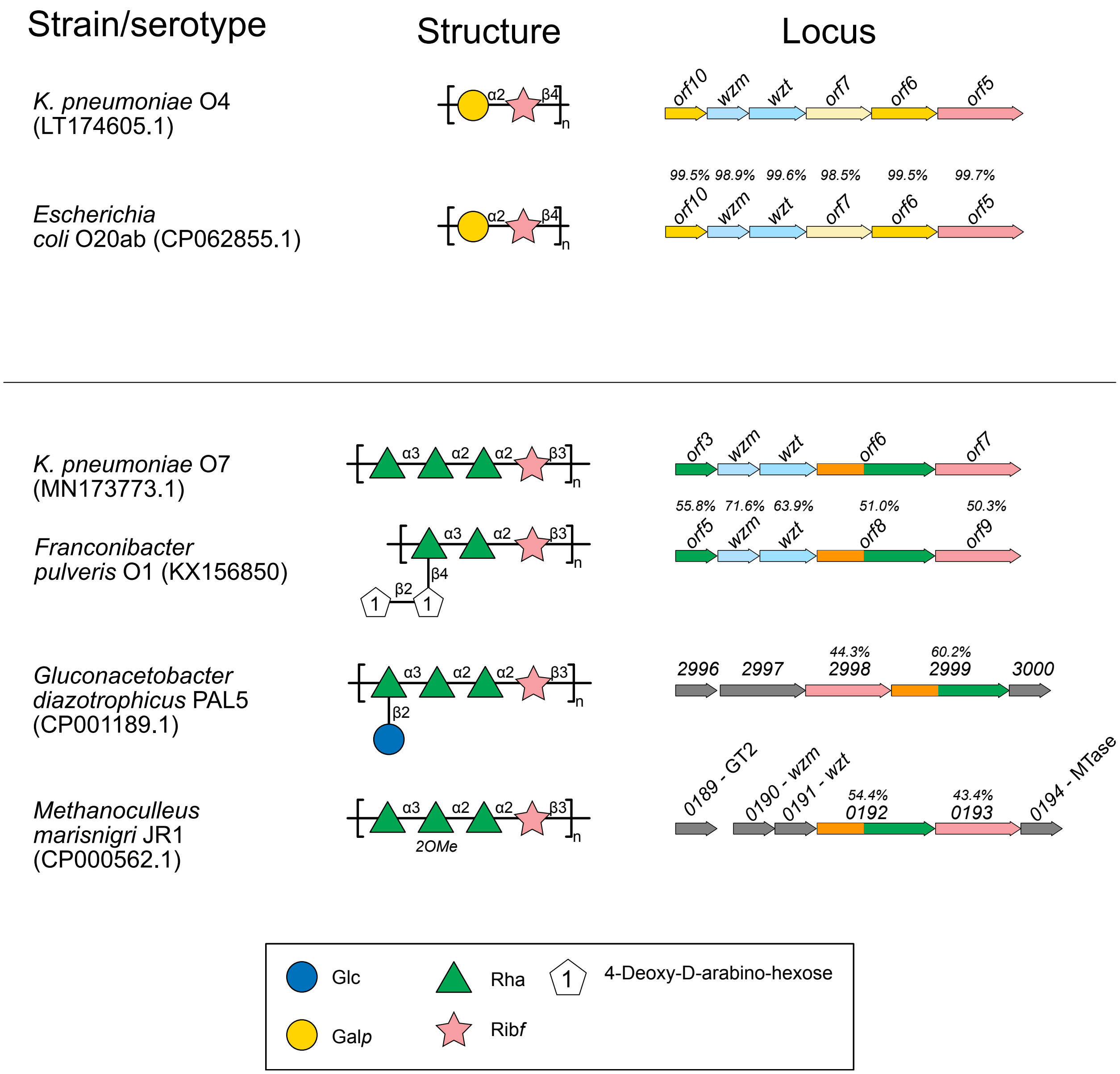

SI Figure 12. Published structures with similar or identical structures to *K. pneumoniae* O4 and O7 and their corresponding genetic loci from genomic sequences (Accession numbers are provided with the species/strain names). The carbohydrate structure database (http://csdb.glycoscience.ru) was used to search for similar structures to O4 and O7. In all cases, the polymerase/terminator components were identified, but genes encoding components such as an ABC transporter or candidate adaptor GTs were not always found. Values for percent identity of the encoded proteins (where possible) are shown above the gene designations. Genes shown in grey were still predicted to encode proteins involved in polysaccharide biosynthesis but did not show similarity to any of the encoded proteins of the O7 cluster.

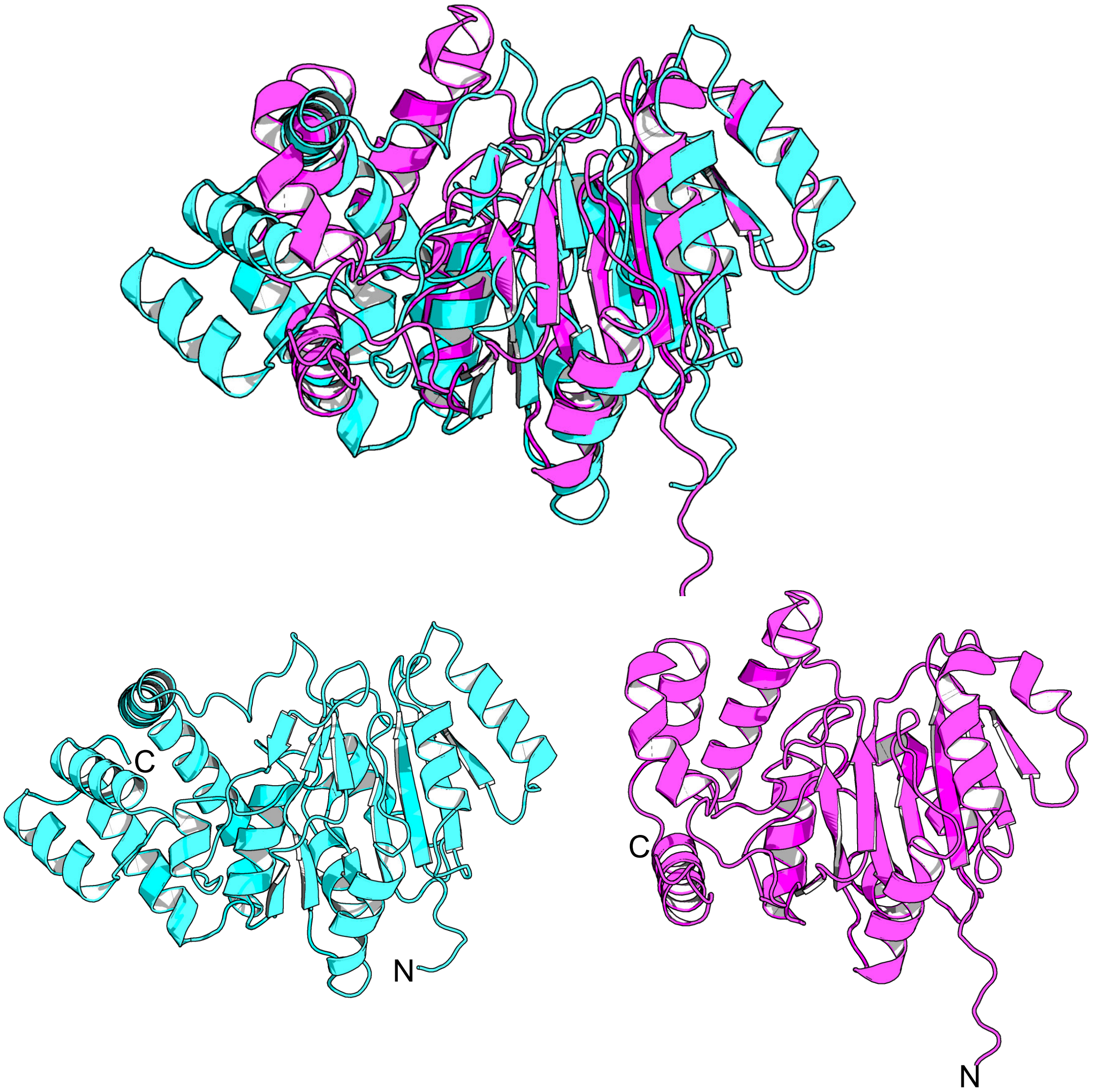

SI Figure 13. AlphaFold models of *K. pneumoniae* O12 WbbL (pink) and O7 ORF3 (blue). The structures are shown as an overlay and separated side-by-side. The structures share similarity in the central Rossmann fold (Z-score of 15.4, RMSD of 3.6Å, with significant divergence in the C-terminal α-helical domains.

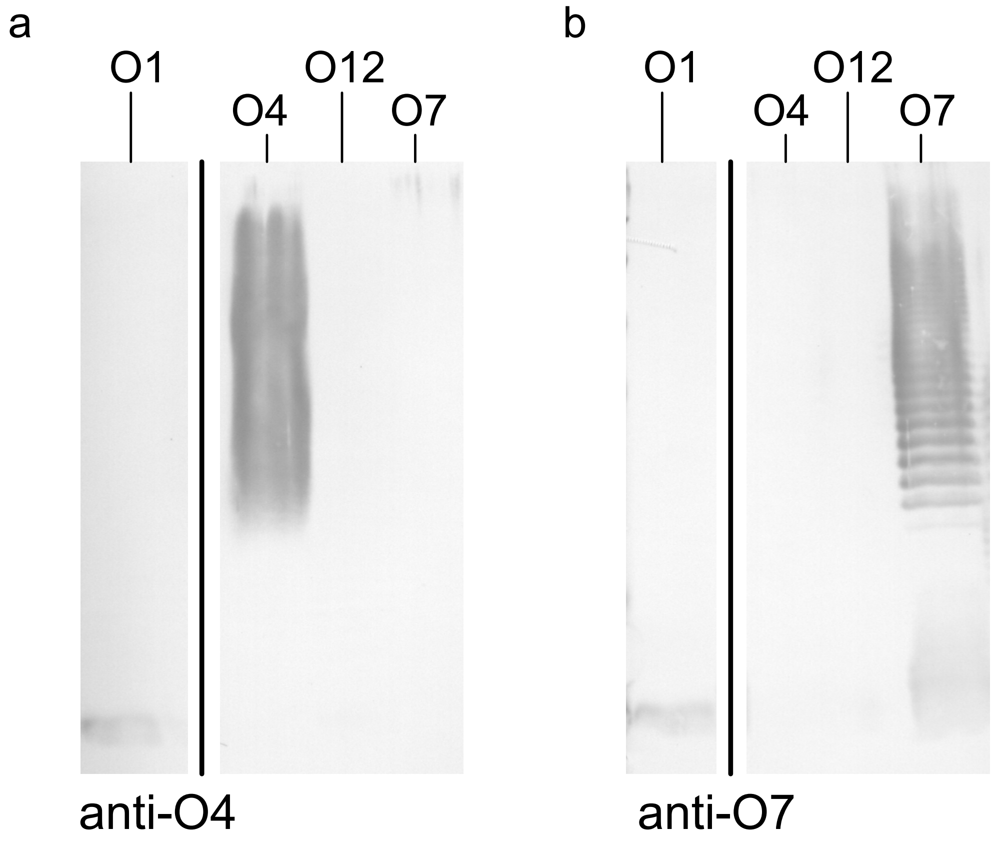

SI Figure 14. Western blot validation of anti-O4 (**a**) and anti-O7 (**b**) antisera. A selection of *K. pneumoniae* serotypes were used to assess the specificity of each antiserum. Strains used were O1 (B5055), O4 (1702), O12 (708) and O7 (264-1). The final blot was spliced to only show relevant lanes, with the splice point indicated with a black line.

SI Table 1. Chemical structures of compounds used, and products made in *in vitro* reactions.

Compound number	Chemical Structure	SNFG representation
**1**	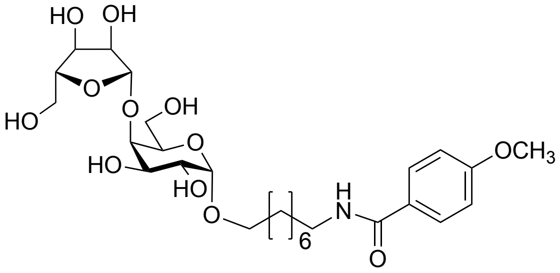	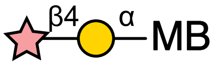
**2**	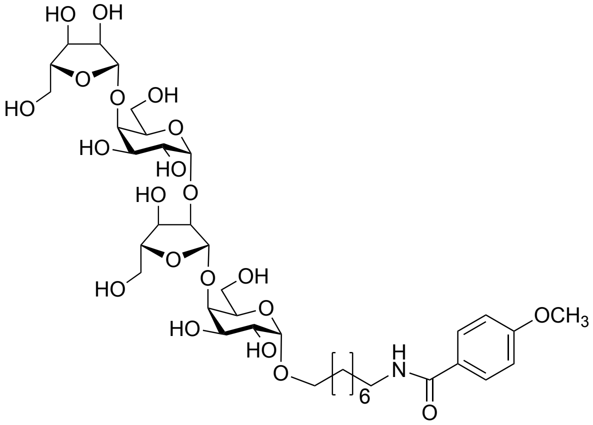	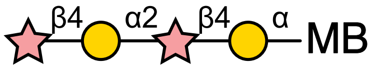
**3**	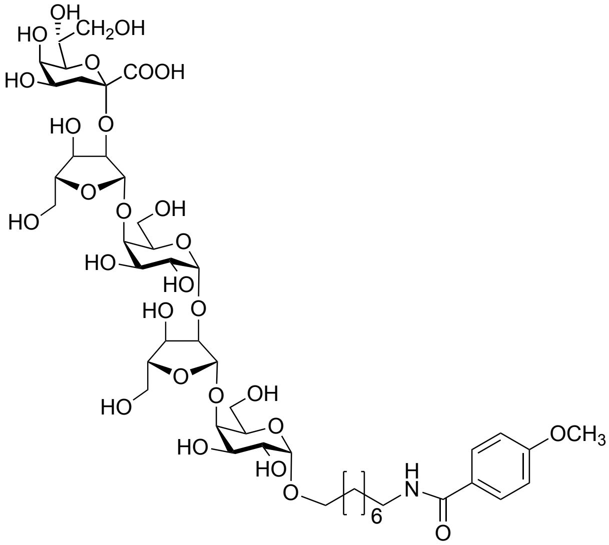	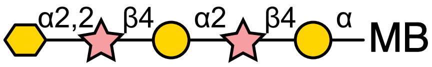

**4**	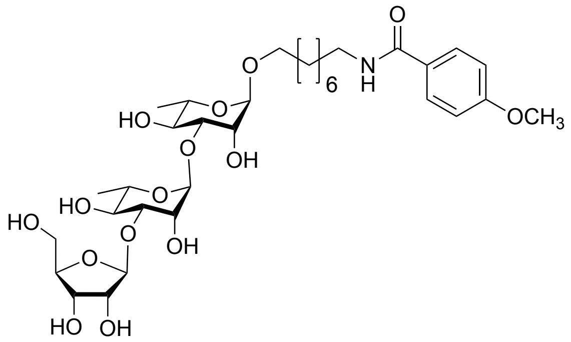	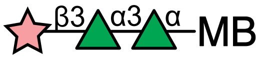
**5**	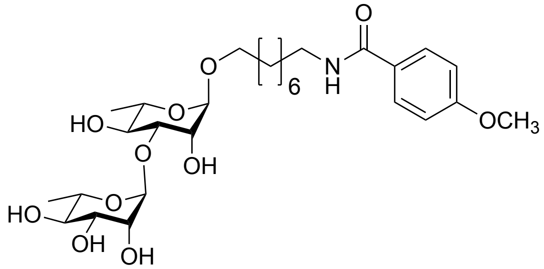	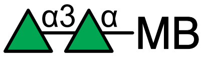
**6**	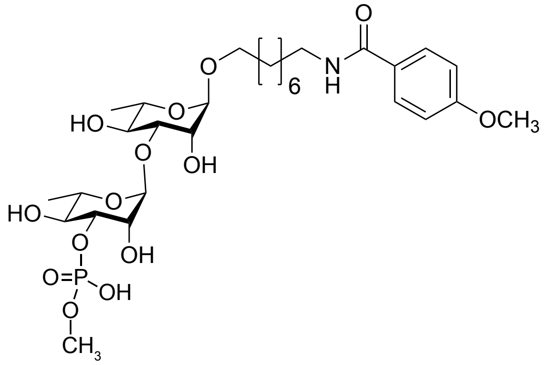	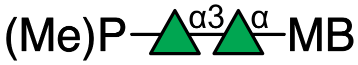

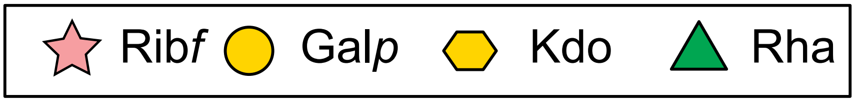

SI Table 2: ^{1}H and ^{13}C NMR chemical shifts for the *K. pneumoniae* O4 polysaccharide synthesized in *in vitro* reactions

Sugar Residue		Chemical shift (ppm)					
		H-1	*H-2*	*H-3*	*H-4*	*H-5 (5a,b)*	*H-6 (6a,b)*
		C-1	C-2	C-3	C-4	C-5	C-6
Repeat Unit							
→2)-β-ᴅ-Rib*f*-(1→	**A**	*5.40*	*4.27*	*4.27*	*4.09*	*3.85, 3.70*	
		107.9	80.4	71.4	84.1	63.5	
→4)-α-ᴅ-Gal*p*-(1→	**B**	*5.20*	*3.86*	*4.07*	*4.11*	*4.16*	*3.76, 3.75*
		98.5	69.3	70.5	78.2	71.9	62.5

SI Table 3: ^{1}H and ^{13}C NMR chemical shifts for **2**

Sugar Residue		Chemical shift (ppm)					
		H-1	*H-2*	*H-3*	*H-4*	*H-5 (5a,b)*	*H-6*
		C-1	C-2	C-3	C-4	C-5	C-6
→4)-α-ᴅ-Gal*p*-(1→MB	**A**	*4.90*	*3.79*	*3.94*	*4.03*	*3.93*	*3.71*
		99.5	69.6	71.4	78.5	71.0	62.5
→2)-β-ᴅ-Rib*f*-(1→	**B**	*5.37*	*4.26*	*4.26*	*4.09*	*3.86, 3.68*	
		108.1	80.5	71.5	84.2	64.0	
→4)-α-ᴅ-Gal*p*-(1→	**C**	*5.20*	*3.88*	*4.07*	*4.10*	*4.16*	3.75
		98.6	69.49	70.8	77.7	71.9	62.8
β-ᴅ-Rib*f*-(1→	**D**	*5.31*	*4.19*	*4.16*	*4.03*	*3.87, 3.69*	
		109.9	75.96	72.1	83.6	64.0	

SI Table 4: ^{1}H and ^{13}C NMR chemical shifts for **3**

Sugar Residue		Chemical shift (ppm)							
		H-1	*H-2*	*H-3*	*H-4*	*H-5 (5a,b)*	*H-6*	*H-7*	*H-8a,b*
		C-1	C-2	C-3	C-4	C-5	C-6	C-7	C-8
→4)-α-ᴅ-Gal*p*-(1→MB	**A**	*4.90*	*3.79*	*3.92*	*4.03*	*3.94*	*3.70*		
		99.5	69.5	70.9	78.5	71.3	62.5		
→2)-β-ᴅ-Rib*f*-(1→	**B**	*5.37*	*4.26*	*4.25*	*4.08*	*3.84, 3.67*			
		108.2	80.3	71.4	84.1	63.8			
→4)-α-ᴅ-Gal*p*-(1→	**C**	*5.20*	*3.94*	*3.95*	*4.01*	*4.14*	3.76		
		98.4	69.4	70.8	77.7	72.2	62.5		
→2)-β-ᴅ-Rib*f*-(1→	**D**	*5.32*	*4.20*	*4.20*	*4.04*	*3.81, 3.66*			
		108.5	77.1	71.0	84.6	63.3			
α-ᴅ-Kdo*p*-(2→	**E**			*2.20, 1.81*	*4.15*	*4.03*	*4.00*	*3.96*	*3.96, 3.72*
		176.2	101.2	35.5	67.2	67.4	72.6	70.8	64.3

SI Table 5: ^{1}H and ^{13}C NMR chemical shifts for *K. pneumoniae* O7 polysaccharide synthesized in *in vitro* reactions

Sugar Residue		Chemical shift (ppm)					
		H-1	*H-2*	*H-3*	*H-4*	*H-5 (5a,b)*	*H-6*
		C-1	C-2	C-3	C-4	C-5	C-6
Repeat unit							
→2)-β-ᴅ-Rib*f*-(1→	**A**	*5.35*	*4.22*	*4.44*	*4.07*	*3.87, 3.71*	
		108.2	81.9	71.0	83.8	63.0	
→3)-α-ᴅ-Rha*p*-(1→	**B**	*5.06*	*4.24*	*3.91*	*3.53*	*3.90*	*1.31*
		103.3	71.1	79.9	72.3	70.4	18.0
→3)-α-ᴅ-Rha*p*-(1→	**C**	*4.98*	*4.18*	*3.85*	*3.57*	*3.80*	*1.29*
		103.2	71.1	79.4	72.5	70.7	17.9
→2)-α-ᴅ-Rha*p*-(1→	**D**	*5.15*	*4.10*	*3.93*	*3.53*	*3.76*	*1.32*
		100.9	79.3	71.0	73.4	70.7	18.0

SI Table 6: ^{1}H and ^{13}C NMR chemical shifts for **6**

Sugar Residue		Chemical shift (ppm)					
		H-1	*H-2*	*H-3*	*H-4*	*H-5*	*H-6*
		C-1	C-2	C-3	C-4	C-5	C-6
Me/MeP→3)-α-ᴅ-Rha*p*-(1→	**A**	*5.18*	*4.25*	*4.29*	*3.59*	*3.87*	*1.29*
		103.2	70.9	75.9	72.6	70.3	18.0
→3)-α-ᴅ-Rha*p*-(1→	**B**	*4.75*	*4.01*	*3.79*	*3.56*	*3.72*	*1.27*
		100.8	71.3	79.3	72.6	70.0	18.0

SI Table 7: Bacterial strains and plasmids.

**Strain or plasmid**	**Description**	**Reference**
E. coli DH5α	K-12 F⁻ φ80*lacZ* çM15 Δ(*lacZYA-argF*) U169 *deoR recA1 endA1 hsdR17* (r_K^-, m_K^+) *gal*⁻ *phoA supE44 thi*⁻ *gyrA96 relA1*	(1)
E. coli Top10	F⁻, *mcrA*, Δ(*mrr-hsdRMS-mcrBC*), φ80, *lacZΔM15*, Δ*lacX74*, *deoR*, *nupG*, *recA1*, *araD139*, Δ(*ara-leu*)7697, *galU*, *galK*, *repsL*(Str^R), *endA1*	Invitrogen
E. coli BL21 (DE3)	F⁻ *ompT hsdS*$_B$ (r_B^-, m_B^-) *gal dcm* (DE3)	NEB
K. pneumoniae 264-1 (O7:K$^-$)	Acapsular mutant of wildtype O7 serotype	F. Ørskov
K. pneumoniae 1702 (O4:K$^-$)	Acapsular mutant of wildtype O4 serotype	F. Ørskov
E. coli CWG1219	*E. coli* Top10 derivative Δ*wzx-wbbK* Δ*gtrA*	(2)
E. coli CWG286	K-12 *lacZ trp* Δ(*sbc-rfb*) *upp rel rpsL galE::Tn10*	(3)
pACYC184	Cloning vector Cm^r, Tc^r	(4)
pET28a(+)	Cloning vector with Isopropyl β-d-1-thiogalactopyranoside-inducible promoter	Novagen
pBAD24	Cloning vector with ʟ-arabinose-inducible promoter; Ap^r	(5)
pWQ573	pBAD24 derivative containing a selectable Cm resistance marker; Cm^r	(6)
pKT25	pACYC184 derivative for N-terminal fusion of the T25 fragment of adenylate cyclase from *B. pertussis*.	(7)

pKNT25	pACYC184 derivative for C-terminal fusion of the T25 fragment of adenylate cyclase from *B. pertussis*.	(7)
pUT18	pBluescript II KS derivative for C-terminal fusion of the T18 fragment of adenylate cyclase from *B. pertussis*	(7)
pUT18C	pBluescript II KS derivative for N-terminal fusion of the T18 fragment of adenylate cyclase from *B. pertussis*	(7)
pKT25-ZIP	pKT25 derivative containing a 35-residue leucine zipper for positive control	(7)
pUT18C-ZIP	pUT18C derivative containing a 35-residue leucine zipper for positive control	(7)
pWQ516	pACYC184 derivative containing *K. pneumoniae* O2a cluster with *wbbO* deletion	(8)
pWQ1146	pACYC184 derivative containing O4 cluster (*orf10-5*) from *K. pneumoniae* 1702.	This study
pWQ1147	pWQ1146 derivative with $orf7^{O4}$ deletion	This study
pWQ1148	pBAD24 derivative for expression of $ORF10^{O4}$-His_6	This study
pWQ1149	pBAD24 derivative for expression of $ORF7^{O4}$-His_6	This study
pWQ1150	pET28a(+) derivative for expression of $ORF6^{O4}$-His_6	This study
pWQ1072	pET28a(+) derivative for expression of $ORF5^{O4}$-His_6	(9)
pWQ1151	pACYC184 derivative containing *K. pneumoniae* O7 cluster (*orf3-7)* from *K. pneumoniae* 264-1.	This study
pWQ1152	pWQ1151 derivative containing *K. pneumoniae* O7 cluster with $orf6^{O7}$ deletion.	This study

pWQ1153	pWQ573 derivative expressing *K. pneumoniae* O12 cluster with *wbbL* deletion.	This study
pWQ1154	pBAD24 derivative for expression of ORF3^{O7}	This study
pWQ1155	pBAD24 derivative for expression of ORF6^{O7}-His$_6$	This study
pWQ1069	pBAD24 derivative for expression of ORF7^{O7}-His$_6$	(9)
pWQ1156	pET28a(+) derivative for expression of ORF6$^{O7\text{-}766\text{-}1414}$ (ORF6$^{O7\text{-}C}$)-His$_6$	This study
pWQ1157	pET28a(+) derivative for expression of ORF6$^{O7\text{-}C\ D975A,D977A,D978A}$-His$_6$	This study
pWQ1158	pET28a(+) derivative for expression of ORF6$^{O7\text{-}C\ N1230A,D1232A}$-His$_6$	This study
pWQ1159	pWQ1155 derivative for expression of ORF6$^{O7\ D441A}$-His$_6$	This study
pWQ1160	pWQ1155 derivative for expression of ORF6$^{O7\ G91A,G93A}$-His$_6$	This study
pWQ1161	pKT25 derivative for expression of T25-ORF6^{O7}	This study
pWQ1162	pKNT25 derivative for expression of ORF6^{O7}-T25	This study
pWQ1163	pUT18 derivative for expression of ORF7^{O7}-T18	This study
pWQ1164	pUT18-C derivative for expression of T18-ORF7^{O7}	This study
pWQ1165	pKT25 derivative for expression of T25-ORF6^{O4}	This study

pWQ1166	pKNT25 derivative for expression of ORF6^{O4}-T25	This study
pWQ1167	pUT18 derivative for expression of ORF5^{O4}-T18	This study
pWQ1168	pUT18-C derivative for expression of T18-ORF5^{O4}	This study

SI Table 8: Oligonucleotide primers

Plasmid/Strain Generated	Primer Sequence Forward/ Reverse[a-c]	Description
pWQ1146	5′- aaaagaaaattctatcgcaagtattaaGTCGACCGATGCCCTTGAGAG-3′/<br>5′- ttttatgcatttaatttttatttaaccttgGATCCACAGGACGGGTGTGG-3′	pACYC184 fragment primers for the Gibson assembly of O4 cluster
	5′- ccacacccgtcctgtggatccCAAGGTTAAATAAAAATTAAATGCATAAAAACTC-3′/<br>5′- ctctcaagggcatcggtcgacTTAATACTTGCGATAGAATTTTCTTTTATAAAAAAC -3′	insert fragment primers for the Gibson assembly of O4 cluster
pWQ1147	5′-TTCTATTGTGAAAGGTTATAATG-3′/<br>5′-CAACTTACCACTCTGCAG-3′	Inverse PCR primers to delete *orf7*^{O4} from pWQ1146
pWQ1148	5′-gatcccatggggAGGATAGTGTATTTTGTCAATGCAGC-3′/<br>5′-gatcaagcttttagtgatggtgatggtgatgCCGCTTTGTTGTACTTAGTATTTTATTG-3′	Primers to clone O4 *orf10* into NcoI-HindIII of pBAD24 and express with C-his$_6$.
pWQ1149	5′-acccgtttttttgggctagcaggaggaattcaccATGAATGATATTTCAATAACTGATTATCTCGGG-3′/<br>5′-tcttctctcatccgccaaaacagccaagcttttagtgatggtgatggtgatgTTTTATATTTAGCATGCGTAGTAGATGCC-3′	Primers for Gibson assembly of O4 *orf7* into EcoRI-HindIII of pBAD24 and express with C-his$_6$.
pWQ1150	5′-gatcccatgggaGCTAATATTGCTTGGTTTATACC-3′/<br>5′-gatcgcggccgcttagtgatggtgatggtgatgATTCTTCAACTTAAAATAAAGCGCG-3′	Primers to clone O4 *orf6* into NcoI-NotI of pET28a(+) and express with C-his$_6$
pWQ1151	5′-cgccagtcactatggcgtgctgctagcCGATGCTGAAGTATATAATTAATTCATG-3′/<br>5′-cctgacatatctggttgtacttTCCAAATTATTTATCAACCGAACTAC-3′	Primers for Gibson fragment 1 of O7 cluster in BamHI/SalI of pACYC184
	5′-aagtacaaccagatatgtcaggTGAGTATATCTGCGTTAAATATTGCA-3′/<br>5′-ggctgctgctgctcgatGCACAATGCCATTTCATACAAGCAG-3′	Primers for Gibson fragment 2
	5′-atcgagcagcagcagccTGATTTTATTTACAGTGATGAAGATAAG-3′/<br>5′-gatgcgtccggcgtagaggatccAATTATAAATAATCTGTCGCAACCAGC-3′/	Primers for Gibson fragment 3
pWQ1152	5′-GTCATTTTTTGATTTTATATTT-3′/<br>5′-ATATTCAACCTATGCAATATTTAAC-3′	Inverse PCR primers to delete *orf6* from pWQ1151
pWQ1153	5′-aaaaaaaaggtaccaggaggaattcAAAATGAAAAACCCGCATC-3′/<br>5′-aaaaaaaagcttCTAGCGGTTGCGCTTAAACTC-3′	Primers to clone *K. pneumoniae* O12 cluster with *wbbL* deletion into KpnI-HindIII of pWQ573

pWQ1154	5′-gtttctccatacccgtttttttgggctagcaggaggaattcaccATGcaccatcaccatcaccatAATATAAACGACAA AATTGCGATCC-3′/ 5′-ctctagaggatccccgggtaccatggTTAGAGCTTCTTTAAAAGAACGGCC-3′	Gibson primers to clone O7 *orf3* into NheI-NcoI in pBAD24 and express with N-His_6
pWQ1155	5′-gatcgctagcaggaggaattcaccATGTTTCATCTCGAAGAAAGTGGG-3′/ 5′-gatcccatggtcagtgatggtgatggtgatgGGCCGTCAGATCAAAGTTAATTCC-3′	Primers to clone O7 *orf6* into NheI-NcoI of pBAD24 and express with a C-His_6
pWQ1156	5′-gatcgctagcATGACAAGCTGGAAAGTGTCTCAG-3′/ 5′-gatcctcgagGGCCGTCAGATCAAAGTTAATTCC-3′	Primers to clone truncated O7 *orf6* into NheI-XhoI of pET28a(+) to express $ORF6^{O7-766-1414}$ with a C-His_6
pWQ1157	5′-GCGGCGAAAGGTGATTTTATTATTTTTGCCGcTCATGcCGcTGAGTTAACCGTTGACTGC-3′/ 5′-CATTTCATACAAGCAGTCAACGGTTAACTCAgCGgCATGAgCGGCAAAATAATAAAATCAC CTTTC-3’	Inverse PCR primers to mutate pWQ1156 to express $ORF6^{O7-C\ D975A,D977A,D978A}$-$His_6$
pWQ1158	5′-atgccACCGAAGTGCTGATG-3′/ 5′-cggcCAGGAAAAGCAGGAG-3′	Inverse PCR primers to mutate pWQ1156 to express $ORF6^{O7-C\ N1230A,\ D1232A}$-$His_6$
pWQ1159	5′-AAATTCATCGcTGCAGTGCCTC-3′/ 5′-CCCAGGGATAGTCTGATTAATC-3′	Inverse PCR primers to mutate pWQ1155 to express $ORF6^{O7\ D441A}$-His_6
pWQ1160	5′-GGCAGGGTGCTGGAAATCGcTGCCGcCTGCGGTGCGATTACCCGTTTTCTCGG-3′/ 5′-CGGGTAATCGCACCGCAGgCGGCAgCGATTTCCAGCACCCTGCCTTTTAGCTTATC-3′	Inverse PCR primers to mutate pWQ1155 to express $ORF6^{O7\ G91A,G93A}$-His_6
pWQ1161, pWQ1162	5′-GATCTCTAGAGatggctaatattgcttggtttatacctc-3′/ 5′-GATCCGGTACCCGattcttcaacttaaaataaagcgcgc-3′	Primers to clone *orf6*^{O4} into XbaI-KpnI of BACTH plasmids
pWQ1163, pWQ1164	5′-GATCTCTAGAGatgaataaatttaaactcaaaactgttgatgtatg-3′/ 5′-GATCCGGTACCCGatacttgcgatagaattttcttttataaaaaacc-3′	Primers to clone *orf5*^{O4} into XbaI-KpnI of BACTH plasmids
pWQ1165, pWQ1166	5′-GATCTCTAGAGatgtttcatctcgaagaaagtgggtataag-3′/ 5′-GATCCGGTACCCGggccgtcagatcaaagttaattcc-3′	Primers to clone *orf6*^{O7} into XbaI-KpnI of BACTH plasmids
pWQ1167, pWQ1168	5′-GATCTCTAGAGatggacgaaatttctatggaacagtttg-3′/ 5′-GATCCGGTACCCGtaaataatctgtcgcaaccagctg-3′	Primers to clone *orf7*^{O7} into XbaI-KpnI of BACTH plasmids

[a]Sequence complimentary to the template is in lower case
[b]Mutations introduced by mutagenesis primers are shown in lower case, with the rest of the primer shown in upper case
[c]Restriction enzyme cut sites are underlined

General Synthetic Methods

All reagents were purchased from commercial sources and were used without further purification unless noted. Reaction solvents were purified by successive passage through columns of alumina and copper under argon. Unless stated otherwise, all reactions were carried out at room temperature and under a positive pressure of argon and were monitored by TLC on Silica Gel G-25 F254 (0.25 mm). Visualization of the reaction components was achieved using UV fluorescence (254 nm) and/or by charring with acidified anisaldehyde solution in ethanol, acetic acid and sulfuric acid. Organic solvents were evaporated under reduced pressure, and the products were purified by column chromatography on silica gel (230−400 mesh). Optical rotations were measured at 22 ± 2 °C at the sodium D line (589 nm) in a microcell (10 cm, 1 mL) and are in units of deg·mL/(dm·g). ^{1}H NMR spectra were recorded at 500 MHz and chemical shifts are referenced to residual $CHCl_3$ (7.26 ppm, $CDCl_3$), CHD_2OD (3.30 ppm, CD_3OD). ^{13}C NMR spectra were recorded at 126 MHz and chemical shifts are referenced to $CDCl_3$ (77.0 ppm) or CD_3OD (49.3 ppm). Reported splitting patterns are abbreviated as s = singlet, d = doublet, t = triplet, m = multiplet, q = quartet, and dd = doublet of doublets. Assignments of NMR spectra were based on two-dimensional experiments (^{1}H−^{1}H COSY, HSQC). High-resolution ESI-MS spectra (time-of-flight analyzer) were recorded on an Agilent Technologies 6220 TOF spectrometer with samples suspended in CH_3OH or THF with added NaCl or NH_4Cl.

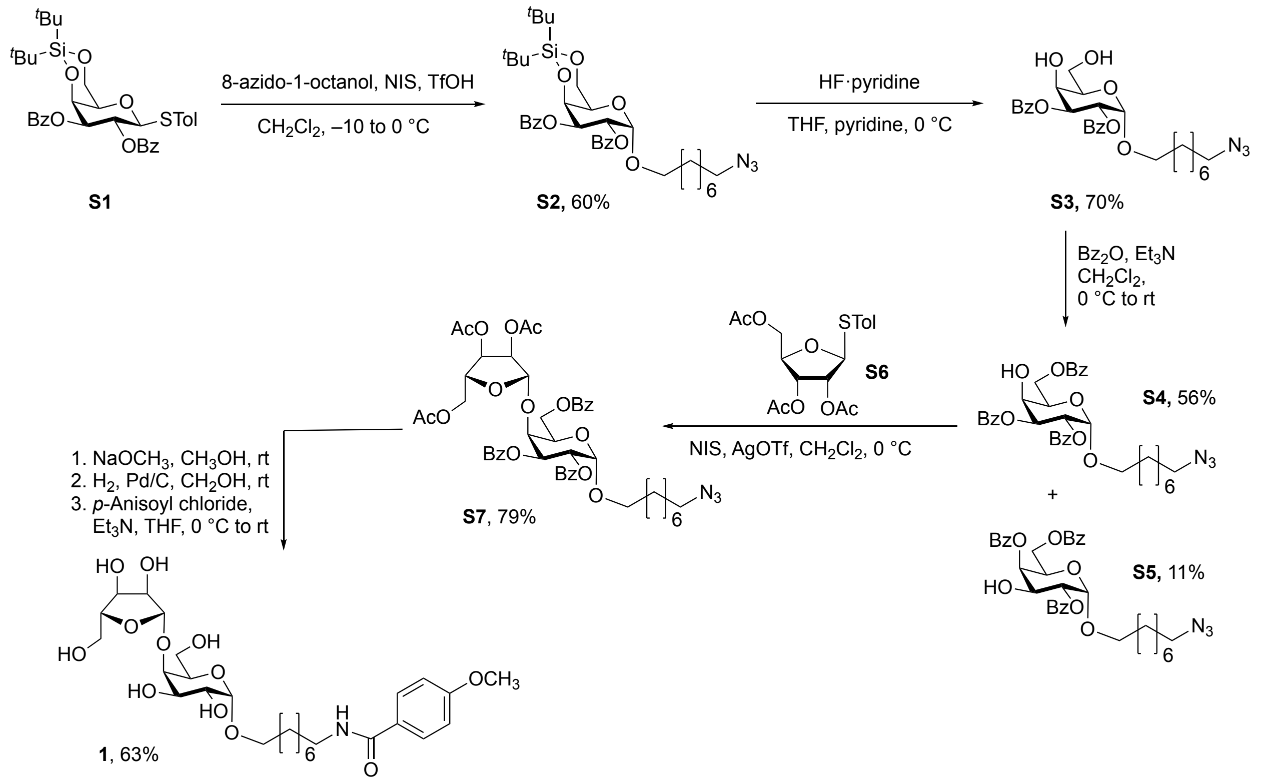

SI Scheme 1. Synthesis of **1**

Procedures and Data

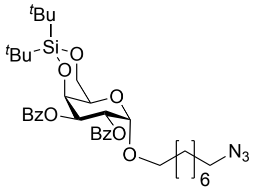

8-Azidooctyl 2,3-di-*O*-benzoyl-4,6-di-*O*-di-t-butylsilylidene-a-D-galactopyranoside (S2). Thioglycoside[1] (10) **S1** (390 mg, 0.615 mmol) and 8-azidooctanol (11) (526 mg, 3.074 mmol) were dried overnight under vacuum and then dissolved in CH_2Cl_2 (10 mL). After the mixture was stirred for 10 min at room temperature over 4 Å molecular sieves (freshly activated), the solution was cooled to –10 °C and stirred for 5 min. To the cooled reaction mixture were sequentially added *N*-iodosuccinimide (277 mg, 1.23 mmol) and TfOH (6 mL, 0.061 mmol) and the reaction mixture stirred for 4 h at 0 °C. The reaction mixture was diluted with CH_2Cl_2 (100 mL) and 5% $Na_2S_2O_3$ (200 mL) and satd aq $NaHCO_3$ (100 mL) were added to reaction mixture, which was stirred for 5–10 min at 0–5 ^oC, before

the solution was filtered through Celite. Next, the organic layer was separated, dried with Na_2SO_4, filtered and concentrated to give a crude product that was purified by chromatography (5% to 12% EtOAc–Hexane) to afford **S2** (250 mg, 60%) as an oil: $[\alpha]_D^{23}$ +128.9 (*c* = 0.7, CH_2Cl_2) ^{1}H NMR (500 MHz, $CDCl_3$): *δ* 8.05–8.02 (m, 4 H, Ar*H*), 7.55–7.52 (m, 2 H, Ar*H*), 7.42–7.39 (m, 4 H, Ar*H*), 5.73 (dd, *J* = 10.5, 3.5 Hz, 1 H, H-2), 5.63 (dd, *J* = 11.0, 2.5 Hz, 1 H, H-3), 5.31 (d, *J* = 3.5 Hz, 1 H, H-1), 4.90 (d, *J* = 2.0 Hz, 1 H, H-4), 4.37 (d, *J* = 12.5 Hz, 1 H, H-6), 4.26 (d, *J* = 12.5 Hz, 1 H, H-6), 3.95 (s, 1 H, H-5), 3.76–3.72 (m, 1 H, O$CH_2$$CH_2$), 3.48–3.43 (m, 1 H, O$CH_2$$CH_2$), 3.23 (t, *J* = 7.0 Hz, 2 H, CH_2N_3), 1.56–1.51 (m, 4 H, $OCH_2(CH_2)_6$), 1.31–1.19 (m, 8 H, $OCH_2(CH_2)_6$), 1.15 (s, 9 H, $SiC(CH_3)_3$), 0.99 (s, 9 H, $SiC(CH_3)_3$); ^{13}C NMR (125 MHz, $CDCl_3$): δ 166.2 (*C*OPh), 166.1 (*C*OPh), 133.1 (Ar*C*), 133.0 (Ar*C*), 129.9 (Ar*C*), 129.7 (Ar*C*), 129.68 (Ar*C*), 129.63 (Ar*C*), 128.3 (Ar*C*), 96.6 (C-1), 71.2 (C-3), 71.1 (C-4), 68.8 (C-2), 68.3 (OCH_2CH_2), 67.0 (C-6), 66.9 (C-5), 51.4 (CH_2N_3), 29.3 ($OCH_2(CH_2)_6$), 29.1 ($OCH_2(CH_2)_6$), 28.9 ($OCH_2(CH_2)_6$), 28.7 ($OCH_2(CH_2)_6$), 27.5 ($SiC(CH_3)_3$), 27.4 ($C(CH_3)_3$, 27.2 ($SiC(CH_3)_3$), 26.5 ($OCH_2(CH_2)_6$), 25.9 ($OCH_2(CH_2)_6$); HRMS (ESI) *m/z* calcd for $C_{36}H_{51}N_3NaO_8Si$ $[M+Na]^+$ 704.3338; found: 704.3342.

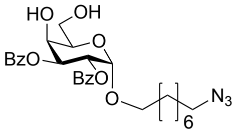

8-Azidooctyl 2,3-di-*O*-benzoyl-a-D-galactopyranoside (S3). To a solution of **S2** (220 mg, 0.323 mmol) anhydrous THF and pyridine (5 mL, 4:1) was added HF·pyridine (0.1 mL) at 0 °C. After stirring for 5 h at 0 °C, the reaction mixture was diluted with satd aq $NaHCO_3$ and extracted with EtOAc (5 × 50 mL). The combined organic layers were washed with brine, dried with Na_2SO_4, filtered and concentrated. The crude residue was purified by chromatography (20% to 50% EtOAc–Hexanes) to afford **S3** (122 mg, 70%) as an oil. $[\alpha]_D^{23}$ +52.2 (*c* = 0.75, CH_2Cl_2); ^{1}H NMR (500 MHz, $CDCl_3$): *δ* 8.03–8.00 (m, 4 H, Ar*H*), 7.56–7.52 (m, 2 H, Ar*H*), 7.42–7.38 (m, 4 H, Ar*H*), 5.70–5.64 (m, 2 H. H-2, H-3), 5.30 (d, *J* = 3.5 Hz, 1 H, H-1), 4.47 (dd, *J* = 3.0, 1.0 Hz, 1 H, H-4), 4.10 (app t, *J* = 4.5 Hz, 1 H, H-5), 4.01 (dd, *J* = 11.5, 5.0 Hz, 1 H, H-6), 3.93 (dd, *J* = 12.0, 4.0 Hz, 1 H, H-6), 3.80-3.75 (m, 1H, O$CH_2$$CH_2$), 3.43 (m, 1H, O$CH_2$$CH_2$), 3.24 (t, *J* = 7.0 Hz, 2 H, CH_2N_3), 1.56–

1.47 (m, 4 H, $OCH_2(CH_2)_6$), 1.31–1.11 (m, 8 H, $OCH_2(CH_2)_6$); ^{13}C NMR (125 MHz, $CDCl_3$): δ 166.0 (*C*OPh), 165.8 (*C*OPh), 133.3 (Ar*C*), 133.2 (Ar*C*), 129.8 (Ar*C*), 129.49 (Ar*C*), 129.40 (Ar*C*), 128.4 (Ar*C*), 128.3 (Ar*C*), 96.5 (C-1), 71.1 (C-3), 69.8 (C-4), 68.9 (2C, C-2, C-5), 68.5(OCH_2CH_2), 63.3 (C-6), 51.4 (CH_2N_3), 29.3 ($OCH_2(CH_2)_6$), 29.1 ($OCH_2(CH_2)_6$), 29.0 ($OCH_2(CH_2)_6$), 28.7 ($OCH_2(CH_2)_6$), 26.6 ($OCH_2(CH_2)_6$), 26.0 ($OCH_2(CH_2)_6$); HRMS (ESI) *m/z* calcd for $C_{28}H_{35}N_3NaO_8$ $[M+Na]^+$ 564.2316; found: 564.2321.

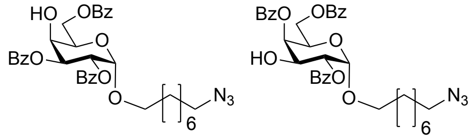

8-Azidooctyl 2,3,6-tri-*O*-benzoyl-a-D-galactopyranoside (S4) and **8-Azidooctyl 2,4,6-tri-*O*-benzoyl-a-D-galactopyranoside (S5).** A solution of thioglycoside **S3** (122 mg, 0.225 mmol) in CH_2Cl_2 (5 mL) was cooled to 0 °C and then triethylamine (0.4 ml, 2.87 mmol) was added dropwise until the solution became clear. After stirring for 5 min at 0 °C, benzoic anhydride (80 mg, 0.354 mmol) was added and the reaction mixture was stirred for 48 h while warming to room temperature. The reaction mixture was concentrated and toluene was added and evaporated twice. The crude residue was purified by chromatography (15% to 25% EtOAc–Hexane) to afford **S4** (82 mg, 56%) and **S5** (16 mg, 11%), both as oils. Data for **S4**: $[\alpha]_D^{23}$ +89.2 (c = 1.4, CH_2Cl_2); ^1H NMR (500 MHz, $CDCl_3$): δ 8.11–8.00 (m, 6 H, Ar*H*), 7.63–7.38 (m, 9 H, Ar*H*), 5.79 (dd, J = 11.0, 2.5 Hz, 1 H, H-3), 5.69 (dd, J = 10.5, 4.0 Hz, 1 H, H-2), 5.33 (d, J = 3.5 Hz, 1 H, H-1), 4.72 (dd, J = 11.0, 6.0 Hz, 1 H, H-6), 4.58 (dd, J = 11.5, 6.5 Hz, 1 H, H-6), 4.44–4.40 (m, 2H, H-4, H-5), 3.80–3.73 (m, 1 H, $OCH_2(CH_2)_6$), 3.49–3.45 (m, 1 H, $OCH_2(CH_2)_6$), 3.23 (t, J = 6.5 Hz, 2 H, CH_2N_3), 2.48 (s, 1 H, OH), 1.58–1.50 (m, 4 H, $OCH_2(CH_2)_6$), 1.29–1.17 (m, 8 H, $OCH_2(CH_2)_6$); ^{13}C NMR (125 MHz, $CDCl_3$): δ 166.4 (*C*OPh), 166.0 (*C*OPh), 165.8 (*C*OPh), 133.4 (Ar*C*), 133.3 (Ar*C*), 133.2 (Ar*C*), 129.8 (Ar*C*), 129.75 (Ar*C*), 129.71 (Ar*C*), 128.5 (Ar*C*), 128.47 (Ar*C*), 128.43 (Ar*C*), 128.3 (Ar*C*), 96.4 (C-1), 70.9 (C-3), 68.9 (C-2), 68.5 ($OCH_2(CH_2)_6$), 68.2 (C-5), 67.7 (C-4), 63.3 (C-6), 51.4 (CH_2N_3), 29.3 ($OCH_2(CH_2)_6$), 29.1 ($OCH_2(CH_2)_6$), 29.0 ($OCH_2(CH_2)_6$), 28.8 ($OCH_2(CH_2)_6$), 26.5 ($OCH_2(CH_2)_6$), 26.0 ($OCH_2(CH_2)_6$); HRMS (ESI) *m/z* calcd for $C_{35}H_{39}N_3NaO_9$ $[M+Na]^+$ 668.2579; found: 668.2582. Data for **S5**: $[\alpha]_D^{23}$ +87.7 (c = 1.4, CH_2Cl_2); ^1H NMR (500 MHz, $CDCl_3$): δ 8.17–

8.04 (m, 6 H, Ar*H*), 7.63–7.56 (m, 3 H, Ar*H*), 7.52–7.43 (m, 6 H, Ar*H*), 5.85 (d, *J* = 3.0 Hz, 1 H, H-4), 5.41 (dd, *J* = 10.5, 3.5 Hz, 1 H, H-2), 5.33 (d, *J* = 3.0 Hz, 1 H, H-1), 4.59-4.49 (m, 3 H, H-3, H-5, H-6), 4.46-4.43 (m, 1H, H-6'), 3.78–3.73 (m, 1 H $OCH_2(CH_2)_6$), 3.51–3.46 (m, 1 H, $OCH_2(CH_2)_6$), 3.24 (t, *J* = 7.0 Hz, 2 H, CH_2N_3), 2.35 (d, *J* = 5.5 Hz, 1H, OH), 1.60–1.52 (m, 4 H, $OCH_2(CH_2)_6$), 1.30–1.21 (m, 8 H, $OCH_2(CH_2)_6$); ^{13}C NMR (125 MHz, $CDCl_3$): δ 166.7 (*C*OPh), 166.4 (*C*OPh), 166.0 (*C*OPh), 133.5 (Ar*C*), 133.4 (Ar*C*), 133.1 (Ar*C*), 130.0 (Ar*C*), 129.8 (Ar*C*), 129.7 (Ar*C*), 129.5 (Ar*C*), 129.2 (Ar*C*), 128.6 (Ar*C*), 128.47 (Ar*C*), 128.43 (Ar*C*), 96.5 (C-1), 72.3 (C-2), 71.5 (C-4), 68.5 ($OCH_2(CH_2)_6$), 68.2 (C-5), 67.7 (C-3), 63.0 (C-6), 51.4 (CH_2N_3), 29.3 ($OCH_2(CH_2)_6$), 29.1 ($OCH_2(CH_2)_6$), 29.0 ($OCH_2(CH_2)_6$), 28.8 ($OCH_2(CH_2)_6$), 26.6 ($OCH_2(CH_2)_6$), 26.0 ($OCH_2(CH_2)_6$); HRMS (ESI) *m/z* calcd for $C_{35}H_{39}N_3NaO_9$ $[M+Na]^+$ 668.2579; found: 668.2581.

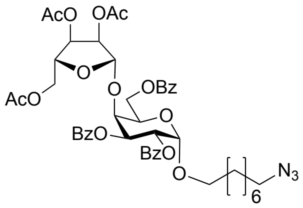

8-Azidooctyl 2,3,5-tri-*O*-acetyl-b-D-ribofuranosyl-(1→4)-2,3,6-tri-*O*-benzoyl-a-D-galactopyranoside (S7). Thioglycoside **S6** (12) (110 mg, 0.288 mmol) and acceptor **S4** (60 mg, 0.093 mmol) were dried overnight under vacuum and then dissolved in CH_2Cl_2 (5 mL). After stirring for 10 min at room temperature over 4 Å molecular sieves (freshly activated), the solution was cooled to 0 °C and stirred for 5 min. To the cooled reaction mixture were sequentially added *N*-iodosuccinimide (78 mg, 0.355 mmol) and TfOH (1.00 mL, 0.009 mmol) and the solution was stirred for 2 h at 0 °C. The reaction mixture was diluted with CH_2Cl_2 (100 mL) and filtered through Celite. The organic layer was successively washed with 5% $Na_2S_2O_3$ (100 mL) and satd aq $NaHCO_3$, dried with Na_2SO_4, filtered and concentrated to give a residue that was purified by chromatography (10% to 20% to 40% EtOAc–Hexane) to afford **S7** (66 mg, 79%) as an amorphous solid: $[\alpha]_D^{23}$ +52.9 (*c* = 1.0, CH_2Cl_2); ^1H NMR (500 MHz, $CDCl_3$): δ 8.06–7.95 (m, 6 H, Ar*H*), 7.59–7.34 (m, 9 H, Ar*H*), 5.81 (dd, *J* = 10.5, 3.5 Hz, 1 H, H-3), 5.56 (dd, *J* = 11.0, 3.5 Hz, 1H, H-2), 5.41 (m, 1 H, H-2'), 5.71 (app t, *J* = 5.5 Hz, 1 H, H-3'), 5.31 (d, *J* = 3.4 Hz, 1 H,

H-1), 5.17 (d, J = 1.5 Hz, 1H, H-1'), 4.58–4.55 (m, 1H, H-6), 4.51–4.47 (m, 1 H, H-6), 4.46 (s, 1 H, H-4), 4.40–4.37 (m, 1 H, H-5'), 4.26–4.13 (m, 3 H, H-4', H-5', H-5), 3.73–3.68 (m, 1 H, $OCH_2(CH_2)_6$), 3.44–3.40 (m, 1 H, $OCH_2(CH_2)_6$), 3.20 (t, J = 7.0 Hz, 2 H, CH_2N_3), 2.07 (s, 3 H, $COCH_3$), 1.97 (s, 3 H, $COCH_3$), 1.87 (s, 3 H, $COCH_3$), 1.55–1.47 (m, 4 H, $OCH_2(CH_2)_6$), 1.22–1.12 (m, 8 H, $OCH_2(CH_2)_6$); ^{13}C NMR (125 MHz, $CDCl_3$): δ 170.6 (*C*OPh/CH_3), 169.3 (*C*OPh/CH_3), 169.0 (*C*OPh/CH_3), 166.0 (*C*OPh/CH_3), 165.7 (*C*OPh/CH_3), 165.6 (*C*OPh/CH_3), 133.7 (Ar*C*), 133.3 (Ar*C*), 133.1 (Ar*C*), 133.0 (Ar*C*), 130.0 (Ar*C*), 129.9 (Ar*C*), 129.8 (Ar*C*), 129.7 (Ar*C*), 129.6 (Ar*C*), 129.4 (Ar*C*), 129.1 (Ar*C*), 128.4 (Ar*C*), 128.3 (Ar*C*), 128.3 (Ar*C*), 106.7 (C-1'), 96.2 (C-1), 78.6 (C-4'), 75.5 (C-4), 74.9 (C-2'), 70.6 (C 3'), 70.5 (C-3), 69.2 (C-2), 68.4 ($OCH_2(CH_2)_6$), 67.3 (C-5), 63.5 (2C, C-5', C-6), 51.3 (CH_2N_3), 29.2 ($OCH_2(CH_2)_6$), 29.0 ($OCH_2(CH_2)_6$), 28.9 ($OCH_2(CH_2)_6$), 28.7 ($OCH_2(CH_2)_6$), 26.5 ($OCH_2(CH_2)_6$), 25.9 ($OCH_2(CH_2)_6$), 20.7 ($COCH_3$), 20.3 ($COCH_3$), 20.2 ($COCH_3$); HRMS (ESI) *m/z* calcd for $C_{46}H_{53}N_3NaO_{16}$ $[M+Na]^+$ 926.3321; found: 926.3318.

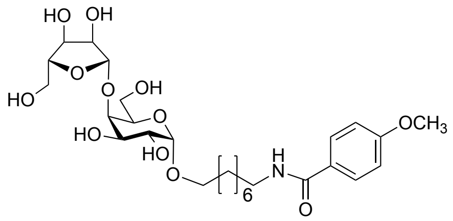

8-(*p*-Methoxy-benzamido)octyl b-D-ribofuranosyl-(1→4)-a-D-galactopyranoside (1). A solution of **S7** (50 mg, 0.055 mmol) in 0.1M CH_3ONa in CH_3OH (3 mL) was stirred at room temperature for 3 h. The reaction mixture was neutralized with few drops of acetic acid and concentrated to give a crude product that was purified by chromatography (15% to 50% CH_3OH–CH_2Cl_2) to afford a colorless oil that was used directly in the next step. A solution of the compound from the previous step (26 mg, 0.056 mmol) in CH_3OH (1 mL) was added to a suspension of palladium hydroxide on carbon (4 mg, 0.028 mmol, 20% wt). After purging the flask with H_2 gas three times, the reaction mixture was stirred under H_2 for 15 h at room temperature. The reaction mixture was diluted with CH_3OH, filtered through Celite, the Celite was washed with CH_3OH and the filtrate was concentrated. The resulting crude residue was dried under vacuum for 3 h and used directly for the next step

without further purification. A solution of the compound from the previous step (24 mg, 0.055 mmol) in THF (1 mL) was cooled to 0 °C and after stirring for 5 min, *p*-anisoyl chloride (14 mg, 0.083 mmol) was added and the mixture was stirred for 12 h. The reaction mixture was concentrated and toluene was added and evaporated twice. The crude residue was purified by chromatography (5% to 20% to 50% $CH_3OH–CH_2Cl_2$) to afford **1** (20 mg, 63% over three steps) as an amorphous solid: $[\alpha]_D^{23}$ = +10.5 (*c* = 0.50, CH_3OH); ^1H NMR (500 MHz, CD_3OD): *δ* 7.79–7.76 (m, 2 H, Ar*H*), 6.98–6.94 (m, 2 H, Ar*H*), 5.33 (s, 1 H, H-1’), 4.76 (d, *J* = 3.5 Hz, 1 H, H-1), 4.06–4.02 (m, 3 H, H-2’, H-3’, H-4), 3.95–3.92 (m, 1 H, H-4’), 3.85–3.78 (m, 5 H, H-3, OCH_3, H-5), 3.76–3.71 (m, 2 H, H-6, H-2), 3.70–3.65 (m, 2 H, $OCH_2(CH_2)_6$, H-5’), 3.63–3.54 (m, 2 H, H-6, H-5’), 3.45–3.40 (m, 1 H, $OCH_2(CH_2)_6$), 3.34 (t, *J* = 7.5 Hz, 2 H, CH_2NH), 1.65–1.57 (m, 4 H, $OCH_2(CH_2)_6$), 1.41–1.27 (m, 8 H, $OCH_2(CH_2)_6$); ^{13}C NMR (125 MHz, $CDCl_3$): *δ* 163.8 ($CONHCH_2$), 130.0 (Ar*C*), 127.9 (Ar*C*), 114.7 (Ar*C*), 110.1 (C-1’), 100.4 (C-1), 84.2 (C-4’), 76.7 (C-4), 76.5 (C-2’), 71.9 (C-3’), 71.89 (C-5), 71.80 (C-3), 70.4 (C-2), 69.3 ($OCH_2(CH_2)_6$), 64.0 (C-6), 61.8 (C-5’), 55.9 (OCH_3), 40.9 (CH_2NH), 30.5 (2C, $OCH_2(CH_2)_6$), 30.4 ($OCH_2(CH_2)_6$), 30.3 ($OCH_2(CH_2)_6$), 28.0 ($OCH_2(CH_2)_6$), 27.2 ($OCH_2(CH_2)_6$); HRMS (ESI) *m/z* calcd for $C_{27}H_{43}NNaO_{12}$ $[M+Na]^+$ 596.2677; found: 596.2681.

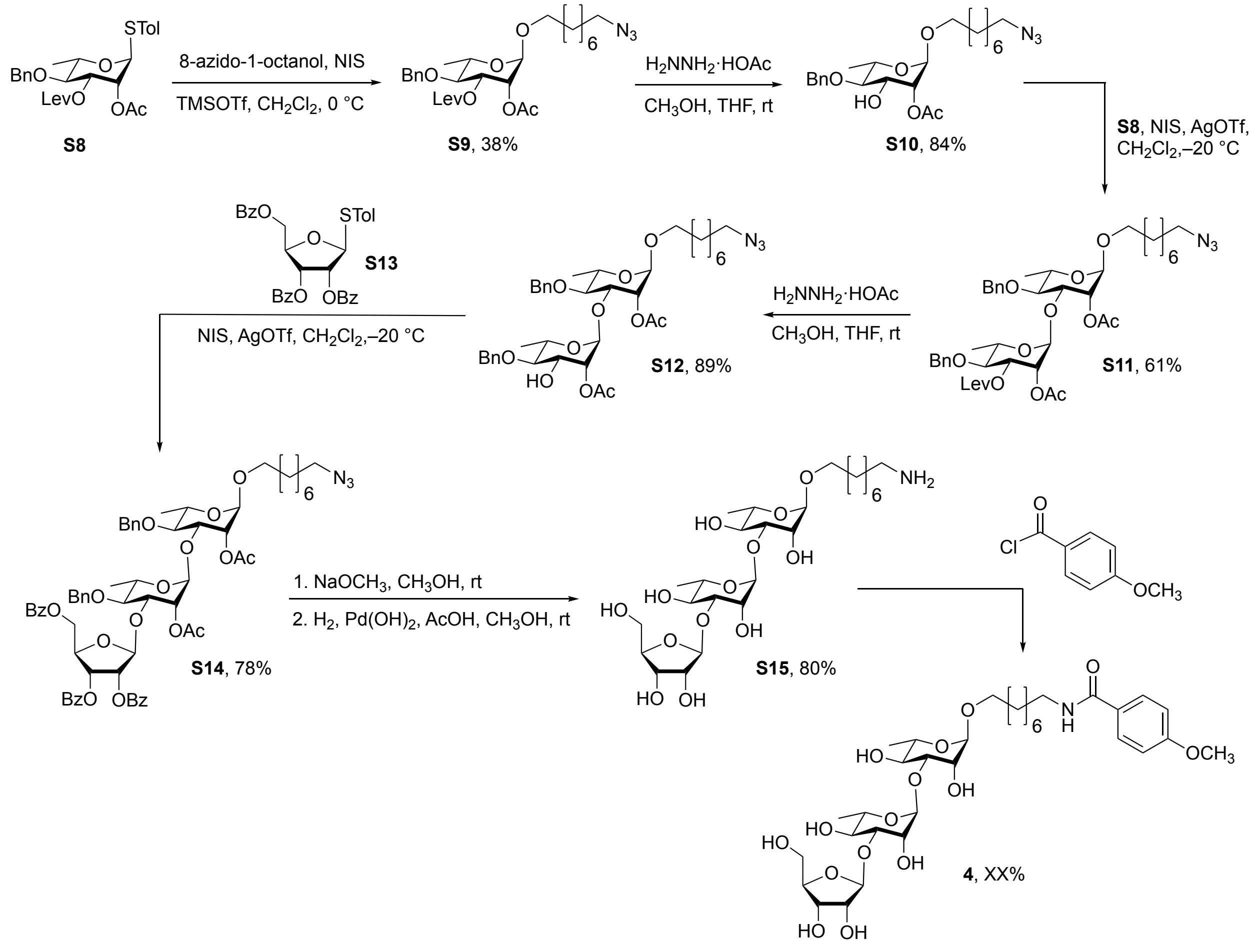

SI Scheme 2. Synthesis of **4**

Procedures and Data

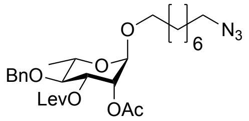

8-Azidooctyl 2-*O*-acetyl-4-*O*-benzyl-3-*O*-levulinoyl-α-L-rhamnopyranoside (S9). A mixture of **S8** (0.12 g, 0.20 mmol, 1.0 equiv) and 8-azidooctanol (11) (0.041 g, 0.24 mmol, 1.0 equiv) was dried under vacuum for 30 min. The mixture was dissolved in dry CH_2Cl_2 (3 mL) and then 4 Å molecular sieves (100 mg) were added. Then, *N*-iodosuccinimide (0.041 g, 1.4 equiv) and trimethylsilyl trifluoromethanesulfonate (8.0 µL, 0.15 equiv) were added at 0 °C. The mixture was stirred at 0 °C for 1 h, then Et_3N was added until the solution pH was 7. The reaction mixture was filtered through Celite and then washed with a satd aq soln of $Na_2S_2O_3$ (2 × 10 mL). The organic layer was dried with Na_2SO_4, filtered, and the filtrate was concentrated. The resulting crude residue was purified by chromatography (6:1 hexane–EtOAc) to give **S9** (0.046 g, 38%) as a yellow oil: R_f = 0.23 (3:1 hexane–EtOAc); $[\alpha]_D^{20}$ –29.4 (*c* 0.17, $CHCl_3$); ^{1}H NMR (500 MHz, $CDCl_3$) *δ* 7.36–7.27 (m, 5 H, Ar*H*), 5.31 (dd, *J* = 10.0, 3.5 Hz, 1 H, H-3), 5.22 (dd, *J* = 3.5, 2.0 Hz, 1 H, H-2), 4.73 and 4.63 (ABq, J_{AB} = 11.0 Hz, 2 H, C*H*$_2$Ar), 4.66 (d, *J* = 1.5 Hz, 1 H, H-1), 3.75 (dq, *J* = 9.5, 6.0 Hz, 1 H, H-5), 3.63 (ddd, *J* = 9.5, 6.5, 6.5 Hz, 1 H, OC*H*$_2$CH$_2$), 3.50 (dd, *J* = 9.5, 9.5 Hz, 1 H, H-4), 3.38 (ddd, *J* = 9.5, 6.5, 6.5 Hz, 1 H, OC*H*$_2$CH$_2$), 3.25 (t, *J* = 7.0 Hz, 2 H, C*H*$_2$N$_3$), 2.78–2.63 (m, 2 H, CH$_2$ Lev), 2.55–2.43 (m, 2 H, CH$_2$ Lev), 2.16 (s, 3 H, CH$_3$ Lev), 2.14 (s, 3 H, CH$_3$ Ac), 1.62–1.53 (m, 10 H, OCH$_2$(C*H*$_2$)$_6$), 1.37–1.31 (m, 5 H, OCH$_2$(C*H*$_2$)$_6$, H-6); ^{13}C NMR (126 MHz, $CDCl_3$) *δ* 206.3 (C=O Lev), 171.8 (C=O Lev), 170.3 (*C*OCH$_3$), 138.1 (Ar*C*), 128.5 (Ar*C*), 127.9 (Ar*C*), 97.4 (C-1), 78.9 (C-4), 75.1 (*C*H$_2$Ar), 72.3 (C-3), 70.5 (C-2), 68.0 (O*C*H$_2$CH$_2$), 67.6 (C-5), 51.5 (CH$_2$N$_3$), 37.9 (CH$_2$ Lev), 29.9 (CH$_3$ Lev), 29.4 (OCH$_2$(*C*H$_2$)$_6$), 29.3 (OCH$_2$(*C*H$_2$)$_6$), 29.1 (OCH$_2$(*C*H$_2$)$_6$), 28.9 (OCH$_2$(*C*H$_2$)$_6$), 28.0 (CH$_2$ Lev), 26.7 (OCH$_2$(*C*H$_2$)$_6$) 26.0 (OCH$_2$(*C*H$_2$)$_6$), 21.0 (CH$_3$ Ac), 18.0 (C-6); HRMS (ESI) *m/z* Calcd for $C_{28}H_{41}NaN_3O_8$ $[M + Na]^+$ 570.2786, found 570.2780.

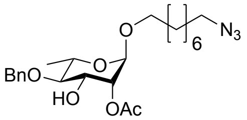

8-Azidooctyl 2-*O*-acetyl-4-*O*-benzyl-α-L-rhamnopyranoside (S10). Compound **S9** (0.18 g, 0.35 mmol, 1.0 equiv.) was dissolved in THF (1.8 mL) and then NH_2NH_2–AcOH (0.064 g, 2.0 equiv) in CH_3OH (0.2 mL) was added at room temperature. The mixture was stirred at room temperature for 45 min and then the mixture was washed with a satd aq $NaHCO_3$ soln (3 × 10 mL). The organic layer was dried with Na_2SO_4, filtered, and the filtrate was concentrated. The resulting crude residue was purified by chromatography (6:1 hexane–EtOAc) to give **S10** (0.13 g, 84%) as yellow oil: R_f = 0.44 (7:3 hexane–EtOAc); $[\alpha]_D^{20}$ –41.4 (*c* 0.14, $CHCl_3$); ^1H NMR (500 MHz, $CDCl_3$) δ 7.38–7.29 (m, 5 H, Ar*H*), 5.09 (dd, *J* = 4.0, 2.0 Hz, 1 H, H-2), 4.83 (ABq, J_{AB} = 11.0 Hz, 1 H, CH_2Ar), 4.73–4.71 (m, 2 H, CH_2Ar, H-1), 4.11 (d, *J* = 9.0 Hz, H-3), 3.74 (app dq, *J* = 9.5, 6.5 Hz, 1 H, H-5), 3.63 (ddd, *J* = 9.5, 6.5, 6.5 Hz, 1 H, OCH_2CH_2), 3.40–3.33 (m, 2 H, OCH_2CH_2, H-4), 3.25 (t, *J* = 7.0 Hz, 2 H, CH_2N_3), 2.16 (s, 3 H, CH_3 Ac), 1.62–1.52 (m, 4 H, $OCH_2(CH_2)_6$), 1.35 (d, *J* = 6.0 Hz, 3 H, H-6), 1.34–1.25 (m, 8 H, $OCH_2(CH_2)_6$); ^{13}C NMR (126 MHz, $CDCl_3$) δ 170.9 (*C*OCH_3), 138.3 (Ar*C*), 128.5 (Ar*C*), 127.9 (Ar*C*), 97.3 (C-1), 81.8 (C-4), 75.2 (*C*H_2Ar), 73.0 (C-2), 70.3 (C-3), 67.8 (O*C*H_2CH_2), 67.3 (C-5), 51.4 (CH_2N_3), 29.3 ($OCH_2(CH_2)_6$), 29.2 ($OCH_2(CH_2)_6$), 29.0 ($OCH_2(CH_2)_6$), 28.8 ($OCH_2(CH_2)_6$), 26.6 ($OCH_2(CH_2)_6$), 26.0 ($OCH_2(CH_2)_6$), 21.1 (CH_3 Ac), 18.0 (C-6); HRMS (ESI) *m/z* Calcd for $C_{23}H_{35}NaN_3O_6$ $[M + Na]^+$ 472.2418, found 472.2413.

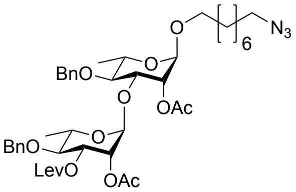

8-Azidooctyl 2-*O*-acetyl-4-*O*-benzyl-3-*O*-levulinoyl-α-L-rhamnopyranosyl-(1→3)-2-*O*-acetyl-4-*O*-benzyl-α-L-rhamnopyranoside (S11). A mixture of **S10** (0.016 g, 0.036 mmol, 1.0 equiv) and compound **S8** (0.020 g, 0.040 mmol, 1.1 equiv) was dried under vacuum for 30 min. The mixture was dissolved in dry CH_2Cl_2 (1 mL) and then 4 Å molecular sieves (100 mg) were added. The solution was cooled to –20 °C and then *N*-

iodosuccinimide (0.011 g, 1.4 equiv) and AgOTf (0.0029 g, 0.2 equiv) were added. The mixture was stirred at –20 °C for 10 min, then Et_3N was added until the solution pH was 7. The reaction mixture was filtered through Celite, then washed with a satd aq soln of $Na_2S_2O_3$ (2 × 10 mL). The organic layer was dried with Na_2SO_4, filtered, and the filtrate was concentrated. The crude residue was purified by chromatography (6:1 hexane–EtOAc) to give **S11** (0.018 g, 61%) as a colorless oil: R_f = 0.20 (3:1 hexane–EtOAc); $[\alpha]_D^{20}$ –32.4 (*c* 0.60, $CHCl_3$); ^1H NMR (500 MHz, $CDCl_3$) *δ* 7.69–7.27 (m, 10 H, Ar*H*), 5.34–5.28 (m, 2 H, H-2', H-3'), 5.10 (d, *J* = 3.5 Hz, 1 H, H-2), 4.97 (s, 1 H, H-1'), 4.85 (ABq, J_{AB} = 10.5 Hz, 1 H, CH_2Ar), 4.72 (ABq, J_{AB} = 11.5 Hz, 1 H, CH_2Ar), 4.68 (s, 1 H, H-1), 4.64–4.61 (m, 2 H, 2 × CH_2Ar), 4.08 (dd, *J* = 9.5, 3.5 Hz, 1 H, H-3), 3.85 (dq, *J* = 9.5, 6.0 Hz, 1 H, H-5'), 3.71 (dq, *J* = 9.5, 6.0 Hz, 1 H, H-5), 3.60 (ddd, *J* = 9.5, 6.5, 6.5 Hz, 1 H, OCH_2CH_2), 3.50–3.44 (m, 2 H, H-4, H-4'), 3.36 (ddd, *J* = 9.5, 6.5, 6.5 Hz, 1 H, OCH_2CH_2), 3.24 (t, *J* = 7.0 Hz, 2 H, CH_2N_3), 2.76–2.62 (m, 2 H, CH_2 Lev), 2.54–2.42 (m, 2 H, CH_2 Lev), 2.16 (s, 3 H, CH_3 Ac), 2.15 (s, 3 H, CH_3 Lev), 2.07 (s, 3 H, CH_3 Ac), 1.62–1.50 (m, 6 H, $OCH_2(CH_2)_6$), 1.37–1.28 (m, 12 H, $OCH_2(CH_2)_6$, H-6, H-6'); ^{13}C NMR (126 MHz, $CDCl_3$) *δ* 206.3 (C=O Lev), 171.9 (C=O Lev), 170.6 ($COCH_3$), 169.9 ($COCH_3$), 138.2 (Ar*C*), 138.1 (Ar*C*), 128.4 (Ar*C*), 128.2 (Ar*C*), 128.17 (Ar*C*), 127.9 (Ar*C*), 127.8 (Ar*C*), 99.6 (C-1'), 97.0 (C-1), 80.4 (C-4), 78.4 (C-4'), 78.0 (C-3), 75.7 (CH_2Ar), 74.7 (CH_2Ar), 72.4 (C-2), 71.9 (C-3'), 70.3 (C-2'), 68.5 (C-5'), 67.9 (OCH_2CH_2), 67.7 (C-5), 51.5 (CH_2N_3), 37.9 (CH_2 Lev), 29.8 (CH_3 Lev), 29.4 ($OCH_2(CH_2)_6$), 29.3 ($OCH_2(CH_2)_6$), 29.1 ($OCH_2(CH_2)_6$), 28.9 ($OCH_2(CH_2)_6$), 28.0 (CH_2 Lev), 26.7 ($OCH_2(CH_2)_6$), 26.0 ($OCH_2(CH_2)_6$), 21.2 (CH_3 Ac), 20.9 (CH_3 Ac), 18.0 (C-6'), 17.9 (C-6); HRMS (ESI): *m/z* calcd for $C_{43}H_{59}NaN_3O_{13}$ $[M + Na]^+$ 848.3940, found 848.3936.

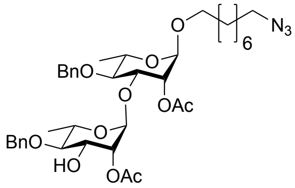

8-Azidooctyl 2-*O*-acetyl-4-*O*-benzyl-6-α-L-rhamnopyranosyl-(1 → 3)-2-*O*-acetyl-4-*O*-benzyl-6-α-L-rhamnopyranoside (S12). Compound **S11** (0.22 g, 0.27 mmol, 1.0 equiv) was dissolved in THF (2.7 mL), then NH_2NH_2-AcOH (0.050 g, 2.0 equiv) in CH_3OH (0.3

mL) was added at room temperature. The mixture was stirred at room temperature for 1 h, then extracted with $NaHCO_3$ (3 × 10 mL). The combined organic phase was dried with Na_2SO_4, filtered, and concentrated. The crude was purified by chromatography (1:1 hexane–EtOAc) to give **S12** (0.18 g, 89%) as a colorless oil: R_f = 0.85 (60% ethyl acetate/hexane); $[\alpha]_D^{20}$: –32.2 (*c* 3.58, $CHCl_3$); ^1H NMR (500 MHz, $CDCl_3$) *δ* 7.36–7.27 (m, 10 H, Ar*H*), 5.18 (d, *J* = 3.5 Hz, 1 H, H-2), 5.08 (d, *J* = 3.5 Hz, 1 H, H-2'), 4.97 (s, 1 H, H-1), 4.82 and 4.60 (ABq, J_{AB} = 11.0 Hz, 2 H, C*H*$_2$Ar), 4.77 and 4.73 (ABq, J_{AB} = 11.0 Hz, 2 H, C*H*$_2$Ar), 4.68 (s, 1 H, H-1'), 4.10 (dd, *J* = 9.5, 3.5 Hz, 1 H, H-3'), 4.04 (app d, *J* = 9.5 Hz, 1 H, H-3), 3.79–3.68 (m, 2 H, H-5, H-5'), 3.60 (ddd, *J* = 13.0, 3.0, 3.0 Hz, 1 H, OC*H*$_2$CH$_2$), 3.44 (dd, *J* = 9.5, 9.5 Hz, 1 H, H-4), 3.38–3.32 (m, 2 H, OCH$_2$CH$_2$, H-4'), 3.24 (t, *J* = 7.0 Hz, 2 H, C*H*$_2$N$_3$), 2.14 (s, 3 H, COC*H*$_3$), 2.10 (s, 3 H, COC*H*$_3$), 1.61–1.51 (m, 6 H, OCH$_2$(C*H*$_2$)$_6$), 1.37–1.28 (m, 12 H, OCH$_2$(C*H*$_2$)$_6$, H-6, H-6'); ^{13}C NMR (126 MHz, $CDCl_3$) *δ* 170.6 (*C*OCH$_3$), 170.4 (*C*OCH$_3$), 138.3 (Ar*C*), 138.0 (Ar*C*), 128.6 (Ar*C*), 128.5 (Ar*C*), 128.1 (Ar*C*), 128.0 (Ar*C*), 127.96 (Ar*C*), 127.8 (Ar*C*), 99.5 (C-1), 97.0 (C-1'), 81.5 (C-4'), 80.6 (C-4), 77.3 (C-3'), 75.6 (*C*H$_2$Ar), 74.9 (*C*H$_2$Ar), 72.7 (C-2), 72.5 (C-2'), 70.0 (C-3), 68.3 (C-5), 67.9 (O*C*H$_2$CH$_2$), 67.7 (C-5'), 51.5 (*C*H$_2$N$_3$), 29.4 (OCH$_2$(*C*H$_2$)$_6$), 29.3 (OCH$_2$(*C*H$_2$)$_6$), 29.1 (OCH$_2$(*C*H$_2$)$_6$), 28.9 (OCH$_2$(*C*H$_2$)$_6$), 26.7 (OCH$_2$(*C*H$_2$)$_6$), 26.0 (OCH$_2$(*C*H$_2$)$_6$), 21.2 (CO*C*H$_3$), 21.0 (CO*C*H$_3$), 18.0 (C-6), 17.9 (C-6'); HRMS (ESI): *m/z* calcd for $C_{38}H_{53}NaN_3O_{11}$ $[M + Na]^+$ 750.3572, found 750.3567.

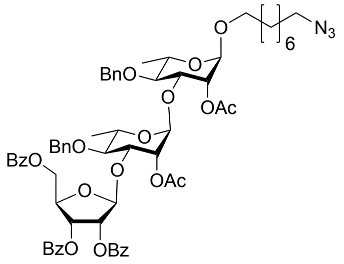

8-Azidooctyl 2,3,5-tri-*O*-benzoyl-β-D-ribofuranosyl-(1 → 3)-2-*O*-acetyl-4-*O*-benzyl-6-α-L-rhamnopyranosyl-(1 → 3)-2-*O*-acetyl-4-*O*-benzyl-6-α-L-rhamnopyranoside (S7). A mixture of **S12** (0.033 g, 0.045 mmol, 1.0 equiv) and compound **S13** (12) (0.028 g, 1.1 equiv) was dried under vacuum for 30 min. The mixture was dissolved in dry CH_2Cl_2 (1 mL), then 4 Å molecular sieves (80 mg) were added. Then, *N*-iodosuccinimide (0.014 g, 1.4 equiv) and AgOTf (0.0020 g, 0.2 equiv) were added at –20 °C under Ar. The mixture

was stirred at –20 °C for 10 min under Ar, and then Et_3N was added until the solution pH was 7. The reaction mixture was filtered with Celite, and then extracted with $Na_2S_2O_3$ (2 × 10 mL). The combined organic phase was dried with Na_2SO_4, filtered, and concentrated. The crude was purified by chromatography (6:1 hexane–EtOAc) to give **S14** (0.041 g, 78%) as a colorless oil: R_f = 0.49 (30% ethyl acetate/hexane); $[\alpha]_D^{20}$ –5.59 (*c* 0.79, $CHCl_3$); ^1H NMR (500 MHz, $CDCl_3$) δ 8.01–8.00 (m, 2 H, Ar*H*), 7.89–7.83 (m, 4 H, Ar*H*), 7.56–7.47 (m, 3 H, Ar*H*), 7.37–7.20 (m, 13 H, Ar*H*), 7.18–7.11 (m, 3 H, Ar*H*), 5.73 (dd, *J* = 4.5, 1.0 Hz, 1 H, H-2), 5.68 (dd, *J* = 5.0, 5.0 Hz, 1 H, H-3), 5.55 (d, *J* = 1.5 Hz, 1 H, H-1), 5.43 (dd, *J* = 3.0, 2.0 Hz, 1 H, H-2'), 5.10 (dd, *J* = 3.5, 2.0 Hz, 1 H, H-2''), 5.02 (d, *J* = 1.5 Hz, 1 H, H-1'), 4.88 (ABq, J_{AB} = 10.5 Hz, 1 H, CH_2Ar), 4.83 (ABq, J_{AB} = 11.0 Hz, 1 H, CH_2Ar), 4.68 (d, *J* = 1.5 Hz, 1 H, H-1''), 4.64–4.60 (m, 2 H, CH_2Ar, H-4), 4.58–4.52 (m, 4 H, CH_2Ar, C-5), 4.17 (dd, *J* = 9.0, 3.0 Hz, 1 H, H-3'), 4.12 (dd, *J* = 9.5, 3.0 Hz, 1 H, H-3''), 3.78 (dq, *J* = 9.5, 6.0 Hz, 1 H, H-5'), 3.71 (dq, *J* = 10.0, 6.5 Hz, 1 H, H-5''), 3.60 (ddd, *J* = 9.5, 7.0 Hz, 1 H, OCH_2CH_2), 3.50–3.41 (m, 2 H, H-4', H-4''), 3.35 (ddd, *J* = 9.5, 6.5 Hz, 1 H, OCH_2CH_2), 3.24 (t, *J* = 7.0 Hz, 2 H, CH_2N_3), 2.12 (s, 3 H, $COCH_3$), 2.09 (s, 3 H, $COCH_3$), 1.62–1.52 (m, 4 H, $OCH_2(CH_2)_6$), 1.35–1.24 (m, 14 H, $OCH_2(CH_2)_6$, H-6', H-6''); ^{13}C NMR (126 MHz, $CDCl_3$) δ 170.5 ($COCH_3$), 170.0 ($COCH_3$), 166.0 (*C*OPh), 165.3 (*C*OPh), 165.1 (*C*OPh), 138.0 (ArC), 133.5 (Ar*C*), 133.4 (Ar*C*), 133.1 (Ar*C*), 129.83 (Ar*C*), 129.80 (Ar*C*), 129.1 (Ar*C*), 129.0 (Ar*C*), 128.5 (Ar*C*), 128.4 (Ar*C*), 128.4 (Ar*C*), 128.3 (Ar*C*), 128.1 (Ar*C*), 128.0 (Ar*C*), 127.8 (Ar*C*), 127.7 (Ar*C*), 107.2 (C-1), 99.4 (C-1'), 97.0 (C-1''), 80.4 (C-4'), 80.3 (C-4''), 79.1 (C-4), 77.6 (C-3''), 77.5 (C-3'), 75.6 (CH_2Ar), 75.59 (C-2), 75.51 (CH_2Ar), 73.1 (C-3), 72.5 (C-2''), 72.4 (C-2'), 68.8 (C-5'), 67.9 (OCH_2CH_2), 67.6 (C-5''), 65.6 (C-5), 51.5 (CH_2N_3), 29.4 ($OCH_2(CH_2)_6$), 29.3 ($OCH_2(CH_2)_6$), 29.1 ($OCH_2(CH_2)_6$), 28.9 ($OCH_2(CH_2)_6$), 26.7 ($OCH_2(CH_2)_6$), 26.1 ($OCH_2(CH_2)_6$), 21.2 ($COCH_3$), 21.0 ($COCH_3$), 18.0 (C-6'), 17.9 (C-6''); HRMS (ESI): *m/z* calcd for $C_{64}H_{77}N_4O_{18}$ $[M + NH_4]^+$ 1189.5227, found 1189.5221.

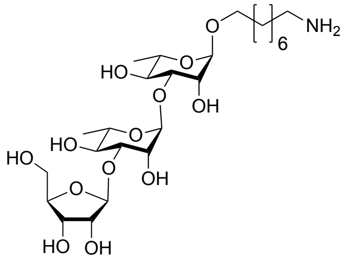

8-Aminooctyl β-D-ribofuranosyl-(1→3)-6-α-L-rhamnopyranosyl-(1→3)-6-α-L-rhamnopyranoside (S15). The compound **S14** (0.041 g, 0.035 mmol, 1.0 equiv) was dissolved in CH_3OH (1 mL), then 1 M $NaOCH_3$ (0.10 mL) was added at room temperature. The mixture was stirred at room temperature for 2 h, then Amberlite IR120 H+ form resin was added until the solution pH was 7. The mixture was filtered and concentrated. Then the mixture was dissolved in CH_3OH (4 mL) under Ar, then $Pd(OH)_2$ (0.028 g, 0.10 equiv) and AcOH (2 μL, 1 equiv) were added. The mixture was stirred for 5 min at room temperature under Ar, then under H_2 for 12 h. The mixture was filtered through Celite and then concentrated. The resulting oily residue was dissolved in H_2O (5 mL) and washed with CH_2Cl_2 (2 × 10 mL). The aqueous layer was separated, concentrated and lyophilized to give **S15** (0.016 g, 80%) as a yellowish foam: $[\alpha]_D^{20}$ –55.0 (*c* 0.02, CH_3OH); ^1H NMR (500 MHz, CD_3OD) δ 5.06 (d, *J* = 1.5 Hz, 1 H, H-1''), 5.01 (d, *J* = 1.5 Hz, 1 H, H-1'), 4.62 (d, *J* = 1.5 Hz, 1 H, H-1), 4.37 (dd, *J* = 3.0, 7.5 Hz, 1 H), 4.26 (d, *J* = 3.0 Hz, 1 H, H-2'), 4.05 (d, *J* = 3.5, 1 H, H-2''), 3.92–3.29 (m, 10 H), 2.89 (t, *J* = 7.5 Hz, 2 H, CH_2NH_2), 1.65–1.58 (m, 4 H, $OCH_2(CH_2)_6$), 1.42–1.33 (m, 6 H, $OCH_2(CH_2)_6$), 1.20–1.15 (m, 6 H, H-6', H-6''); ^{13}C NMR (126 MHz, CD_3OD) δ 110.4 (C-1''), 103.6 (C-1'), 101.6 (C-1), 84.4, 79.6, 79.6, 76.7 (C-2''), 73.2, 72.3, 72.2 (C-2'), 71.4, 70.7, 70.1, 69.9, 68.6, 61.9, 59.5, 41.0, 30.5 ($OCH_2(CH_2)_6$), 30.2 ($OCH_2(CH_2)_6$), 30.1 ($OCH_2(CH_2)_6$), 27.4 ($OCH_2(CH_2)_6$), 27.3 ($OCH_2(CH_2)_6$), 24.8 ($OCH_2(CH_2)_6$), 18.1, 18.0; HRMS (ESI): *m/z* calcd for $C_{25}H_{48}NO_{13}$ $[M + H]^+$ 570.3120, found 570.3120.

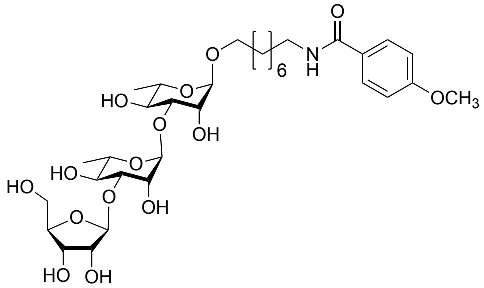

8-(*p*-Methoxy-benzamido)octyl β-D-ribofuranosyl-(1→3)-6-α-L-rhamnopyranosyl-(1→3)-6-α-L-rhamnopyranoside (4). To a solution of **S15** in THF–water (3:1, 1 mL) was added triethylamine (2 drops), and 4-methoxybenzoyl chloride (7.8 mg, 0.046 mmol, 3.0 equiv). The reaction mixture was stirred overnight and then concentrated. The resulting residue was purified by flash column chromatography (CH_2Cl_2–CH_3OH, 6:1) to yield **4** (8.2 mg, 94%) as a clear oil: R_f = 0.45 (CH_2Cl_2–CH_3OH, 4:1); $[\alpha]^{21}_D$ –28.0 (*c* 0.41, CH_3OH); ^1H NMR (500 MHz, CD_3OD) δ 7.77 (d, *J* = 8.9 Hz, 2 H, Ar*H*), 6.96 (d, *J* = 8.9 Hz, 2 H, Ar*H*), 5.06 (s, 1 H), 5.01 (d, *J* = 1.7 Hz, 1H), 4.62 (d, *J* = 1.8 Hz, 1 H), 4.37 (dd, *J* = 7.5, 4.6 Hz, 1 H), 4.26 (dd, *J* = 3.4, 1.7 Hz, 1 H), 4.04 (d, *J* = 4.6 Hz, 1 H), 3.91 (dt, *J* = 7.6, 3.0 Hz, 1 H), 3.86 (t, *J* = 3.3, 1.8 Hz, 1 H), 3.84 (s, 3 H, OCH_3), 3.83–3.37 (m, 10 H), 3.34 (d, *J* = 7.7 Hz, 2 H, CH_2N), 1.65–1.51 (m, 4 H, $OCH_2(CH_2)_6$), 1.44–1.33 (m, 8H, $OCH_2(CH_2)_6$), 1.26–1.21 (m, 6 H, H-6', H-6''); ^{13}C NMR (126 MHz, CD_3OD) δ 169.8 (*C*OAr), 130.1 (Ar*C*), 114.7 (Ar*C*), 110.5 (C-1''), 103.7 (C-1'), 101.6 (C-1), 84.4, 82.0, 79.6, 76.7, 73.3, 72.3, 72.2, 71.5, 70.8, 70.1, 70.0, 68.7, 62.0, 55.9, 41.0, 30.6 ($OCH_2(CH_2)_6$), 30.4 ($OCH_2(CH_2)_6$), 28.1 ($OCH_2(CH_2)_6$), 27.3 ($OCH_2(CH_2)_6$), 18.1, 18.0; HRMS (ESI): *m/z* calcd for $C_{33}H_{53}NaNO_{13}$ (M + Na) 726.3307, found 726.3300.

^{1}H NMR spectrum of compound **S2**

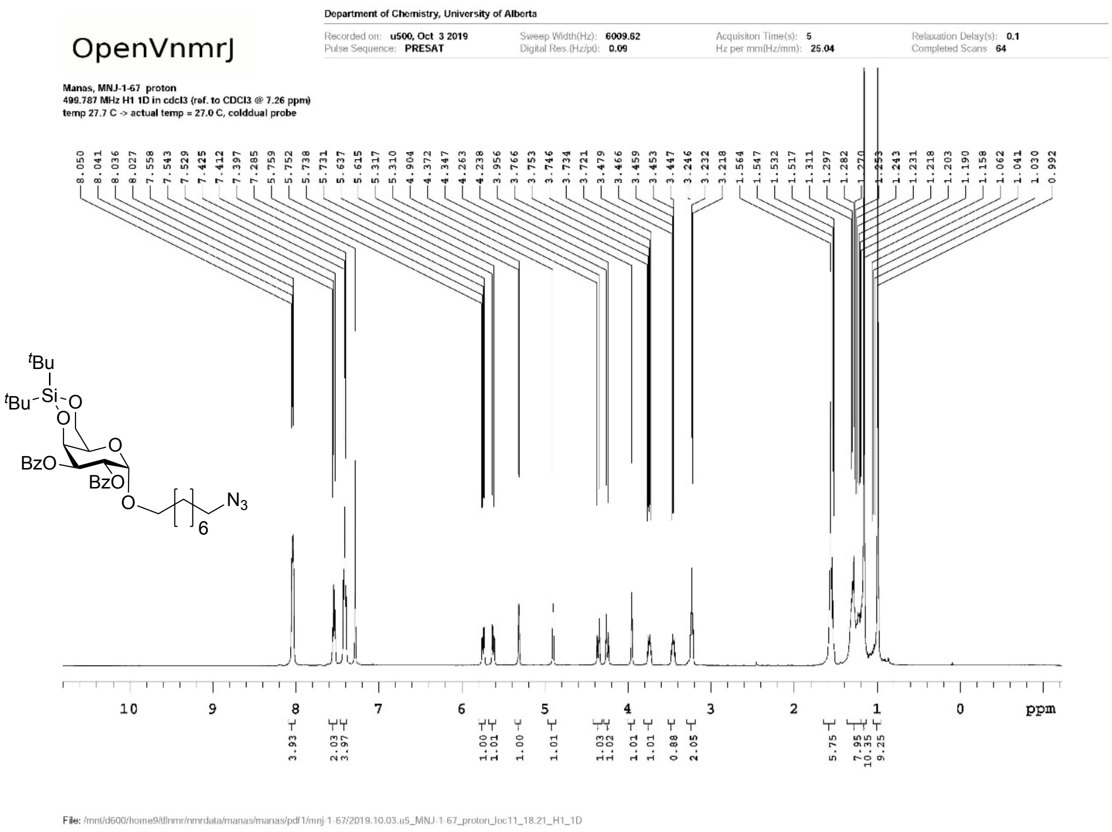

^{13}C NMR spectrum of compound **S2**

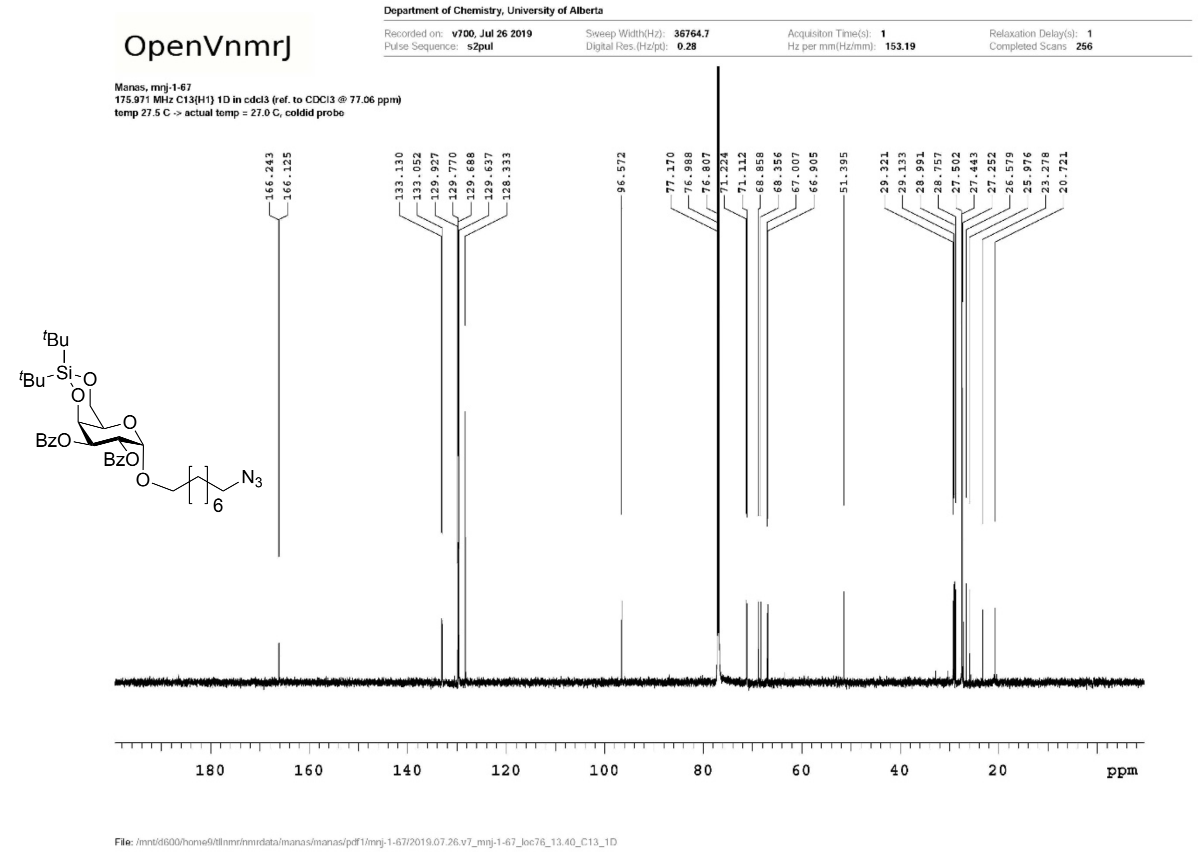

^{1}H NMR spectrum of compound **S3**

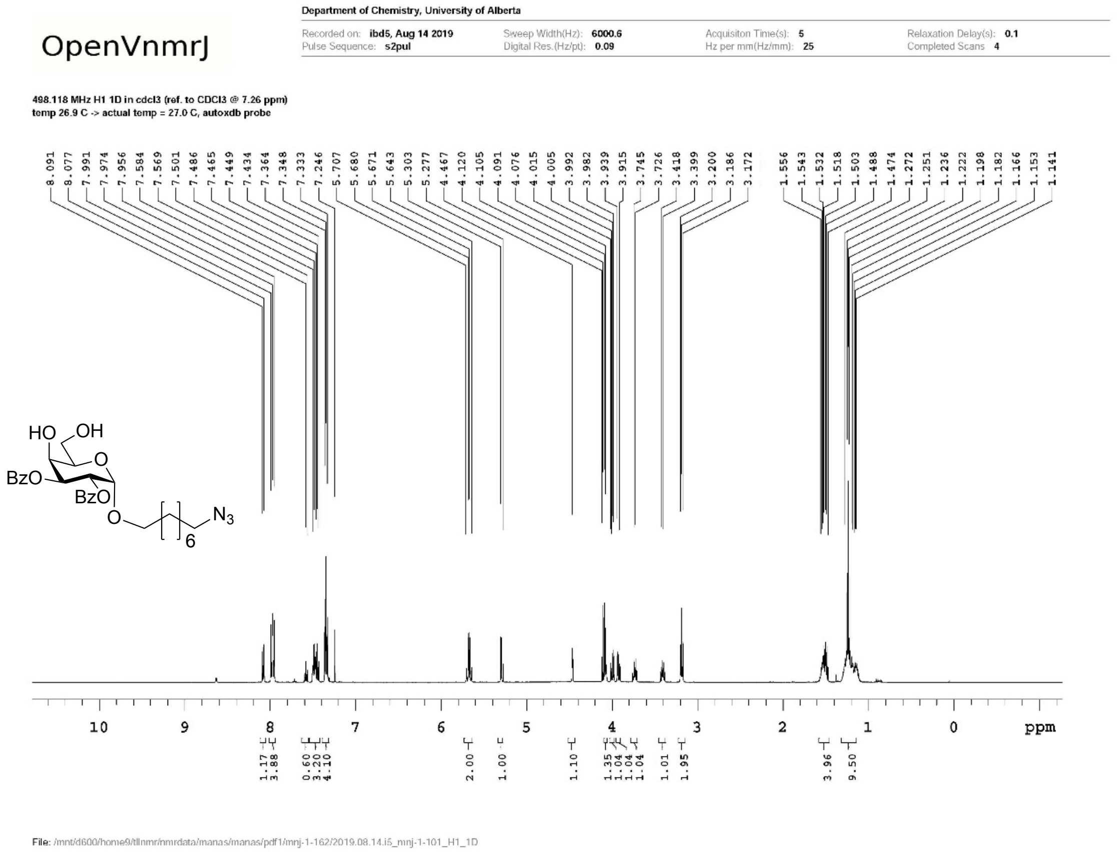

^{13}C NMR spectrum of compound **S3**

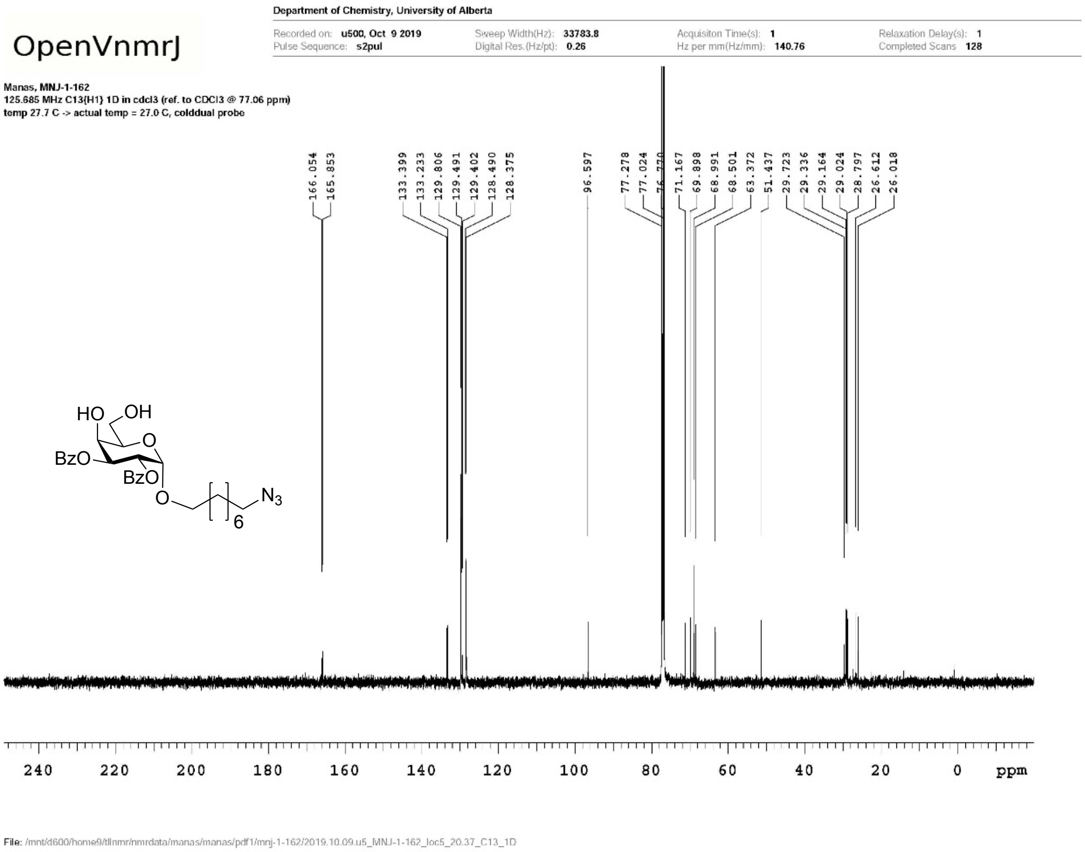

^{1}H NMR spectrum of compound **S4**

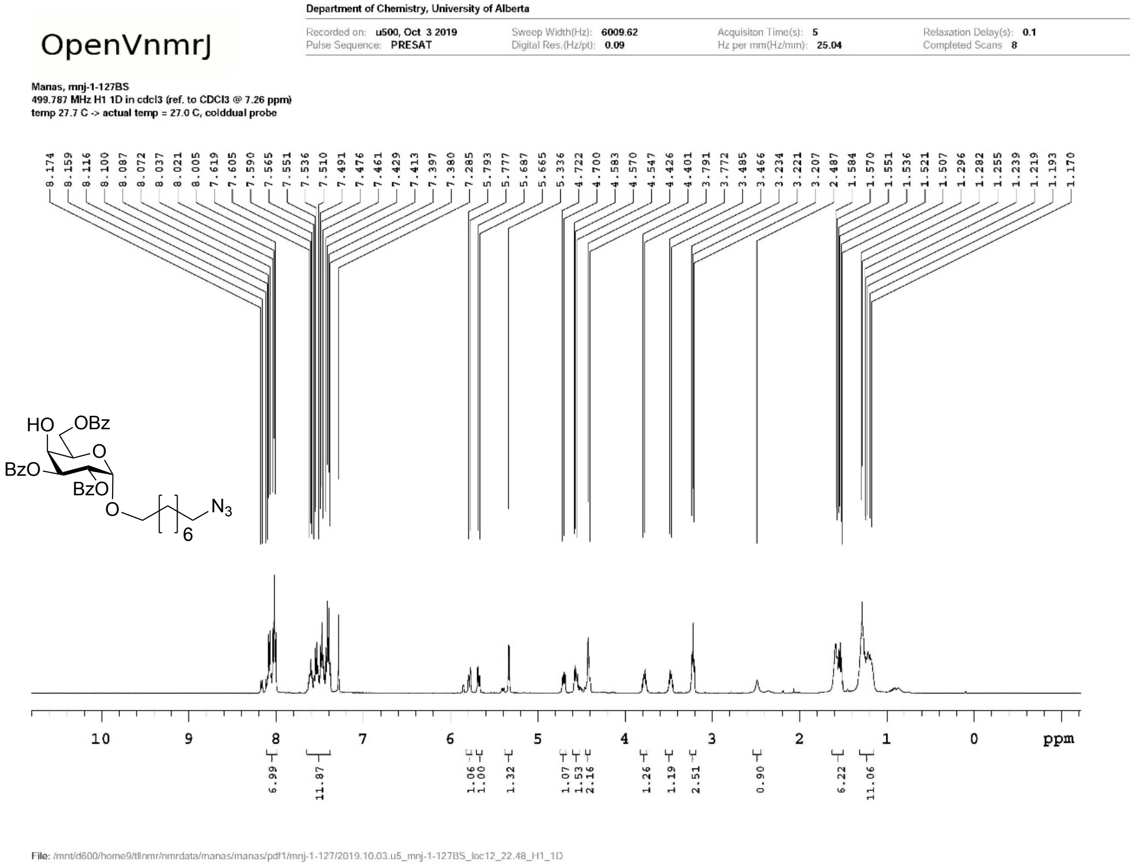

^{13}C NMR spectrum of compound **S4**

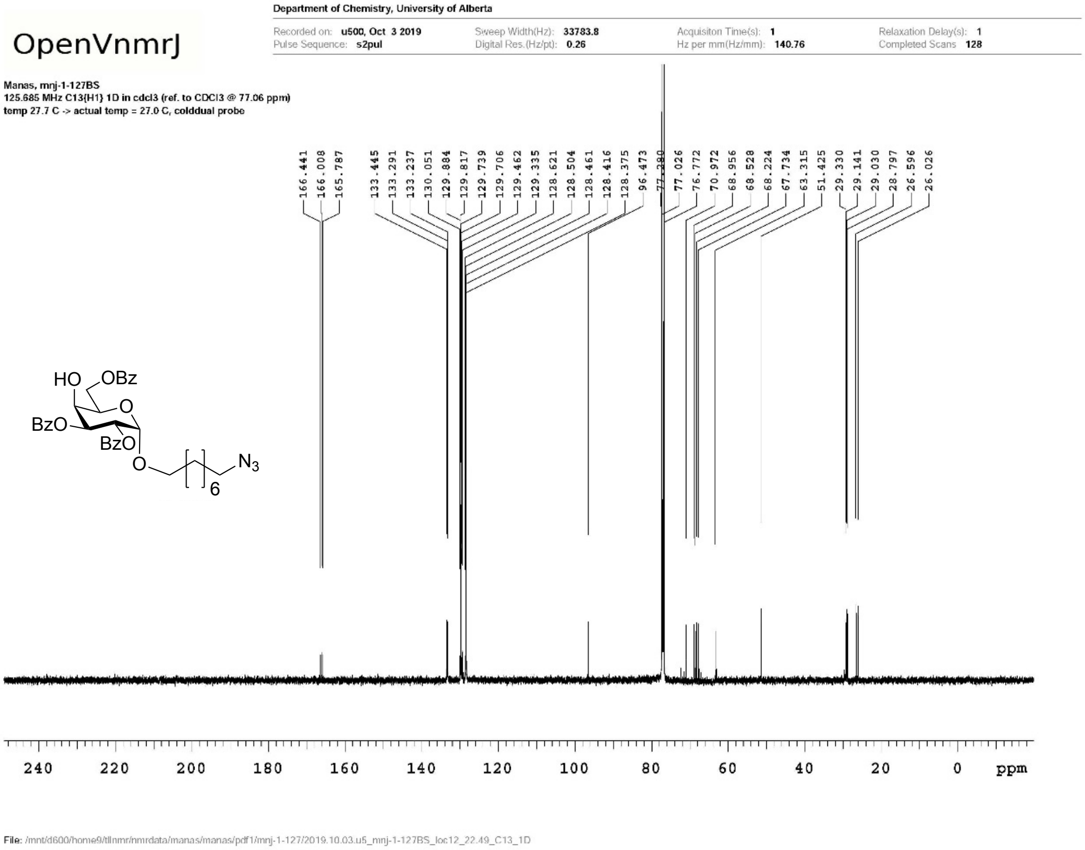

^{1}H NMR spectrum of compound **S5**

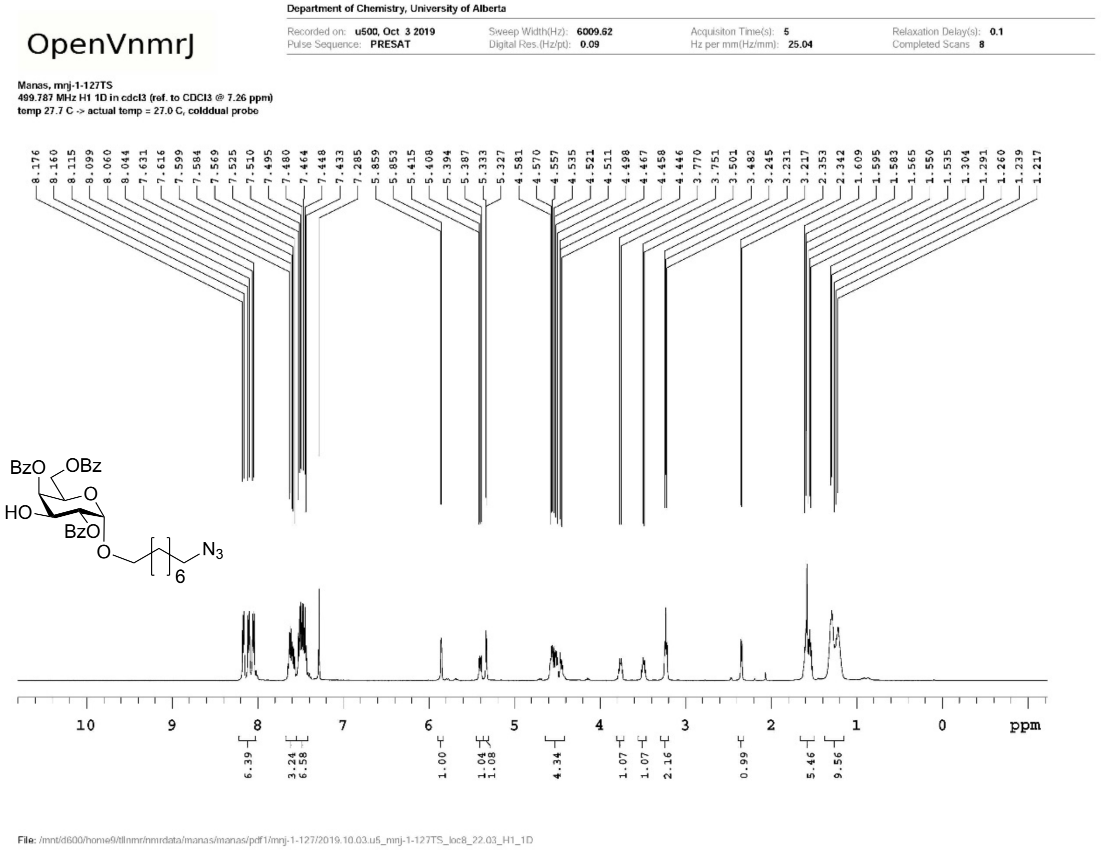

^{13}C NMR spectrum of compound **S5**

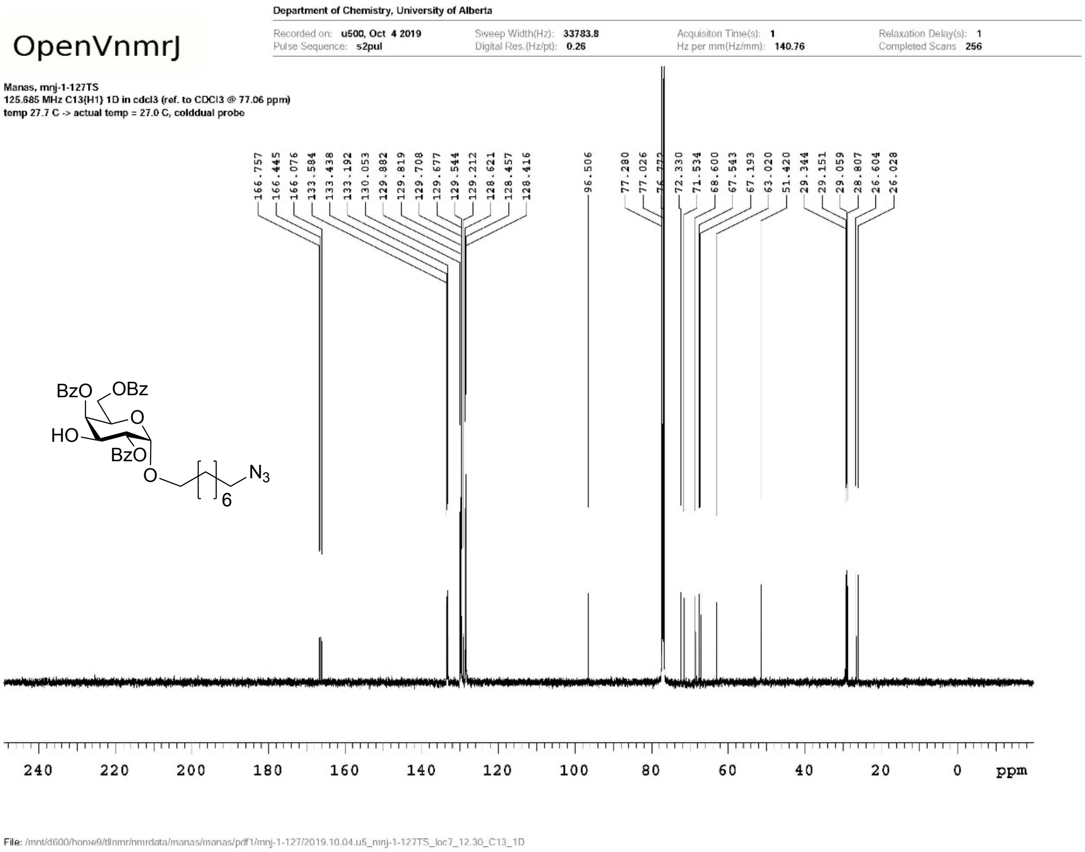

^1H NMR spectrum of compound **S7**

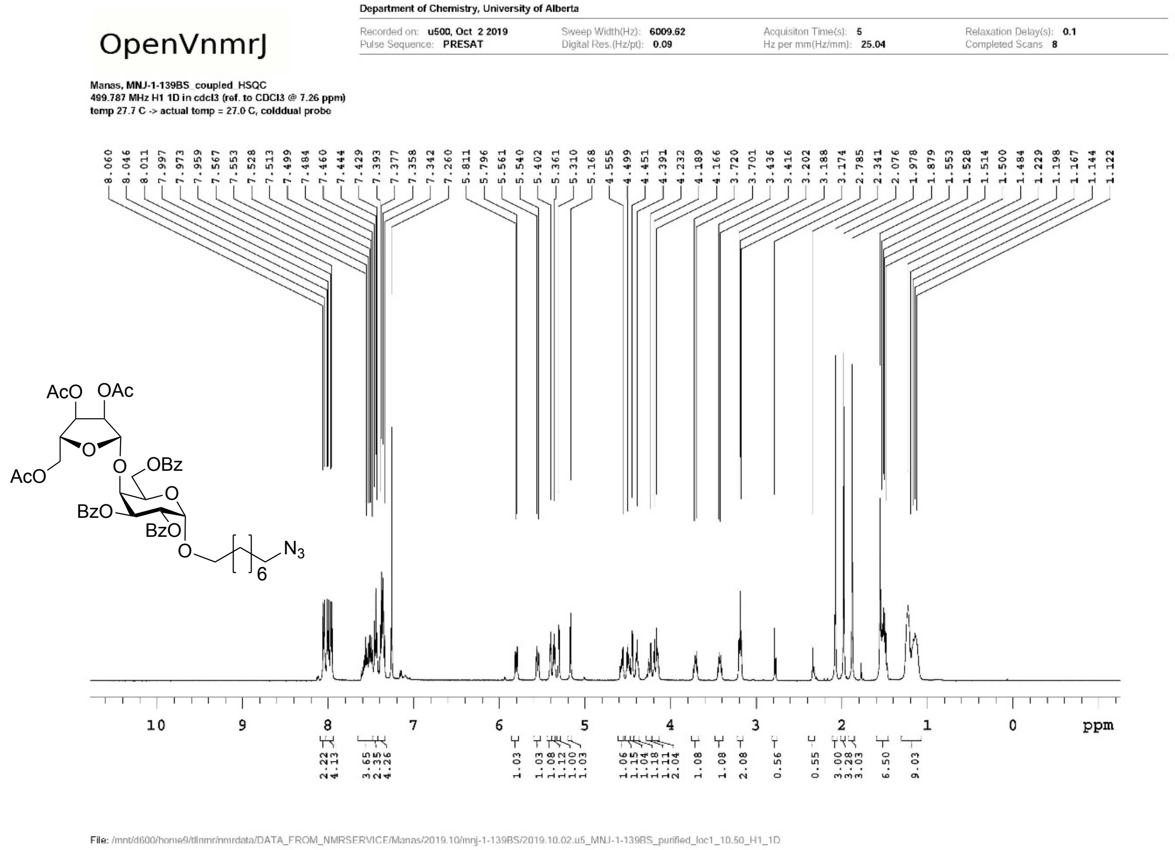

^{13}C NMR spectrum of compound **S7**

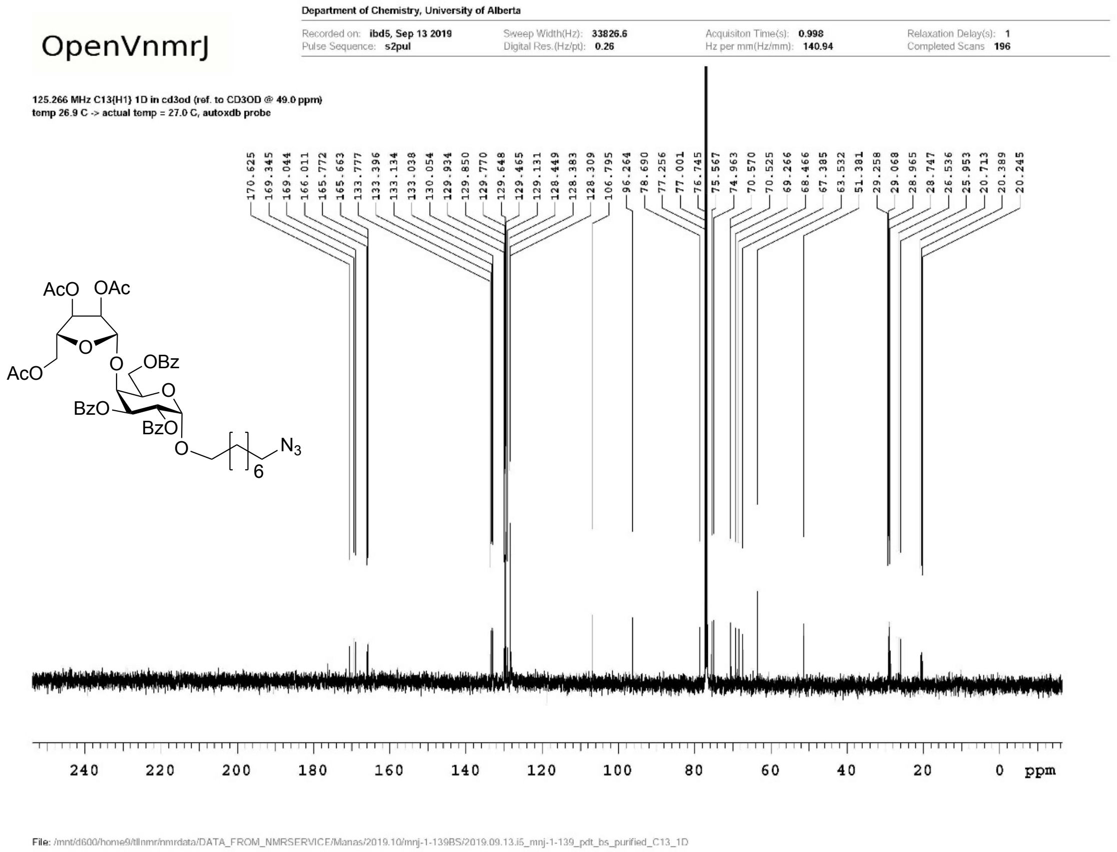

^{1}H NMR spectrum of compound **1**

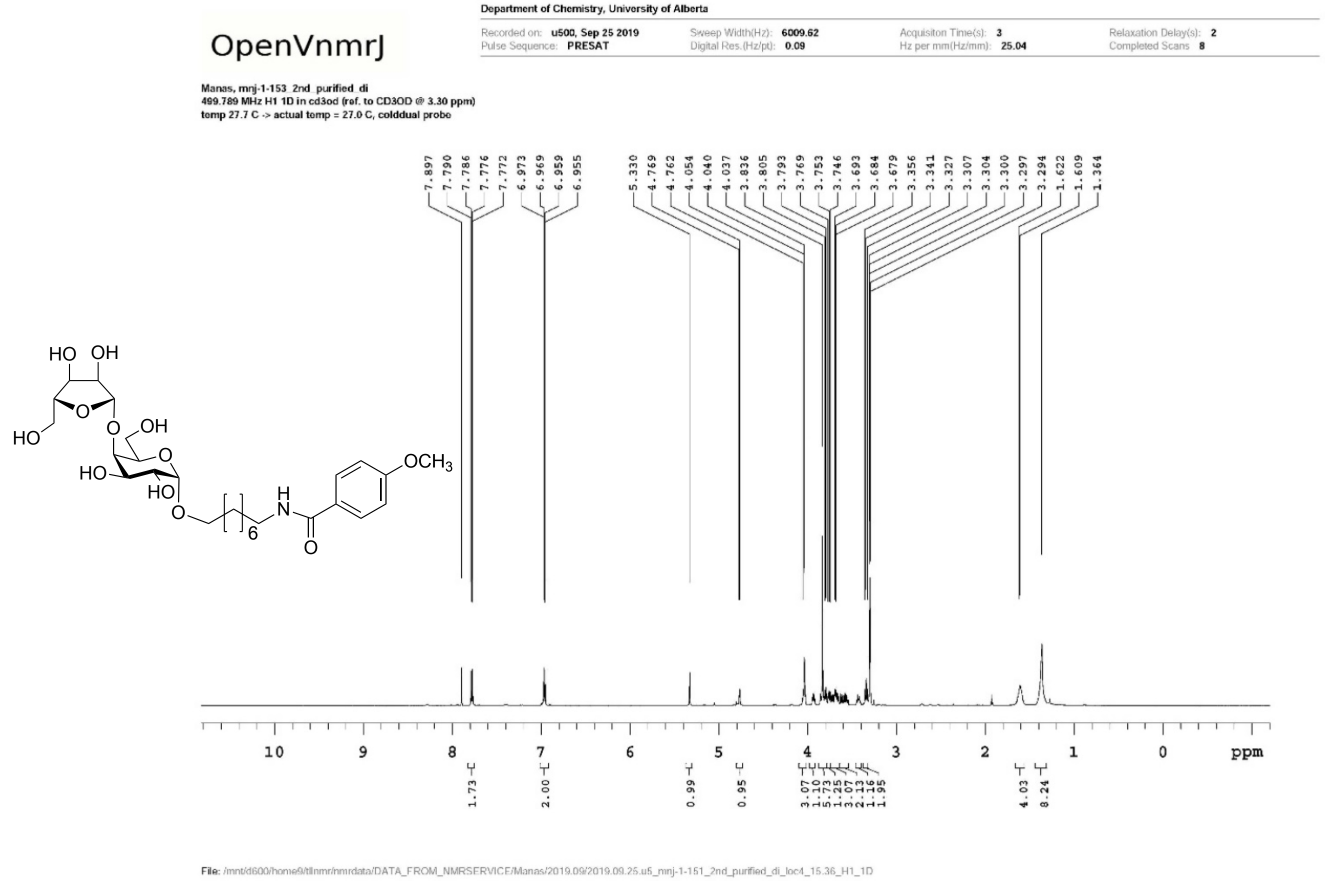

^{13}C NMR spectrum of compound **1**

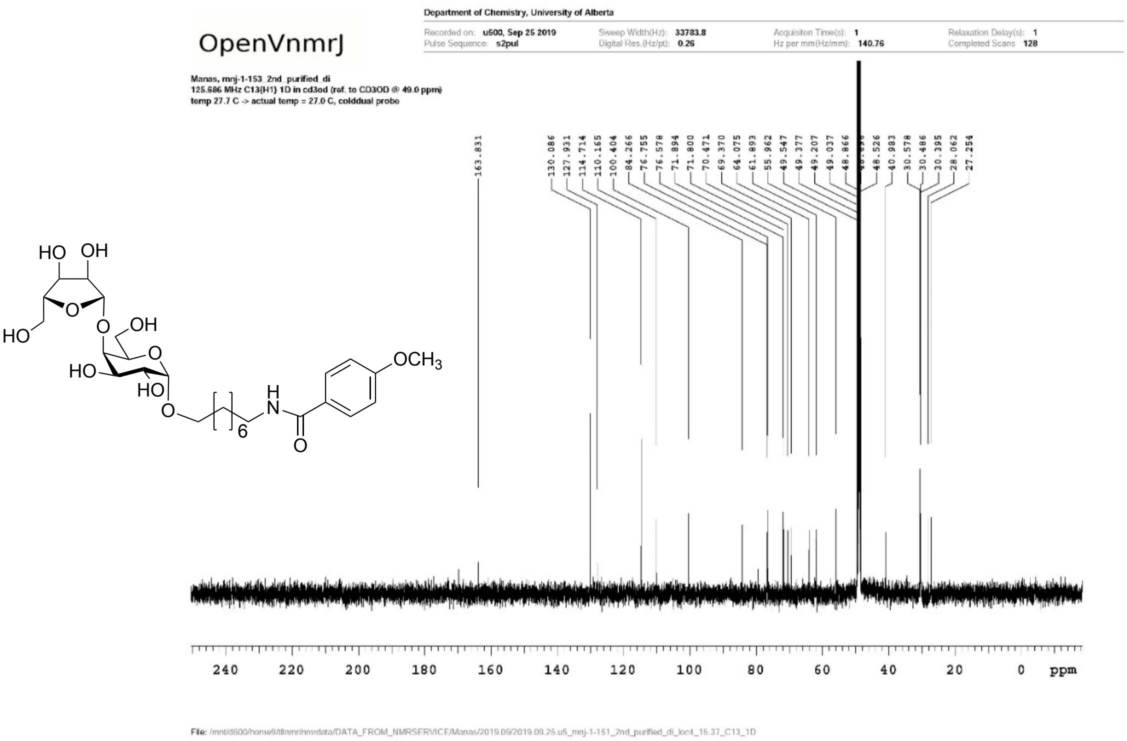

^{1}H NMR spectrum of compound **S9**

Department of Chemistry, University of Alberta

Agilent Technologies

Recorded on: u500, Jul 18 2018	Sweep Width(Hz): 6009.62	Acquisiton Time(s): 5	Relaxation Delay(s): 0.1
Pulse Sequence: PRESAT	Digital Res.(Hz/pt): 0.09	Hz per mm(Hz/mm): 25.04	Completed Scans 8

Rebecca, RK-2-24cc
499.797 MHz H1 1D in cdcl3 (ref. to CDCl3 @ 7.26 ppm)
temp 27.7 C -> actual temp = 27.0 C, colddual probe

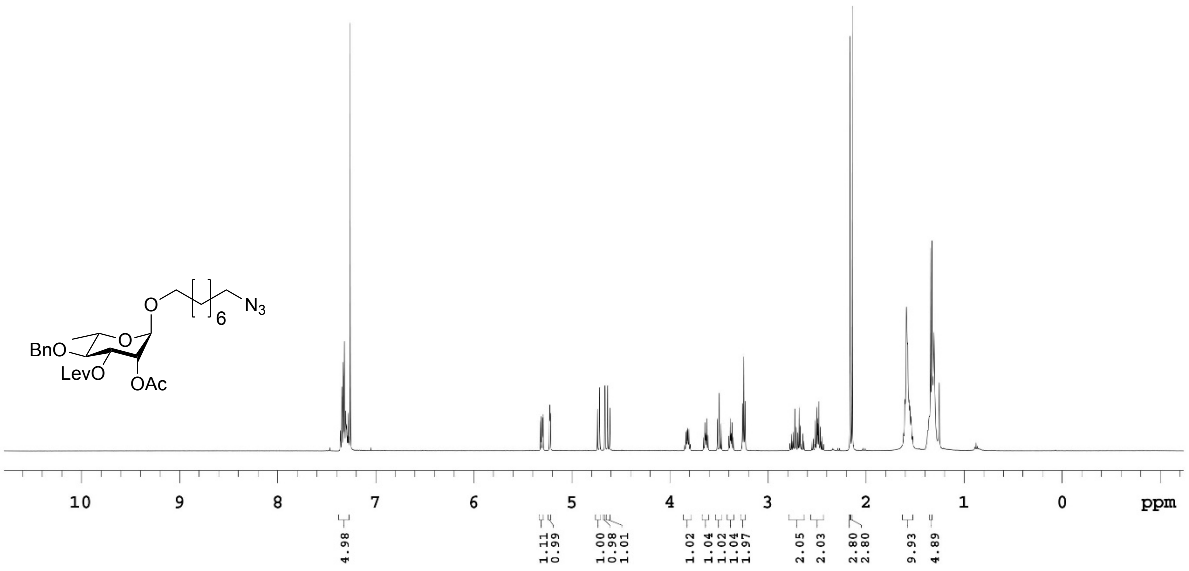

File: /mnt/d600/home9/tllnmr/nmrdata/DATA_FROM_NMRSERVICE/Rebecca/2018.07/2018.07.18.u5_RK-2-24cc_loc6_20.02_H1_1D

^{13}C NMR spectrum of compound **S9**

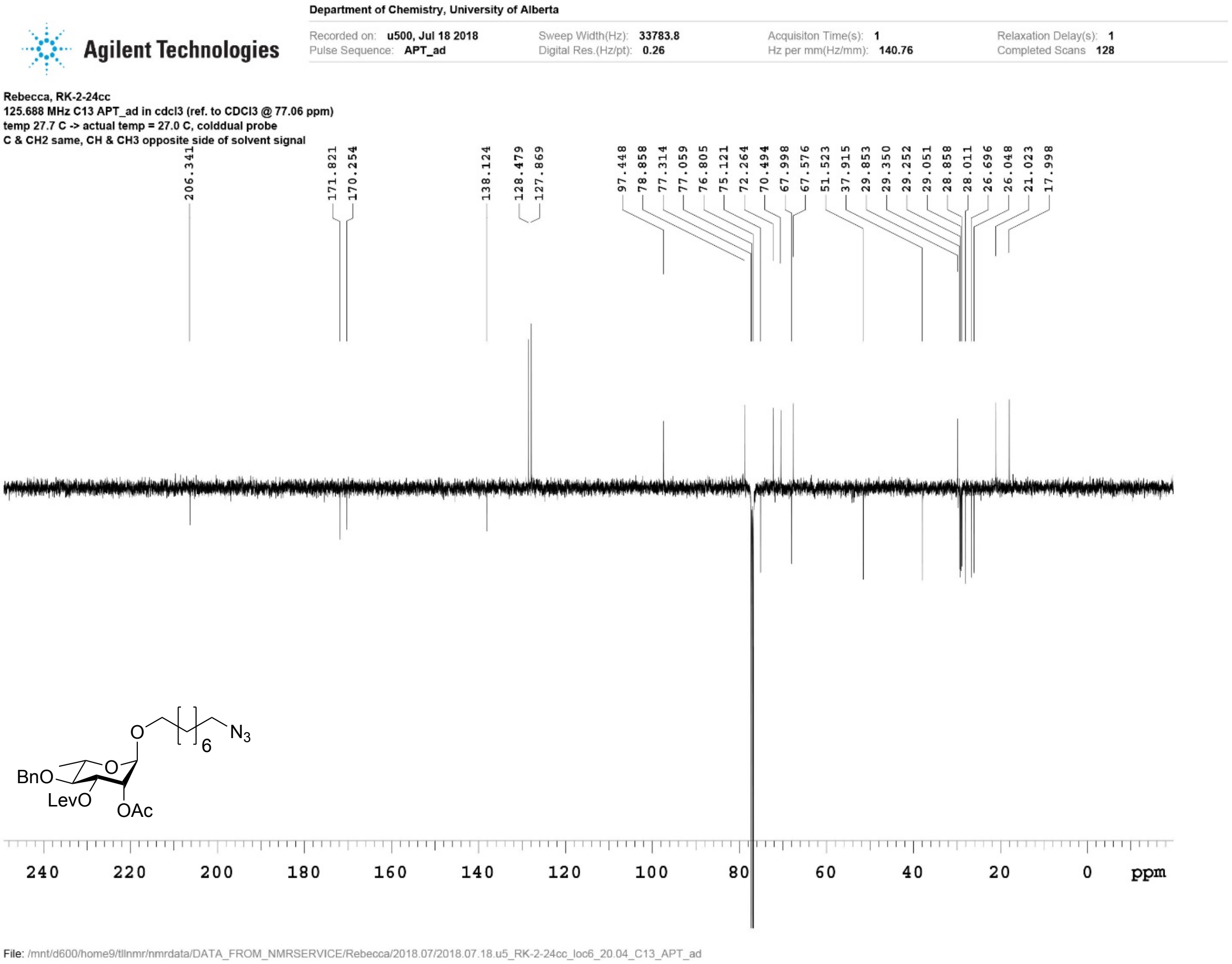

^{1}H NMR spectrum of compound **S10**

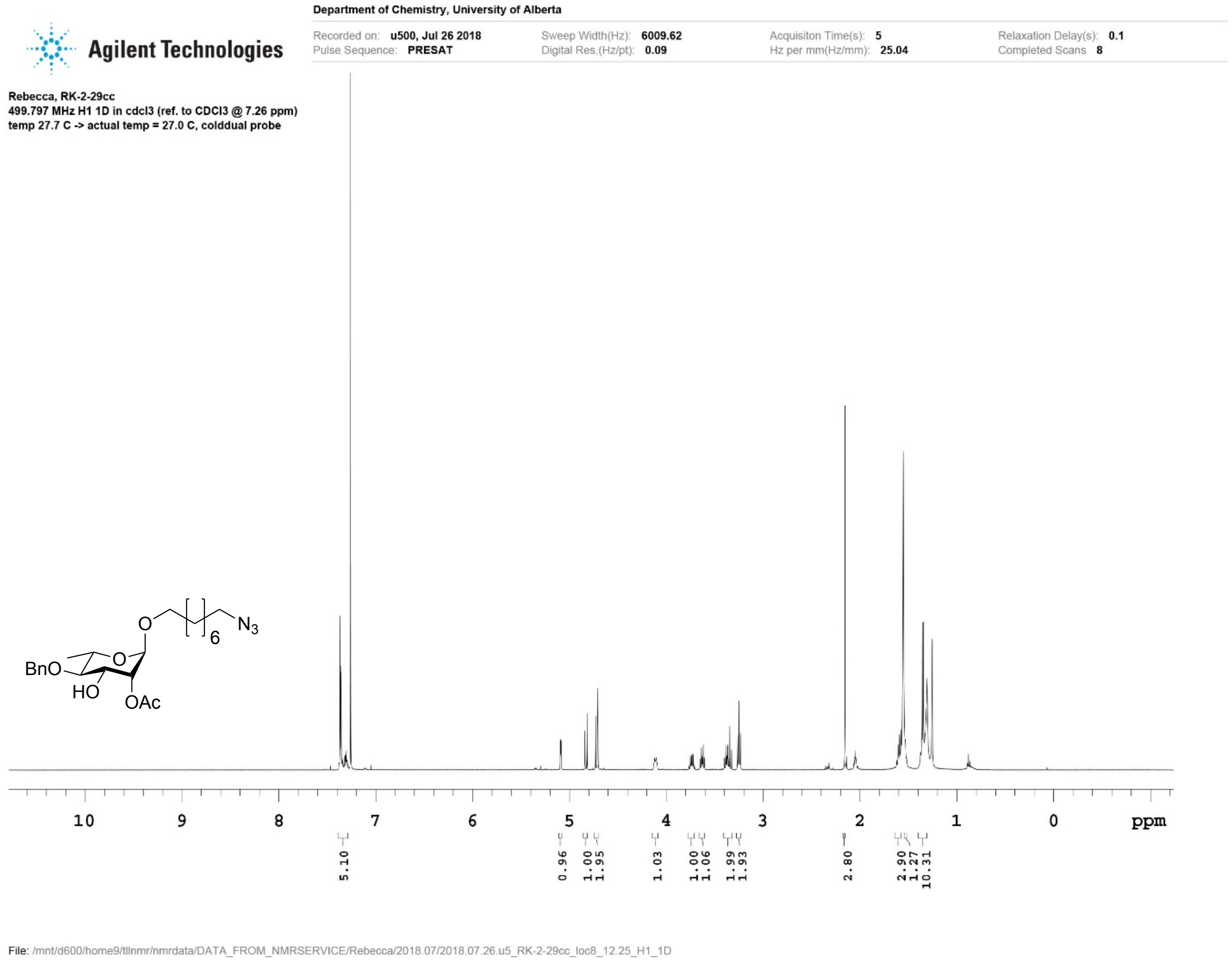

^{13}C NMR spectrum of compound **S10**

Department of Chemistry, University of Alberta

Agilent Technologies

Recorded on: ibd5, Aug 2 2018	Sweep Width(Hz): 33826.6	Acquisiton Time(s): 0.998	Relaxation Delay(s): 1
Pulse Sequence: APT_ad	Digital Res.(Hz/pt): 0.26	Hz per mm(Hz/mm): 140.94	Completed Scans 56

125.266 MHz C13 APT_ad in cdcl3 (ref. to CDCl3 @ 77.06 ppm)
temp 26.9 C -> actual temp = 27.0 C, autoxdb probe
C & CH2 same, CH & CH3 opposite side of solvent signal

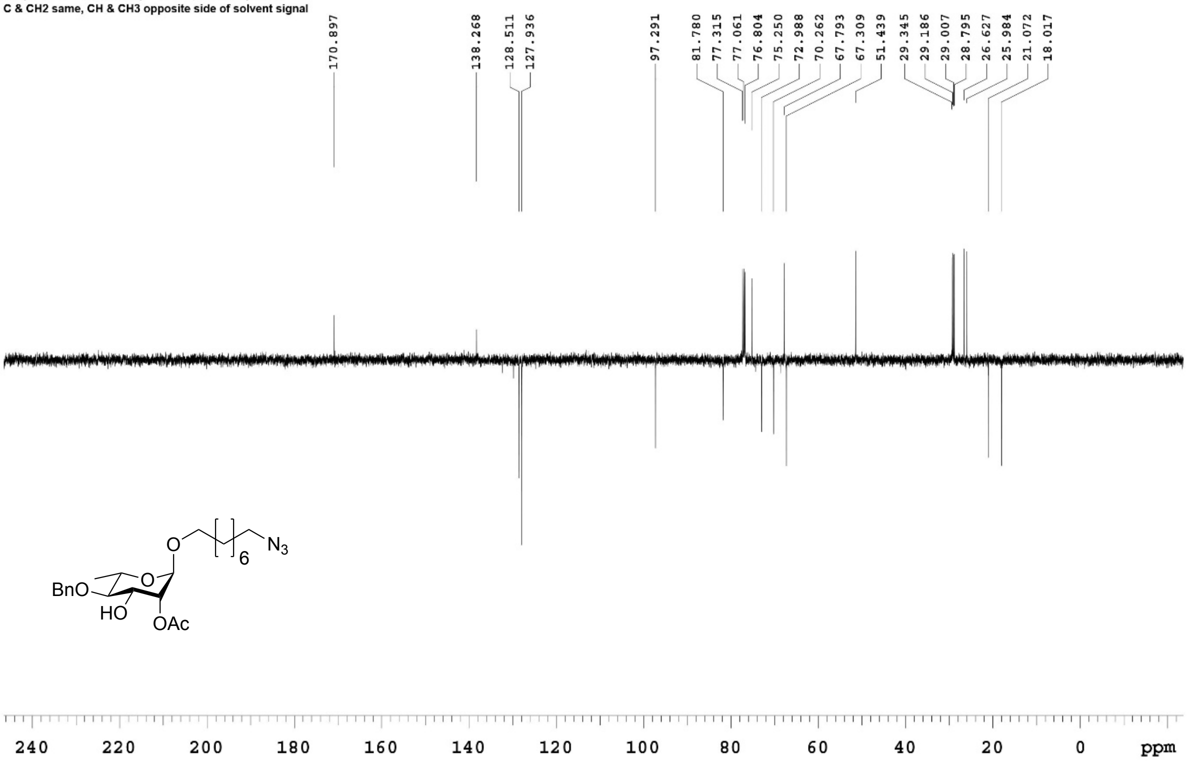

File: /mnt/d600/home9/tllnmr/nmrdata/DATA_FROM_NMRSERVICE/Rebecca/2018.07/2018.08.02.i5_RK-2-33_C13_APT_ad

^{1}H NMR spectrum of compound **S11**

Department of Chemistry, University of Alberta

Recorded on: **u500, Jul 31 2018**	Sweep Width(Hz): **6009.62**	Acquisiton Time(s): **5**	Relaxation Delay(s): **0.1**
Pulse Sequence: **PRESAT**	Digital Res.(Hz/pt): **0.09**	Hz per mm(Hz/mm): **25.04**	Completed Scans **8**

Rebecca, RK-2-31cc
499.797 MHz H1 1D in cdcl3 (ref. to CDCl3 @ 7.26 ppm)
temp 27.7 C -> actual temp = 27.0 C, colddual probe

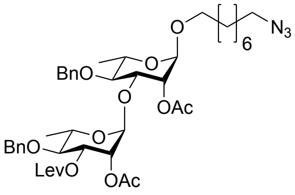

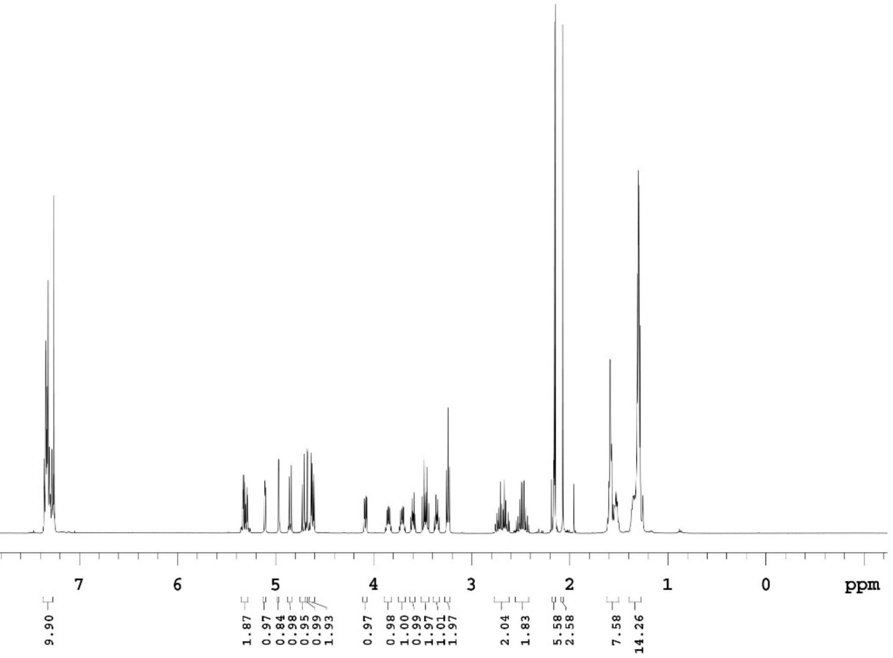

File: /mnt/d600/home9/tllnmr/nmrdata/DATA_FROM_NMRSERVICE/Rebecca/2018.07/2018.07.31.u5_RK-2-31cc_loc11_01.59_H1_1D

^{13}C NMR spectrum of compound **S11**

Department of Chemistry, University of Alberta

Recorded on: u500, Jul 31 2018	Sweep Width(Hz): 33783.8	Acquisiton Time(s): 1	Relaxation Delay(s): 1
Pulse Sequence: APT_ad	Digital Res.(Hz/pt): 0.26	Hz per mm(Hz/mm): 140.76	Completed Scans 808

Rebecca, RK-2-31cc
125.688 MHz C13 APT_ad in cdcl3 (ref. to CDCl3 @ 77.06 ppm)
temp 27.7 C -> actual temp = 27.0 C, colddual probe
C & CH2 same, CH & CH3 opposite side of solvent signal

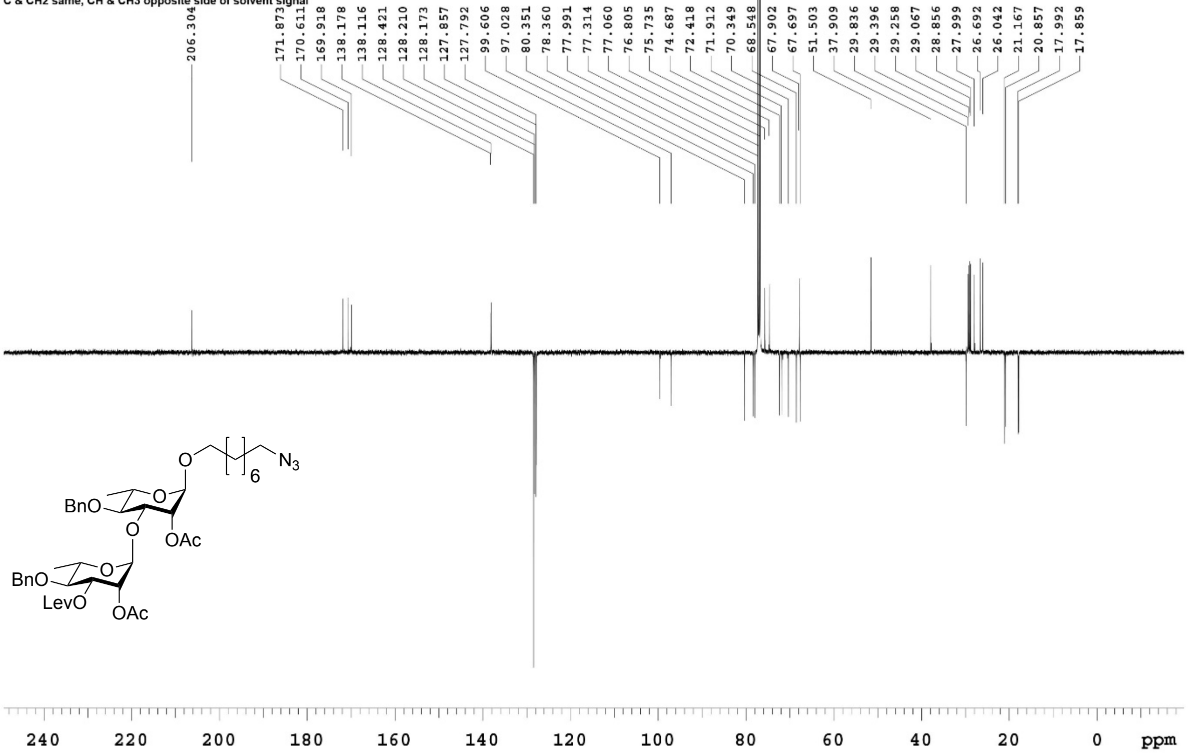

File: /mnt/d600/home9/tllnmr/nmrdata/DATA_FROM_NMRSERVICE/Rebecca/2018.07/2018.07.31.u5_RK-2-31cc_loc11_02.01_C13_APT_ad

^{1}H NMR spectrum of compound **S12**

Department of Chemistry, University of Alberta

Recorded on: u500, Aug 10 2018 | Sweep Width(Hz): 6009.62 | Acquisiton Time(s): 5 | Relaxation Delay(s): 0.1
Pulse Sequence: PRESAT | Digital Res.(Hz/pt): 0.09 | Hz per mm(Hz/mm): 25.04 | Completed Scans 8

Rebecca, RK-2-40c
499.797 MHz H1 1D in cdcl3 (ref. to CDCl3 @ 7.26 ppm)
temp 27.7 C -> actual temp = 27.0 C, colddual probe

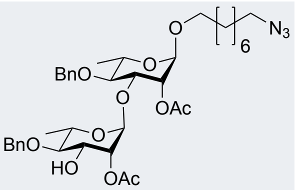

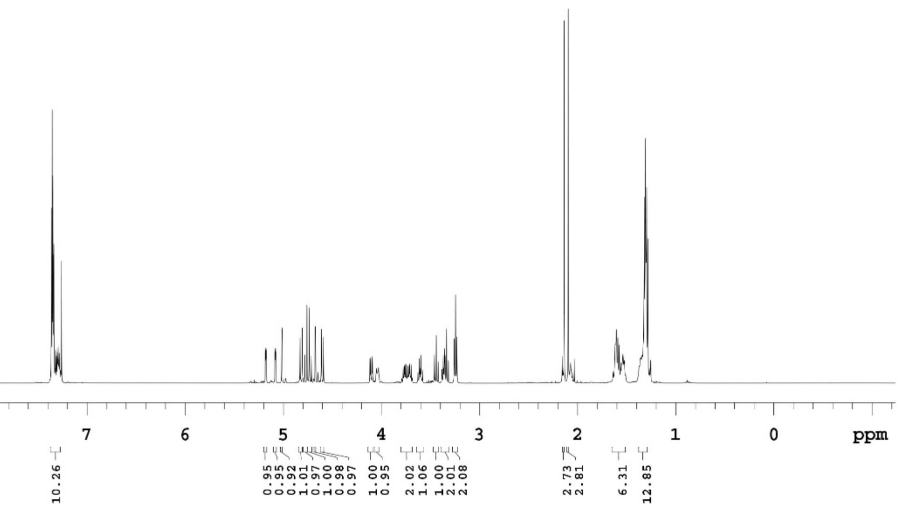

File: /mnt/d600/home9/tllnmr/nmrdata/DATA_FROM_NMRSERVICE/Rebecca/2018.08/2018.08.10.u5_RK-2-40c_loc3_18.18_H1_1D

^{13}C NMR spectrum of compound **S12**

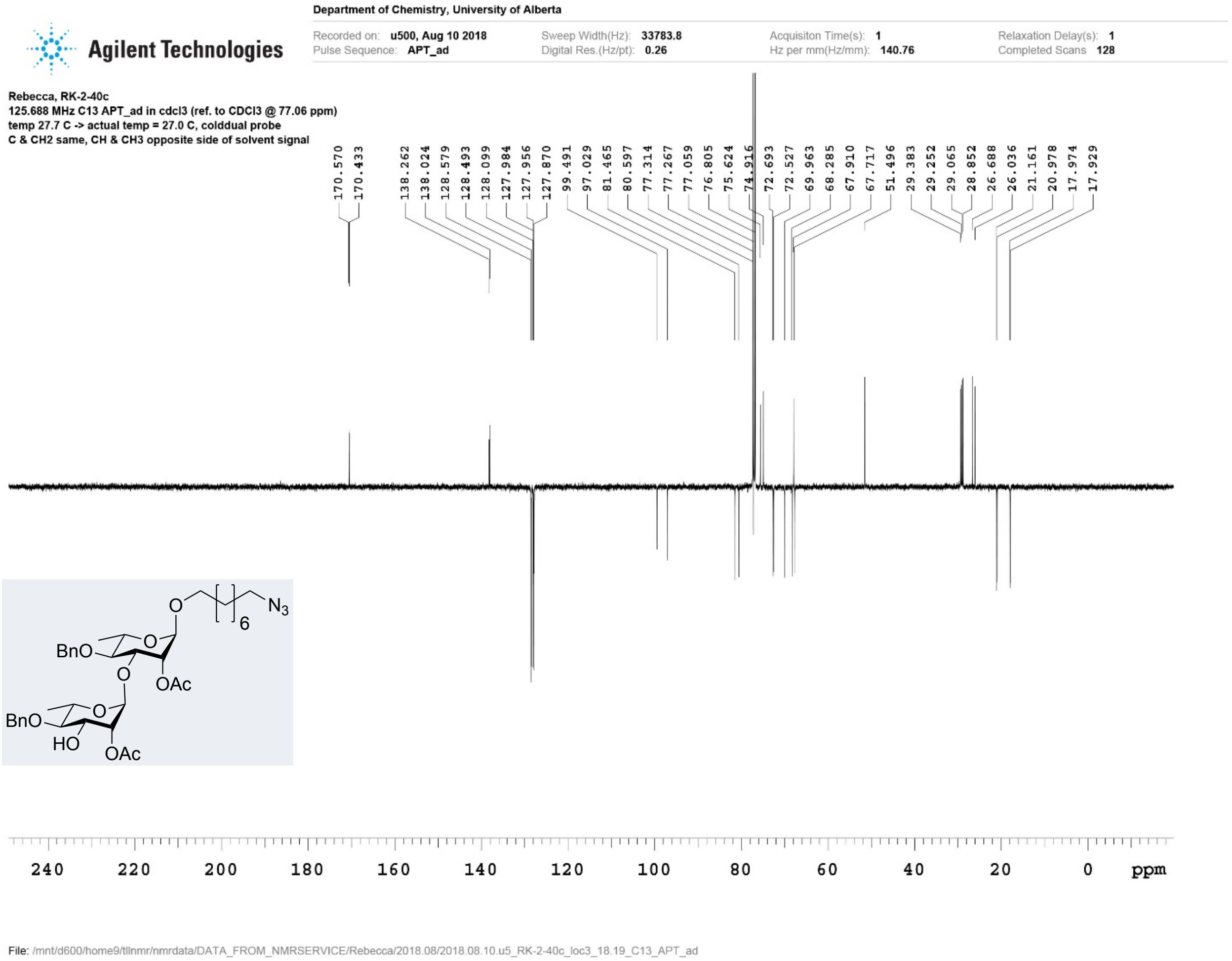

^{1}H NMR spectrum of compound **S14**

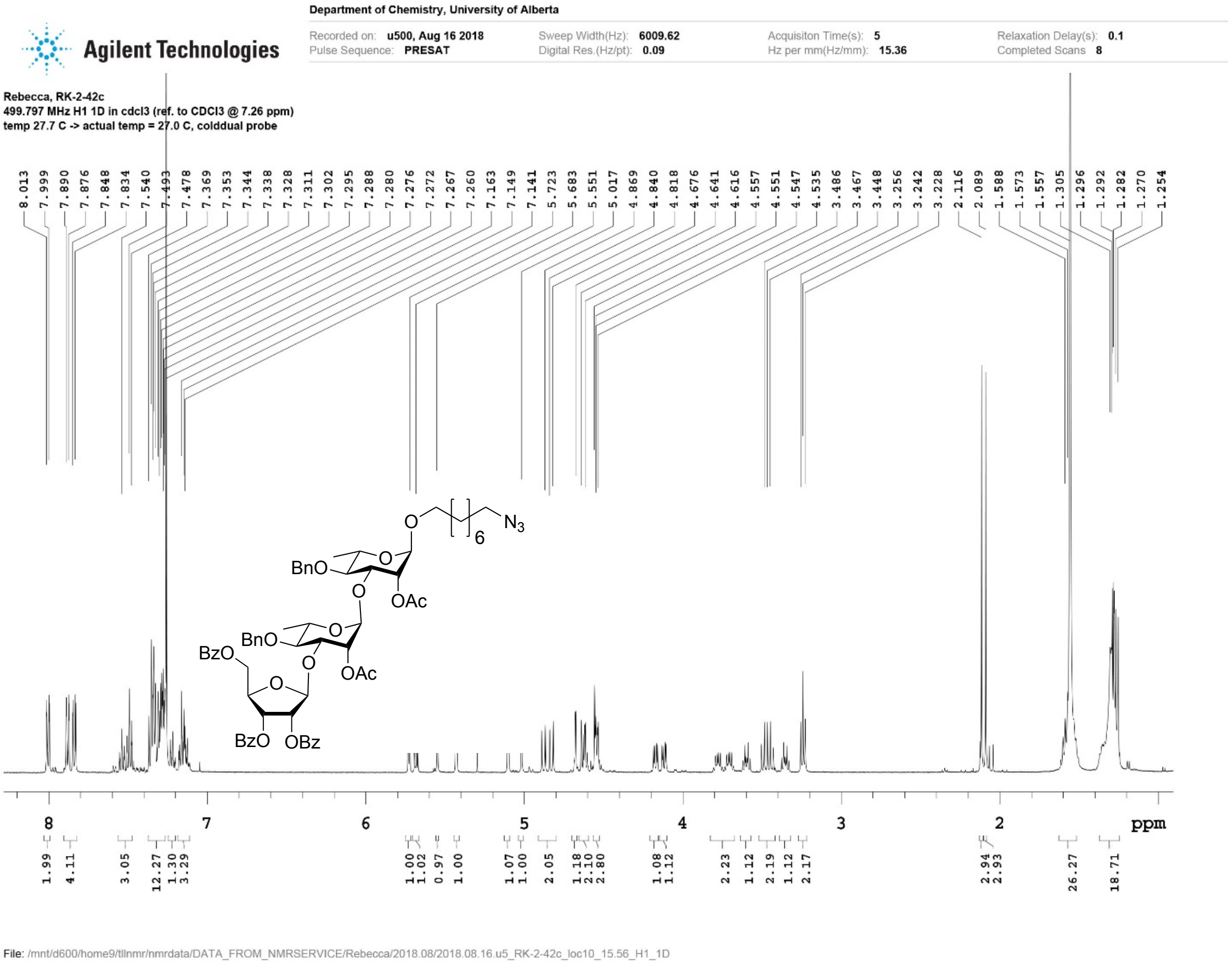

^{13}C NMR spectrum of compound **S14**

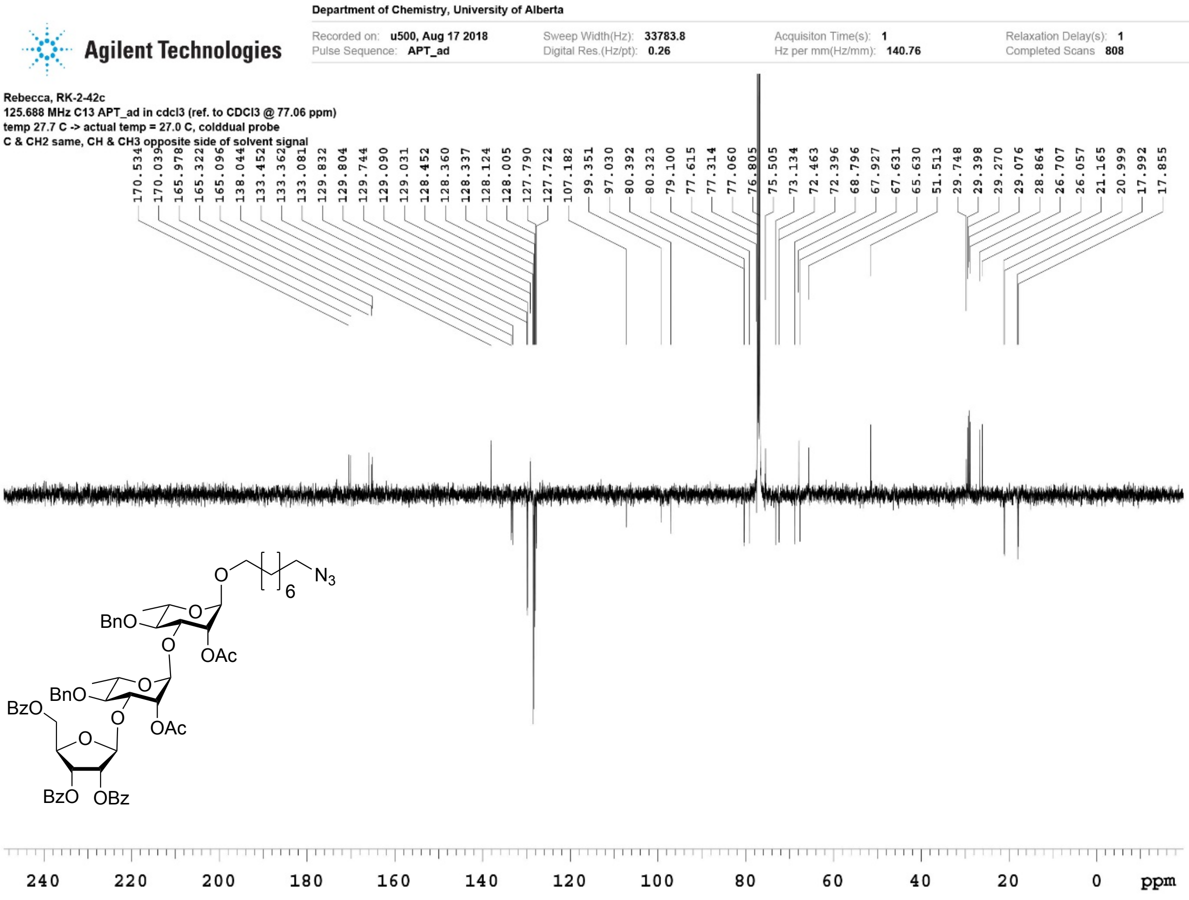

File: /mnt/d600/home9/tllnmr/nmrdata/DATA_FROM_NMRSERVICE/Rebecca/2018.08/2018.08.17.u5_RK-2-42c_loc9_01.48_C13_APT_ad

^{1}H NMR spectrum of compound **S15**

Department of Chemistry, University of Alberta

Agilent Technologies

Recorded on: u500, Apr 28 2019 | Sweep Width(Hz): 6009.62 | Acquisiton Time(s): 3 | Relaxation Delay(s): 2
Pulse Sequence: PRESAT | Digital Res.(Hz/pt): 0.09 | Hz per mm(Hz/mm): 25.04 | Completed Scans 16

Narasimha, NT-RK-Trisac-F-Pure
499.789 MHz H1 1D in cd3od (ref. to CD3OD @ 3.30 ppm)
temp 27.7 C -> actual temp = 27.0 C, colddual probe

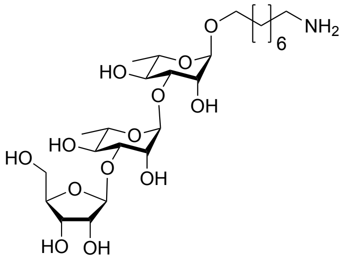

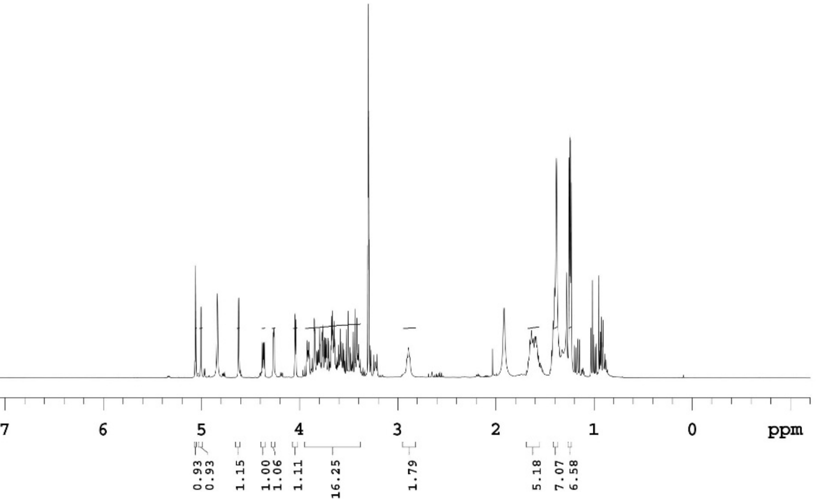

File: /mnt/d600/home9/tllnmr/nmrdata/DATA_FROM_NMRSERVICE/Narasimha/2019.04/2019.04.28.u5_NT-RK-Trisac-F-Pure_loc5_21.39_H1_1D

^{13}C NMR spectrum of compound **S15**

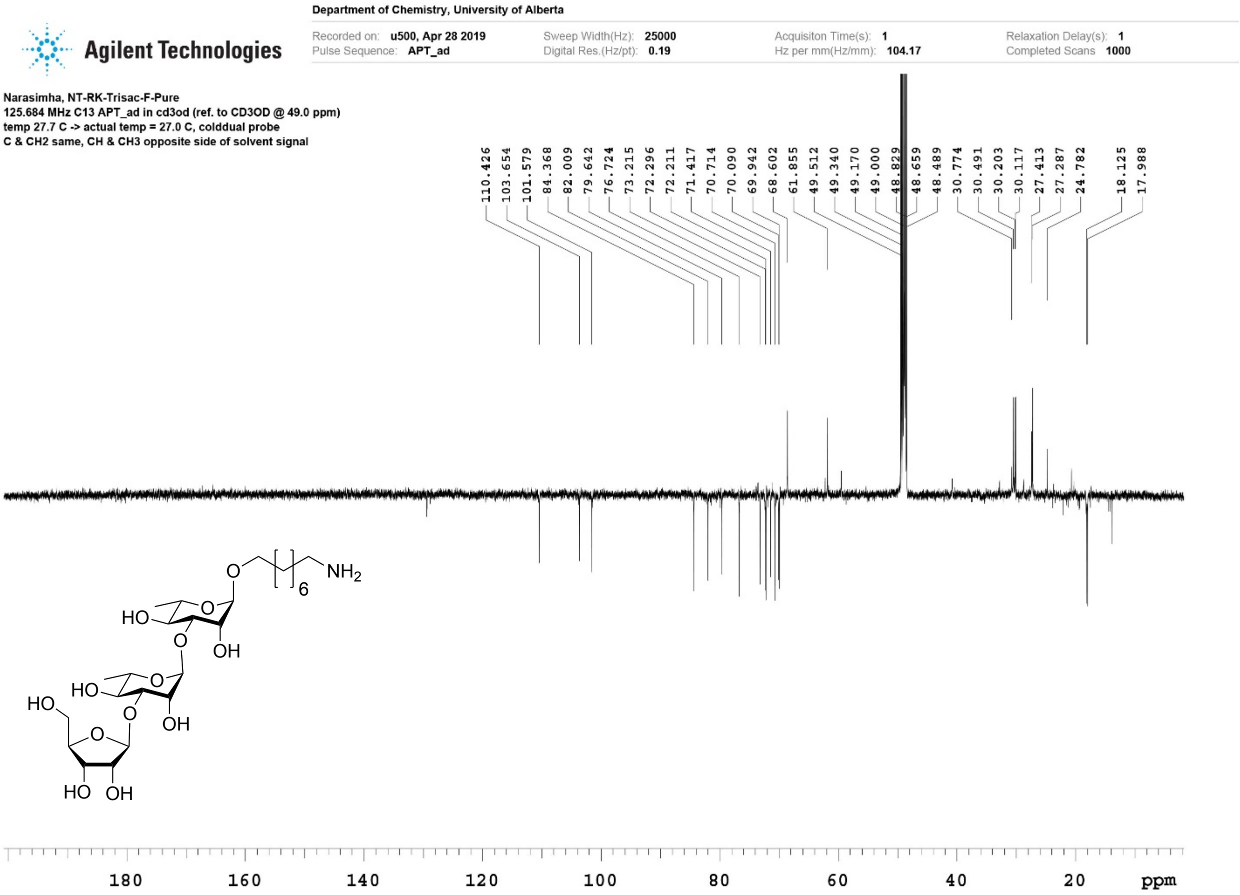

File: /mnt/d600/home9/tllnmr/nmrdata/DATA_FROM_NMRSERVICE/Narasimha/2019.04/2019.04.28.u5_NT-RK-Trisac-F-Pure_loc5_21.41_C13_APT_ad

^{1}H NMR spectrum of compound **4**

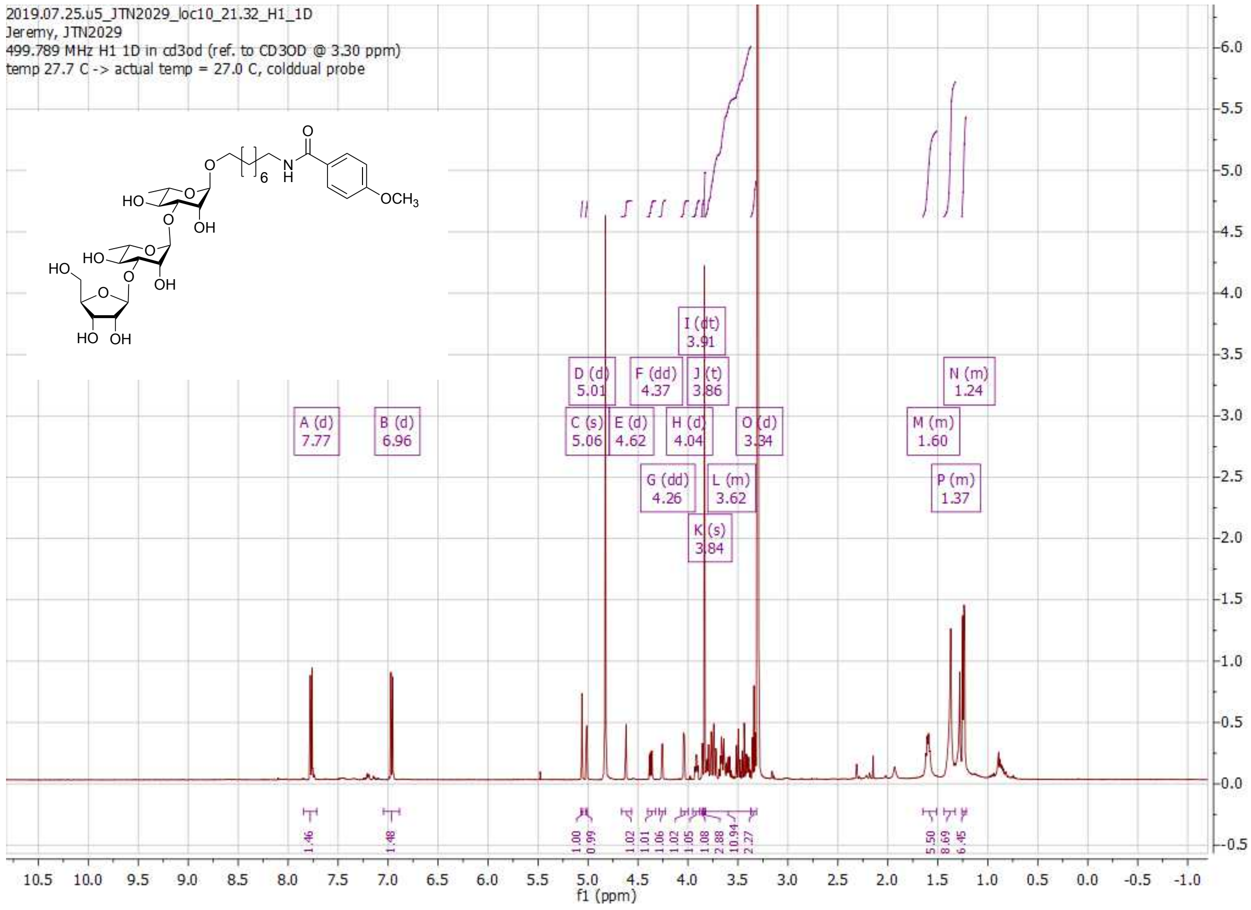

^{13}C NMR spectrum of compound **4**

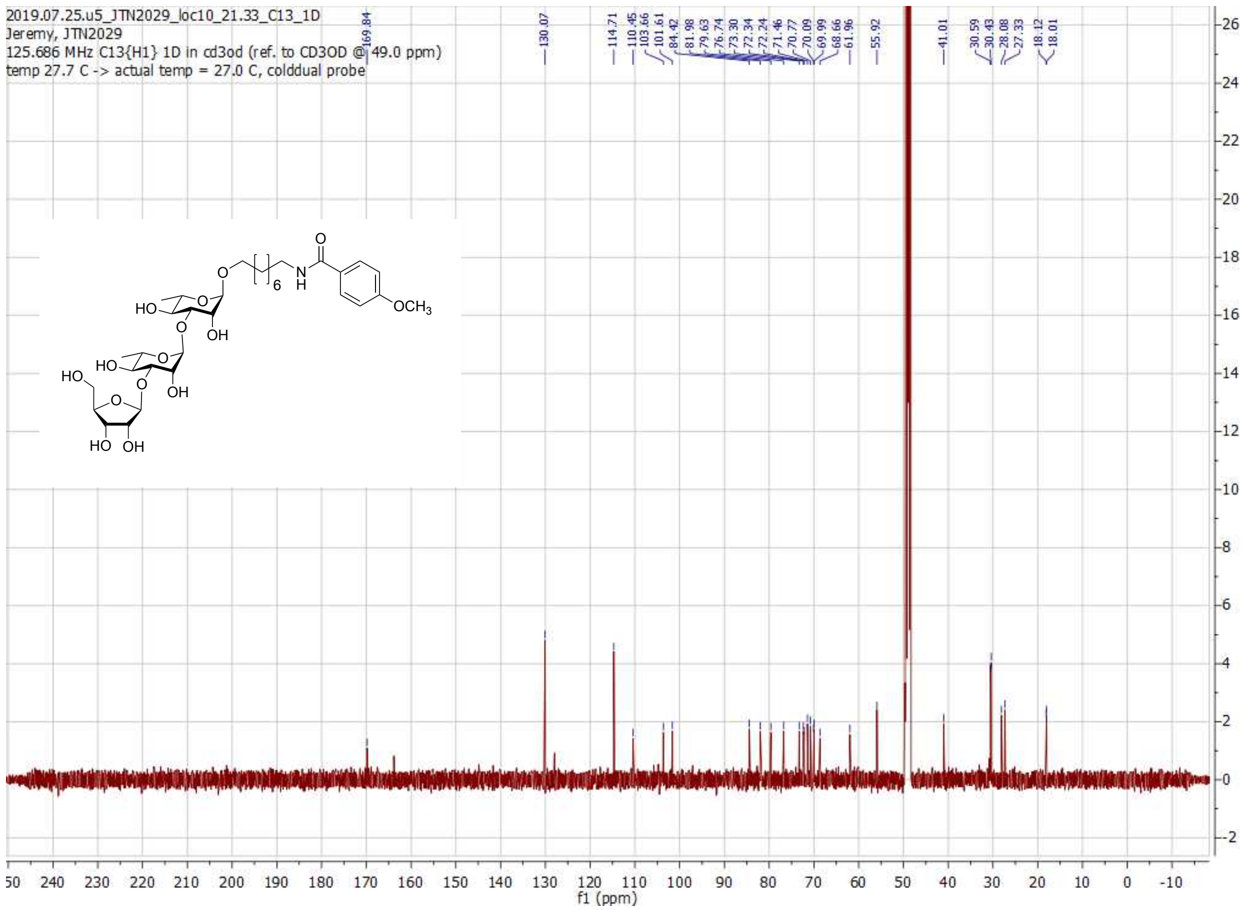

References

1. Bethesda Research Laboratories (1986) BRL pUC host: *E. coli* DH5αTM competent cells. *Focus (Madison)*. **8**, 9–12
2. Mann, E., Ovchinnikova, O. G., King, J. D., and Whitfield, C. (2015) Bacteriophage-mediated glucosylation can modify lipopolysaccharide O-antigens synthesized by an ATP-binding cassette (ABC) transporter-dependent assembly mechanism. *J Biol Chem*. **290**, 25561
3. Köplin, R., Brisson, J.-R., and Whitfield, C. (1997) UDP-galactofuranose precursor required for formation of the lipopolysaccharide O antigen of *Klebsiella pneumoniae* serotype O1 is synthesized by the product of the *rfbD* KPO1 gene. *J. Biol. Chem.* **272**, 4121–4128
4. Chang, A. C., and Cohen, S. N. (1978) Construction and characterization of amplifiable multicopy DNA cloning vehicles derived from the P15A cryptic miniplasmid. *J. Bacteriol.* **134**, 1141–1156
5. Guzman, L. M., Belin, D., Carson, M. J., and Beckwith, J. (1995) Tight regulation, modulation, and high-level expression by vectors containing the arabinose PBAD promoter. *J. Bacteriol.* **177**, 4121–4130
6. Greenfield, L. K., Richards, M. R., Vinogradov, E., Wakarchuk, W. W., Lowary, T. L., and Whitfield, C. (2012) Domain organization of the polymerizing mannosyltransferases involved in synthesis of the *Escherichia coli* O8 and O9a lipopolysaccharide O-antigens. *J. Biol. Chem.* **287**, 38135–38149
7. Karimova, G., Pidoux, J., Ullmann, A., and Ladant, D. (1998) A bacterial two-hybrid system based on a reconstituted signal transduction pathway. *Proc. Natl. Acad. Sci.* **95**, 5752–6
8. Kos, V., and Whitfield, C. (2010) A membrane-located glycosyltransferase complex required for biosynthesis of the D-Galactan I lipopolysaccharide O antigen in *Klebsiella pneumoniae*. *Journal of Biological Chemistry*. **285**, 19668–19687
9. Kelly, S. D., Williams, D. M., Nothof, J. T., Kim, T., Lowary, T. L., Kimber, M. S., and Whitfield, C. (2022) The biosynthetic origin of ribofuranose in bacterial polysaccharides. *Nat. Chem. Bio.* **18**, 530–537
10. Kelly, S. D., Clarke, B. R., Ovchinnikova, O. G., Sweeney, R. P., Williamson, M. L., Lowary, T. L., and Whitfield, C. (2019) *Klebsiella pneumoniae* O1 and O2ac antigens provide prototypes for an unusual strategy for polysaccharide antigen diversification. *J. Biol. Chem.* **294**, 10863–10876
11. Pinto, A., Ciesla, J. H., Palucci, A., Sutliff, B. P., and Nomura, C. T. (2016) Chemically intractable no more: In vivo incorporation of “click”-ready fatty acids into poly-[(R)-3-hydroxyalkanoates] in *Escherichia coli*. *ACS Macro Lett*. **5**, 215–219
12. Kurosu, M., and Li, K. (2008) Highly efficient O-glycosylations with p-tolyl thioribosides and p-TolSOTf. *Journal of Organic Chemistry*. **73**, 9767–9770
